# Integration of lipidomic and polygenic risk scores within contemporary clinical cardiovascular risk assessment pathways: a multi-cohort development and validation study

**DOI:** 10.1016/j.eclinm.2026.104159

**Published:** 2026-08-24

**Authors:** Aleksandar Dakic, Jingqin Wu, Tingting Wang, Thomas G. Meikle, Kevin Huynh, Changyu Yi, Habtamu B. Beyene, Agus Salim, Michelle Cinel, Nat Mellett, Thy Duong, Alexandra N. Faulkner, Matilda van Buuren-Milne, Anjali Bhagwat, Jonathan E. Shaw, Dianna J. Magliano, Gerald F. Watts, Joseph Hung, Jennie Hui, John P. Beilby, John Blangero, Eric K. Moses, Melissa C. Southey, Roger L. Milne, Allison M. Hodge, John J. McNeil, Paul Lacaze, Chenglong Yu, Rory Wolfe, Jean YH. Yang, Stuart M. Grieve, Clara K. Chow, Stephen T. Vernon, Michael P. Gray, Gemma A. Figtree, Jedidiah I. Morton, Paul Scuffham, Mark Woodward, Lisa Kalman, Ellie Paige, Melinda J. Carrington, Michael Inouye, Corey Giles, Peter J. Meikle

**Affiliations:** a Baker Heart and Diabetes Institute, Melbourne, Australia; b Baker Department of Cardiometabolic Health, Melbourne University, Melbourne, Australia; c Baker Department of Cardiovascular Research Translation and Implementation, La Trobe University, Melbourne, Australia; d Faculty of Medicine, Nursing and Health Sciences, Monash University, Melbourne, Australia; e Melbourne School of Population and Global Health, Melbourne University, Melbourne, Australia; f Medical School, University of Western Australia, Perth, Australia; g Lipid Disorders Clinic, Department of Cardiology, Royal Perth Hospital, Perth, Australia; h PathWest Laboratory Medicine of WA, Nedlands, Western Australia, Australia; i School of Population and Global Health, University of Western Australia, Crawley, Australia; j School of Biomedical Sciences, University of Western Australia, Perth, Australia; k South Texas Diabetes and Obesity Institute, The University of Texas Rio Grande Valley, Brownsville, TX, USA; l School of Biomedical Sciences, University of Western Australia, Crawley, Australia; m Menzies Institute for Medical Research, University of Tasmania, Hobart, Australia; n The School of Clinical Sciences, Monash Health, Faculty of Medicine, Nursing and Health Sciences, Monash University, Melbourne, Australia; o Cancer Epidemiology Division, Cancer Council Victoria, Melbourne, Australia; p School of Public Health and Preventive Medicine, Monash University, Melbourne, Australia; q School of Mathematics and Statistics, University of Sydney, Camperdown, Australia; r Charles Perkins Centre, University of Sydney, Camperdown, Australia; s Department of Radiology, Royal Prince Alfred Hospital, Camperdown, Australia; t Westmead Applied Research Centre, Faculty of Medicine and Health, University of Sydney, Westmead, Australia; u Department of Cardiology, Westmead Hospital, Westmead, Australia; v Kolling Institute of Medical Research, University of Sydney, St Leonards, Australia; w Department of Cardiology, Royal North Shore Hospital, St Leonards, Australia; x School of Medicine and Dentistry - Clinical Medicine, Griffith University, Gold Coast, Australia; y The George Institute for Global Health, University of New South Wales, Sydney, Australia; z The George Institute for Global Health, School of Public Health, Imperial College London, London, UK; aa National Heart Foundation of Australia, Melbourne office, Australia; ab QIMR Berghofer Medical Research Institute, Brisbane, Australia; ac Cambridge Baker Systems Genomics Initiative, Department of Public Health and Primary Care, University of Cambridge, Cambridge, United Kingdom; ad British Heart Foundation Cardiovascular Epidemiology Unit, Department of Public Health and Primary Care, University of Cambridge, Cambridge, United Kingdom; ae Health Data Research UK Cambridge, Wellcome Genome Campus and University of Cambridge, Cambridge, United Kingdom

**Keywords:** Cardiovascular disease, Risk prediction, Lipidomics, Polygenic risk score, Precision prevention

## Abstract

**Background:**

Guideline-recommended clinical risk scores such as AusCVDRisk underestimate cardiovascular disease (CVD) risk in a substantial proportion of individuals who later experience events, with up to 65% initially classified as low or intermediate risk. This limitation is most consequential in the intermediate-risk group, where treatment decisions are uncertain and additional risk refinement could alter management. Circulating lipid species and inherited genetic variation capture complementary molecular aspects of atherosclerotic risk that are not fully reflected by conventional clinical variables, but are not routinely incorporated into primary-care risk assessment. We investigated whether selective integration of lipidomic and genomic risk signals into AusCVDRisk improves 5-year CVD prediction and reclassification, with a focus on individuals at intermediate clinical risk.

**Methods:**

A lipidomic score comprising 689 lipid species measured by liquid chromatography–tandem mass spectrometry was derived using regularised Cox regression in 8082 participants from the Australian Diabetes, Obesity and Lifestyle Study (1999–2000). A genome-wide coronary artery disease polygenic score (PGS002048; 762,124 variants) was optimised in 3328 participants from the Busselton Health Study (1994–95). Each score was adjusted for AusCVDRisk predictors to isolate independent effects and incorporated into Cox models retaining the AusCVDRisk linear predictor as a fixed offset, generating lipidomic-enhanced (L.CVDRisk), genomic-enhanced (G.CVDRisk), and combined (LG.CVDRisk) scores. Internal and external validation was performed across five Australian cohorts totalling 13,521 adults without baseline CVD. Discrimination (Harrell’s concordance index; C-statistic), calibration, categorical net reclassification improvement (NRI), and decision-curve analyses were assessed.

**Findings:**

LG.CVDRisk showed modest gains in discrimination compared with AusCVDRisk (pooled ΔC among intermediate-risk individuals 0.071, 95% CI 0.033–0.109; overall 0.012, 95% CI 0.000–0.024). Risk classification improved substantially (pooled NRI in the intermediate-risk group 0.305, 95% CI 0.212–0.397; overall 0.080, 95% CI 0.031–0.129), with net event and non-event reclassification of 38.2% (95% CI 29.3–47.0%) and −6.8% (95% CI −9.3 to −4.2%) among intermediate-risk individuals. Decision-curve analysis showed the greatest net benefit when molecular profiling was selectively applied to individuals with intermediate AusCVDRisk (5–<10%). In a coronary imaging cohort, LG.CVDRisk reclassified 17 (41%) of 41 intermediate-risk individuals with extensive coronary calcification into the high-risk category.

**Interpretation:**

Selective augmentation of an established clinical risk algorithm with lipidomic and genomic information improves cardiovascular risk stratification among individuals at intermediate baseline risk. This approach supports targeted molecular testing within existing primary-care pathways to inform personalised prevention.

**Funding:**

National Heart Foundation, Australia, Australian Government Medical Research Future Fund, National Health and Medical Research Council, Victorian Government.

**Research in context**

**Evidence before this study**

We searched PubMed on 1 February 2025 using the terms “(lipidomic OR metabolomic) AND polygenic AND cardiovascular risk prediction” without language restrictions and reviewed reference lists of relevant articles. Although several studies have layered polygenic scores onto guideline-recommended clinical models, none integrated both molecularly resolved lipidomic and genomic components within an established, population-calibrated risk score with external validation. More recent analyses integrating metabolic and polygenic data into Systematic Coronary Risk Evaluation 2 (SCORE2) showed promising results but lacked external validation and relied on nuclear magnetic resonance (NMR)-based profiling, which captures aggregate lipid measures and only a limited segment of the molecular lipidome. Overall, available evidence indicates incremental predictive value of lipidomic and genomic data but highlights the absence of clinically integrated, generalisable, and implementation-ready approaches.

**Added value of this study**

This study shows that lipidomic and genomic risk components can be jointly embedded within a contemporary, guideline-endorsed clinical risk algorithm to improve risk stratification in a clinically actionable manner. By augmenting AusCVDRisk rather than replacing it, we preserved absolute risk interpretation in the target population while selectively enhancing risk resolution among individuals at intermediate clinical risk. Across multiple Australian cohorts, the combined lipidomic–genomic model substantially improved risk stratification among intermediate-risk individuals, particularly by correctly up-classifying future events, and better identified individuals with substantial subclinical plaque burden. Decision-curve analysis showed that clinical benefit was greatest when molecular testing was applied conditionally to the intermediate-risk group. The approach integrates machine-learning methods for high-dimensional lipidomic data with conventional survival modelling, maintaining interpretability and compatibility with existing care pathways.

**Implications of all the available evidence**

Taken together, the evidence suggests that conventional clinical risk scores alone are insufficient for a substantial subset of patients, that complementary molecular profiling can meaningfully refine risk estimation, and that targeted rather than population-wide deployment is likely to be most feasible and cost-effective. Conditional integration of lipidomic and genomic risk within existing clinical pathways could improve targeting of preventive therapies and help identify intermediate-risk individuals with substantial subclinical atherosclerotic burden who may benefit from further diagnostic imaging. As scalable clinical lipidomic platforms and genotyping become increasingly standardised, such molecularly augmented risk assessment represents a pragmatic next step. These findings support prospective evaluation of lipidomic-genomic-enhanced risk assessment in real-world primary care, including studies of feasibility, impact on clinical decision-making, and cardiovascular outcomes, including surrogate imaging endpoints of atherosclerotic burden.

## Introduction

Cardiovascular disease (CVD) remains the leading cause of morbidity and mortality worldwide, accounting for nearly one-third of global deaths and contributing substantially to the disease burden in Australia.^1^^,^^2^ To guide primary prevention, several population-based CVD risk scores have been developed, including the Pooled Cohort Equations for Atherosclerotic Cardiovascular Disease (PCE-ASCVD) and Predicting Risk of Cardiovascular Disease Events (PREVENT) in the United States, Systematic Coronary Risk Evaluation 2 (SCORE2) in Europe, and QRISK in the United Kingdom.^3^, ^4^, ^5^, ^6^ In Australia and New Zealand, AusCVDRisk and PREDICT-1 are widely used and have improved identification of high-risk individuals, helping to target preventive interventions where they are most likely to be beneficial.^7^, ^8^, ^9^ Yet, despite their demonstrated utility, these models have notable limitations: globally, over 60% of CVD events occur in people without known CVD who are classified as low or intermediate risk,^10^ underscoring the need for more precise and actionable risk stratification.

The intermediate-risk group comprises about 17% of those at risk but contributes 27% of all CVD events^10^ (19% and 35% when limited to individuals without previous CVD). Improving prediction accuracy in this group is pivotal to directing preventive therapies where they are most effective.^11^ Progress beyond current clinical models will likely require incorporation of biological information not captured by conventional risk factors, particularly molecular data such as lipidomics and genomics, which can serve as sensitive indicators of biological processes relevant to atherosclerotic risk. High-resolution lipidomics enables detailed characterisation of hundreds of circulating lipid species beyond traditional cholesterol measures,^12^^,^^13^ while genomic profiling captures inherited susceptibility not reflected in clinical measurements. Together, these approaches capture complementary dimensions of atherosclerotic risk–current metabolic state and lifelong genetic predisposition. Both modalities have been associated with atherosclerotic plaque burden, including non-calcified plaque detected by coronary angiography^14^, ^15^, ^16^, ^17^ In turn, lipidomic, metabolic, and polygenic risk scores have demonstrated incremental value for cardiovascular risk stratification.^18^, ^19^, ^20^, ^21^, ^22^

Despite these advances, translating deep molecular profiling, particularly lipidomics, into routine risk assessment remains challenging. Large-scale cohorts underpinning clinical risk algorithms, such as PREDICT-1 with over 400,000 primary care patients, typically lack deep molecular phenotyping. In contrast, other observational studies, whether small or large, may offer rich molecular data but often lack the population-wide representation or statistical power required to derive generalisable scores suitable for routine clinical decision support.

In the present study, we address this challenge by integrating lipidomic and genomic data into the AusCVDRisk framework using several Australian population cohorts. Each cohort included harmonised clinical measures, deep lipidomic and genomic profiling, and at least five years of follow-up for CVD outcomes. This approach augments an established, guideline-endorsed risk algorithm with complementary molecular information, enabling selective refinement of risk estimates while preserving absolute risk interpretation. As such, it provides a scalable foundation for precision prevention in primary care, particularly for patients whose risk is not fully captured by standard clinical scores.

## Methods

### Study design and cohorts

We derived lipidomic-enhance and genomic-enhanced CVD risk scores using two Australian population cohorts: the Australian Diabetes, Obesity and Lifestyle Study (AusDiab)^23^^,^^24^ and the Busselton Health Study (BHS).^25^^,^^26^ External validation analyses were conducted in three additional cohorts: the Melbourne Collaborative Cohort Study (MCCS),^27^ the Aspirin in Reducing Events in the Elderly (ASPREE),^28^^,^^29^ and the BioHEART-computed tomography (BioHEART-CT) study.^30^^,^^31^ The contributing cohorts are briefly described below, with full study protocols reported previously. Participants eligible for model development were aged 35–85 years, had no previously reported CVD, and had available lipidomic and/or genomic data (Supplementary Fig. S1). External validation applied the same eligibility criteria but was additionally restricted to participants aged 45–79 years, or 35–79 years if they had diabetes, in accordance with the Australian guideline for CVD risk assessment^8^ (Supplementary Fig. S2). The broader age range used for model development was intended to maximise statistical power. Data reporting follows the principles outlined in the TRIPOD + AI statement.

AusDiab is a prospective cohort study aimed at identifying risk factors for diabetes mellitus and cardiometabolic disease, with participants drawn from randomly selected urban and rural areas of Australia. The baseline survey was conducted between 1999 and 2000.^23^^,^^24^ The lipidomic CVD score was derived using data on 8082 AusDiab participants aged 35–85 years, without previous CVD events.

The BHS is a prospective community-based cohort comprising a series of health surveys in the City of Busselton, Western Australia (www.bpmri.org.au).^25^^,^^26^ Polygenic CVD scores were integrated into AusCVDRisk using data on 3328 BHS participants from the 1994–95 follow-up survey treated as baseline, who were 35–85 years old and without previous CVD events.

MCCS is a prospective cohort study established between 1990 and 1994 to investigate the role of diet and lifestyle in chronic diseases in the area of metropolitan Melbourne.^27^ For validation purpose of the present study, we derived a MCCS case-cohort by drawing participants from the second MCCS follow-up (2003–07), which was treated as baseline. The MCCS case-cohort was formed by randomly selecting 2200 individuals (sub-cohort) and enriching them with participants from the remainder of cohort’s second follow-up who died from ischaemic heart disease within 5-year follow-up.

ASPREE is a randomised trial aimed at identifying the effect of daily use of aspirin on disability-free survival in older adults. The incidence of CVD and major bleeding were also studied.^28^^,^^29^ Between 2010 and 2014, the trial enrolled 19,114 adults aged 70 years and over from Australia and 65 years and over from the United States who did not have CVD, dementia, or physical disability. For the present study, we derived an ASPREE case-cohort by randomly selecting 3287 Australian participants (sub-cohort) and enriching them with the remainder of enrolled participants who developed incident myocardial infarction or died from coronary heart disease within 5-year follow-up.

BioHEART-CT is a multicentre, prospective cohort study of patients with suspected coronary artery disease who were clinically referred to undergo CT coronary angiography imaging.^30^^,^^31^ Recruitment commenced in 2017 and is ongoing. For validation purpose of the present study, we used data on the first 992 recruited primary prevention participants, 45% (446) of which were asymptomatic.

### Outcomes

5-year incident CVD was defined in line with the Framingham, PREDICT-1, and AusCVDRisk algorithms^7^^,^^9^^,^^32^ as hospitalisation or death from ischaemic heart disease (including angina), cerebrovascular events (ischaemic or haemorrhagic, including transient ischaemic attacks), peripheral vascular disease, congestive heart failure; or a death from other ischaemic cardiovascular causes. Most outcomes were identified via data linkage using International Classification of Diseases, Tenth Revision, Australian Modification (ICD-10-AM) codes (Supplementary Table S1). Exceptions included: in AusDiab, nonfatal events were self-reported and subsequently adjudicated, episodes of ischaemic heart disease, coronary revascularization procedures, and cerebrovascular events; in ASPREE, the prespecified secondary endpoint (covering fatal/nonfatal myocardial infarction and stroke, including haemorrhagic stroke, coronary death, and heart failure hospitalisation) was extended using Medical Dictionary for Regulatory Activities (MedDRA)-coded hospitalisations aligned with the ICD-10-AM codes above; and in MCCS, outcomes were limited to CVD mortality. In BioHEART, where adjudicated events were not yet available, the outcome of interest was the coronary artery calcium (CAC) score, with scores of 101–400 indicating moderate and scores above 400 indicating extensive evidence of coronary artery disease.

Follow-up was from baseline to first qualifying CVD event, non-CVD death, loss of follow-up, or five years, whichever occurred first.

### Baseline clinical score (AusCVDRisk)

5-year risk was calculated using the AusCVDRisk general population algorithm (www.cvdcheck.org.au),^9^ a recalibrated version of the PREDICT-1 equation^7^ derived from a large New Zealand primary care cohort (AUS-PREDICT, over 300,000 participants). As part of the 2023 Australian Guideline for assessing and managing cardiovascular disease risk, the equation was recalibrated to the Australian population and modified for the Australian healthcare system.^8^

Required variables were age, sex, systolic blood pressure (SBP), total/high-desity lipoprotein cholesterol ratio (TC/HDL-C ratio), diabetes, atrial fibrillation, blood pressure-lowering, lipid-modifying, and antithrombotic medication use, smoking status, and Index of Relative Socio-economic Disadvantage from Australian Socio-Economic Index for Areas. Atrial fibrillation was unavailable in two cohorts, excluded by design in ASPREE, and coded as absent (zero) in all studies. Missing data in required variables were singly imputed using a k-nearest neighbours algorithm. This affected 43 values in 43 AusDiab participants, 324 in 71 BHS participants, 617 in 347 MCCS participants, 165 in 88 ASPREE participants, and 22 in 22 BioHEART participants. For each missing observation, the five nearest participants (k = 5) were identified using Gower’s distance based on all available AusCVDRisk predictors, together with body mass index and fasting blood glucose. The missing value was subsequently imputed using the median value of the corresponding variable among these neighbours.

The AusCVDRisk calculator also includes clinically determined high-risk criteria (chronic kidney disease, familial hypercholesterolaemia), and optional reclassification factors (ethnicity, kidney function, severe mental illness, family history of premature CVD, and CAC score) that may be used to refine risk strata near decision thresholds. These factors were not applied in the present study because relevant data were unavailable for most cohorts; in BioHEART the CAC score served as an outcome rather than a reclassification variable.

Baseline survivor functions and mean risk-factor values were fixed to AusCVDRisk equation values (Supplementary Table S2).

### Lipidomic profiling

Each cohort was profiled using the standardised methodology described by Huynh et al.^12^ Lipidomic profiling was previously described for AusDiab,^33^ BHS,^34^ MCCS^35^ ASPREE^36^ and BioHEART.^18^ Briefly, 10 μL of plasma (for AusDiab, MCCS, ASPREE, and BioHEART) or serum (for BHS) was spiked with an internal standard mix, after which lipid species were isolated using a single-phase butanol/methanol extraction method.^37^ Targeted lipidomic profiling of extracts was performed using liquid chromatography coupled electrospray ionisation-tandem mass spectrometry on an Agilent 1290 high-performance liquid chromatography/Agilent 6490 QQQ mass spectrometer (LC-MS/MS) system. To generate concentration data, chromatographic/mass spectrometry peak areas for each lipid species were related to the corresponding internal standard. All datasets were harmonised using lipid-specific correction factors based on National Institute of Standards and Technology (NIST) Standard Reference Material 1950 (SRM 1950) plasma samples. For each lipid, the factor was computed as the ratio of the NIST SRM 1950 concentrations (median across >1000 unique NIST 1950 samples aggregated from 24 independent studies) to the median concentration of NIST 1950 samples within each cohort. To align BHS with other cohorts, we imputed 139 missing lipid species using a validated method^38^ (Supplementary Materials).

Additional technical details and instrument parameters are available on our website (https://metabolomics.baker.edu.au/method/).

### Genotyping, imputation, and polygenic scores

Participants in the BHS, MCCS, ASPREE, and BioHEART cohorts were genotyped using genome-wide SNP arrays, with genotyping and quality control performed as previously described.^34^^,^^39^^,^^40^ Details of genotyping platforms, imputation reference panels, and post-imputation quality control thresholds are provided in Supplementary Table S3. We obtained 50 PGS for coronary artery/heart disease or stroke from the Polygenic Score Catalogue,^41^ and scored them in each cohort using the PLINK 2 software (v2.31).^42^ In the MCCS and ASPREE case-cohorts, allele-frequency estimates for score calculation were derived from the randomly selected subcohort to minimise the impact of case enrichment on the PGS distribution.

To select the final PGS for our model, individual PGS were assessed on their improvement to model fit and general predictive/discriminative performance over the baseline Cox regression model containing only AusCVDRisk or lipidomic-enriched CVD risk score (linear predictor) as an offset term. Additionally, we considered the breadth and power of each reported PGS, i.e., number of variants and individuals included in the original study.

### Development of lipidomic and polygenic CVD risk models

**Fig. 1:**
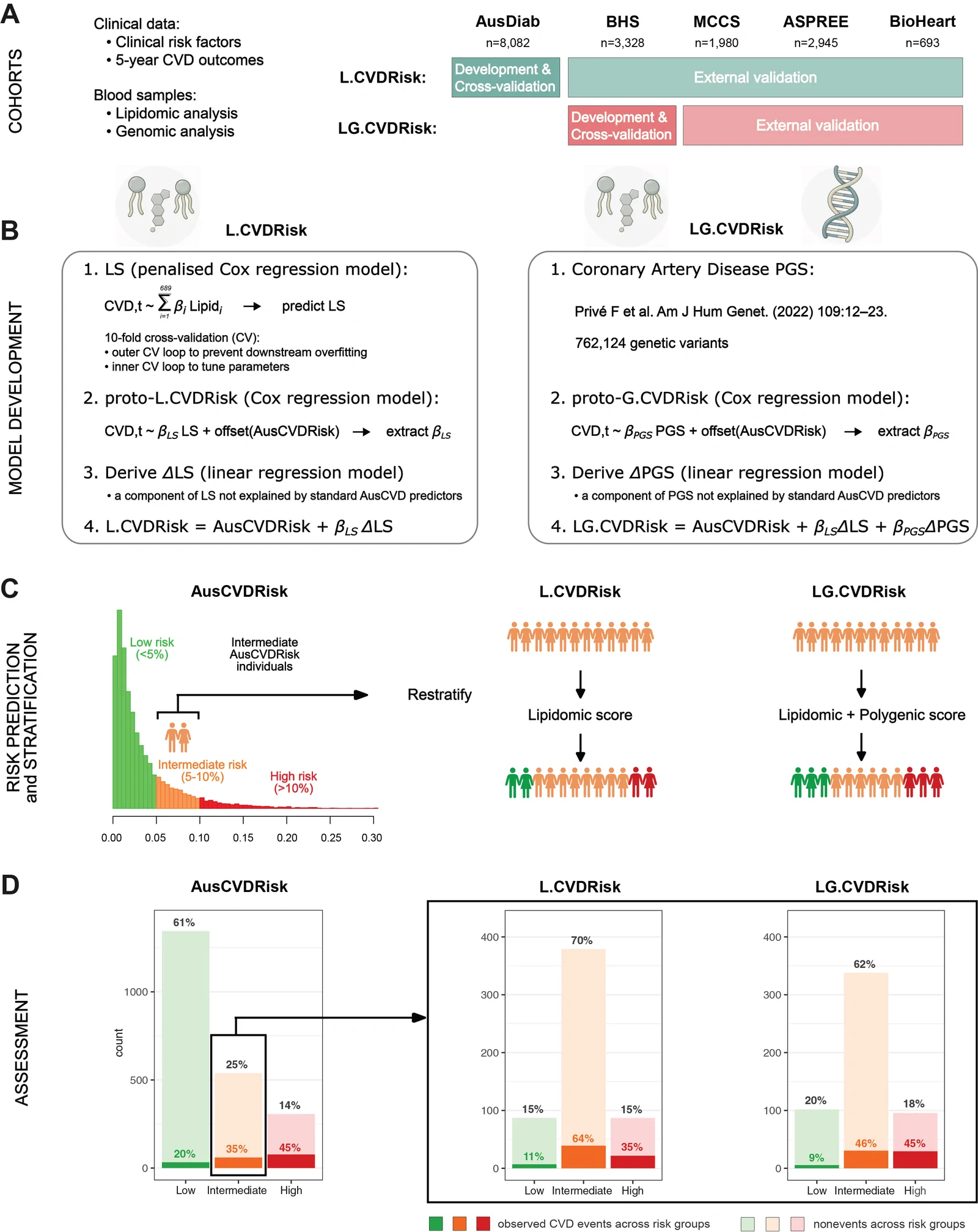
**Workflow for development and validation of lipidomic-enhanced and genomic-enhanced CVDRisk scores.** Panel A shows the cohorts used for model development and external validation. Panel B summarises model construction, illustrating integration of the lipidomic score (LS) and polygenic score (PGS) into the established AusCVDRisk framework. Panel C illustrates reclassification of individuals initially classified as intermediate risk by AusCVDRisk into low-, intermediate-, or high-risk categories by L.CVDRisk and LG.CVDRisk. Panel D shows how reclassification in validation cohorts contributes to performance assessment through correct and incorrect reclassification of CVD events and non-events. Percentages represent approximate trends across cohorts and are shown for illustration only; cohort-specific results are shown in Fig. 4. Abbreviations: CVD, cardiovascular disease; AusDiab, Australian Diabetes, Obesity and Lifestyle Study; BHS, Busselton Health Study; MCCS, Melbourne Collaborative Cohort Study; ASPREE, Aspirin in Reducing Events in the Elderly; L.CVDRisk, lipidomic-enhanced; G.CVDRisk, genomic-enhanced; LG.CVDRisk, lipidomic-enhanced and genomic-enhanced CVD risk score.

The model development workflow is summarised in Fig. 1. To improve prediction of 5-year incident CVD risk, we developed three enhanced scores: L.CVDRisk (lipidomic-enhanced), G.CVDRisk (genomic-enhanced), and LG.CVDRisk (lipidomic– genomic-enhanced), each built upon the established AusCVDRisk algorithm.

The L.CVDRisk score was constructed in a stepwise manner. First, a lipidomic score (LS) was developed using a sex-stratified elastic-net regularised Cox proportional hazards model,^43^ incorporating 689 lipid species as predictors:

(1) $$\begin{array}{c} h_{i}\left(t,s,x\right)=h_{0}\left(t,s\right)\;\exp\left(x_{i}^{T}\beta \right);\;\hat{\beta }={\mathrm{argmin}}_{\beta }\left\{\;-\;l_{Cox}\left(\beta \right)\;+\;\lambda \left(\alpha {\left\|\beta \right\|}_{1}\;+\;\left(1\;-\;\alpha \right){\left\|\beta \right\|}_{2}^{2}\right)\right\} \end{array}$$

Here $h_{i}\left(t,s,x\right)$ and $h_{0}\left(t,s\right)$ denote the estimated and baseline hazard, at time t, for an individual i of sex s; $x_{i}$ is the vector of log-transformed lipid species concentrations for this individual, β is the coefficient vector. Inference is made via penalised partial log-likelihood ($l_{Cox}\left(\beta \right)$), where λ and α control the strength and type of penalty. Extreme log-transformed lipid concentrations were winsorised at 10 median absolute deviations.

The LS was derived in a nested cross-validation procedure (10-fold outer and inner loops) to prevent information leakage between training and prediction folds in each iteration, and ultimately to prevent overfitting in the next step. Inner loops were used for the hyperparameter λ tuning, outer loops for 10 rounds of score predictions which were subsequently combined. The α parameter was fixed at 0.05, favouring a ridge-like penalty structure–coefficient shrinkage over variable selection.

The LS was then incorporated into a standard sex-stratified Cox regression model with the AusCVDRisk linear predictor included as an offset term:

(2) $$\begin{array}{c} h_{i}\left(t,s,LS\right)=h_{0}\left(t,s\right)\;\exp\left(\beta_{LS}{LS}_{i}\;+\;{\text{AusCVD}.\text{offset}}_{i}\right) \end{array}$$

This approach ensures that the AusCVDRisk coefficients are preserved and avoids overfitting to the derivation cohort (AusDiab), while still estimating the added value of lipidomics. However, while the coefficient $\beta_{LS}$ is adjusted for the AusCVDRisk offset term, the AusCVDRisk coefficients may still include residual confounding by the LS. Indeed, lipidomic profiles are strongly associated with some AusCVDRisk predictors such as total and HDL cholesterol, age, sex and diabetes, as well as with CVD outcomes. To remove such residual confounding, we derived the residual lipidomic score (ΔLS) by regressing LS on the subset of AusCVDRisk clinical variables:

(3) $$\begin{array}{c} {LS}_{i}=\beta_{0}\;+\;x_{i}^{T}\beta \;+\;\varepsilon_{i} \end{array}$$

(4) $$\begin{array}{c} \Delta {LS}_{i}={LS}_{i}-{\hat{LS}}_{i} \end{array}$$

Here x included age, sex, diabetes status, SBP, TC/HDL-C ratio, smoking status, use of blood pressure–lowering and lipid-modifying medications, interaction of age with sex, diabetes, SBP, and interaction between SBP and blood pressure–lowering medication. The rationale for residualisation is discussed in greater detail in the Methods Supplement (Supplementary Materials).

Finally, the L.CVDRisk model was specified as:

(5) $$\begin{array}{c} {h_{i}\left(t,s,\Delta LS\right)}_{L.CVDRisk}=h_{0}\left(t,s\right)\;\exp\left(\beta_{LS}{\Delta LS}_{i}\;+\;{\text{AusCVD}.\text{offset}}_{i}\right) \end{array}$$

It consists of adding the product $\beta_{LS}\Delta {LS}_{i}$ from the steps 2 and 4 to the AusCVDRisk linear predictor.

As the AusDiab cohort lacked genotyping, the genomic component of G.CVDRisk and LG.CVDRisk were derived using the BHS data, following a similar stepwise structure. For G.CVDRisk, a selected coronary artery disease PGS replaced the LS term in the model:

(2a) $$\begin{array}{c} h_{i}\left(t,s,PGS\right)=h_{0}\left(t,s\right)\;\exp\left(\beta_{PGS}{PGS}_{i}\;+\;{\text{AusCVD}.\text{offset}}_{i}\right) \end{array}$$

(3a) $$\begin{array}{c} {PGS}_{i}=\beta_{0}\;+\;x_{i}^{T}\beta \;+\;\varepsilon_{i} \end{array}$$

(4a) $$\begin{array}{c} \Delta {PGS}_{i}={PGS}_{i}\;-\;{\hat{PGS}}_{i} \end{array}$$

(5a) $$\begin{array}{c} {h_{i}\left(t,s,\Delta PGS\right)}_{G.CVDRisk}=h_{0}\left(t,s\right)\;\exp\left(\beta_{PGS}{\Delta PGS}_{i}\;+\;{\text{AusCVD}.\text{offset}}_{i}\right) \end{array}$$

The vector x here included sex, diabetes, SBP, TC/HDL-C ratio, and smoking status.

The LG.CVDRisk model was similarly constructed by adding the products $\beta_{LS}\Delta {LS}_{i}$ and $\beta_{PGS}{\Delta PGS}_{i}$ to the AusCVDRisk linear predictor. Since PGS and LS were not significantly correlated, LS was not included in the ΔPGS derivation. The final model can be expressed as:

(5b) $${{\;h}_{i}\left(t,s,\Delta LS,\Delta PGS\right)}_{\mathrm{LG}.AusCVDRisk}=h_{0}\left(t,s\right)\;\exp\left({{\text{AusCVDRisk}.\text{offset}}_{i}+\beta }_{LS}{\Delta LS}_{i}+\beta_{PGS}{\Delta PGS}_{i}\right)$$

We inspected the baseline survival functions, tested the proportional hazards assumption using scaled Schoenfeld residuals and assessed general model fit via binned Martingale residual plots.

To convert linear predictors in equations (5), (5a), (5a) into absolute risk estimates, we applied the sex-specific baseline survivor functions and calibration factors from the AusCVDRisk model (Supplementary Table S2), ensuring comparability and interpretability in Australian primary care settings.

All modelling was conducted using R software environment (v4.4.2), primarily employing the glmnet (v4.1-8) and survival (v3.8-3) packages for regularised and standard Cox regression, respectively.

### Statistical analysis and predictive performance

Model discrimination was assessed using Harrell’s concordance index (C-statistic)^44^ to evaluate global concordance between predicted risk and observed time-to-event outcomes.

Moderate calibration was examined by grouping individuals into deciles of predicted 5-year CVD risk and plotting the mean predicted risk against the corresponding Kaplan–Meier estimates of observed risk. Calibration intercept (calibration-in-the-large) and slope were estimated using Poisson regression models, as previously described.^45^

Predicted 5-year CVD risk was categorised as low (<5%), intermediate (5% to <10%), or high (≥10%), in line with the thresholds recommended by the 2023 Australian guideline.^8^ Analyses within the intermediate-risk group were pre-specified, reflecting the intended clinical application of molecular profiling in individuals whose treatment decisions remain uncertain after conventional risk assessment. Reclassification improvement over the AusCVDRisk baseline was quantified using categorical net reclassification improvement (NRI) metric for censored data,^46^ and the same thresholds.

To compare the clinical utility of different hypothetical primary prevention strategies, we used net benefit and decision-curve analysis for time-to-event outcomes.^47^^,^^48^ The strategy with the highest net benefit at the recommended risk threshold (10%) was considered as the most clinically valuable.

When validating predictive performance in case-cohort samples, we applied design weights accounting for the sampling scheme.^49^^,^^50^ Individuals selected into the sub-cohort who did not experience the event were given weights equal to the inverse of the sub-cohort sampling fraction. Event cases who happened to fall within the sub-cohort were assigned a weight of one. Event cases who were sampled from outside the sub-cohort were given weights equal to the inverse of the case-sampling fraction in the remainder of the cohort. In the MCCS study, this corresponded to weights of 5.34 for sub-cohort non-events and 2.50 for cases sampled from outside the sub-cohort; in ASPREE, the corresponding weights were 3.36 and 2.20, respectively. Where appropriate, results from individual studies were pooled by inverse-variance weighting to generate summary estimates. For internal validation, scores were predicted in the 10-fold cross validation procedure to prevent information leakage between training and prediction folds.

Sample size considerations were informed by the incremental modelling strategy adopted in this study. Because the omics-enhanced models were developed by augmenting the established AusCVDRisk linear predictor, rather than by re-estimating a prediction model de novo, the two derivation models (proto-L.CVDRisk in AusDiab and proto-G.CVDRisk in BHS) each contained only a single free predictor in addition to the fixed offset term (LS and PGS, respectively). With 190 incident CVD events in AusDiab and 166 in BHS, both derivation models substantially exceeded the traditional 10–20 events-per-variable guidance for unpenalised regression models. The derivation of the LS and calculation of the polygenic score (PGS) were not considered subjects of conventional prediction-model sample size calculations. Rather, these represented intermediate feature-extraction procedures used to summarise lipidomic and genomic information associated with future CVD risk. In addition, LS development employed penalised Cox regression with internal cross-validation to reduce overfitting in the high-dimensional setting. Methods Supplement contains further details about the calibration, NRI, net benefit, and sample size calculations (Supplementary Materials).

### Ethics

All participants provided informed consent. Each study was conducted in accordance with the Declaration of Helsinki and approved by the relevant institutional human research ethics committees: AusDiab (Alfred Human Research Ethics Committee, Melbourne, Australia; approval no.: 41/18), BHS (University of Western Australia Human Research Ethics Committee; approval no.: 608/15), MCCS (Cancer Council Victoria Human Research Ethics Committee; approval no.: 23/13), ASPREE (Alfred Human Research Ethics Committee; approval no.: 523/21), and BioHEART (Northern Sydney Local Health District Human Research Ethics Committee; approval no.: 2019/ETH08376).

### Role of the funding source

The funders of the study had no role in study design, data collection, analysis, and interpretation, writing of the report, or in decision to submit the paper for publication.

## Results

We developed the lipidomic-enhanced and genomic-enhanced risk scores using data from 11,410 adults aged 35–85 years without prior CVD who collectively accrued 55,868 person-years and 356 incident cardiovascular events over five years of follow-up. L.CVDRisk was derived in the AusDiab cohort and validated in BHS, MCCS, ASPREE, and BioHEART. The genomic component of G.CVDRisk and LG.CVDRisk was derived in the BHS cohort, and scores validated in MCCS, ASPREE, and BioHEART.

Participant flow diagrams for derivation and validation cohorts (Supplementary Figs. S1 and S2) show the numbers of participants excluded from each cohort, most commonly because of age outside the eligible range, prior CVD, unavailable information on prior or incident CVD, or missing lipidomic, genomic, or outcome data. Baseline characteristics of the derivation and validation cohorts are summarised in Table 1 and Table 2. Mean baseline age in derivation cohorts was 52.8 years (SD 11.8) in AusDiab and 55.3 years (13.4) in BHS, with corresponding 5-year CVD incidence of 190 (2.4%) of 8082 and 166 (5.0%) of 3328. Validation included 13,521 eligible individuals (aged 45–79 years; 35–79 if they had diabetes) across internal and external validation cohorts, with mean ages ranging from 57.4 years (SD 9.3) to 73.5 years (2.5) and 5-year CVD incidence from 156 (2.7%) of 5673 to 137 (6.1%) of 2230. In BioHEART, where adjudicated CVD events were unavailable, analyses were based on the detection of coronary artery disease as indicated by CAC scores.

**Table 1:** Baseline characteristics of participants in derivation cohorts.

	AusDiab			BHS		
	Overall N = 8082	Men N = 3545	Women N = 4537	Overall N = 3328	Men N = 1433	Women N = 1895
Incident CVD events	190 (2.4%)	117 (3.3%)	73 (1.6%)	166 (5.0%)	93 (6.5%)	73 (3.9%)
Mean follow-up time, years	4.92 (0.52)	4.87 (0.64)	4.95 (0.39)	4.85 (0.68)	4.8 (0.78)	4.89 (0.58)
Age, years	52.8 (11.8)	52.7 (11.7)	53 (11.9)	55.3 (13.4)	55.2 (13.1)	55.4 (13.7)
Smoking						
Current smoker	1188 (15%)	581 (16%)	607 (13%)	391 (12%)	209 (15%)	182 (9.6%)
Ex smoker	2332 (29%)	1274 (36%)	1058 (23%)	1037 (31%)	591 (41%)	446 (24%)
Never smoker	4562 (56%)	1690 (48%)	2872 (63%)	1900 (57%)	633 (44%)	1267 (67%)
SEIFA	3.28 (1.47)	3.30 (1.48)	3.27 (1.46)	3.00 (0.00)	3.00 (0.00)	3.00 (0.00)
SBP, mmHg	129.5 (18.3)	132.6 (16.9)	127.1 (19.1)	125.6 (17.9)	128 (16.1)	123.8 (18.9)
Total cholesterol, mmol/L	5.76 (1.06)	5.77 (1.04)	5.75 (1.07)	5.74 (1.08)	5.69 (1.00)	5.79 (1.13)
HDL cholesterol, mmol/L	1.44 (0.39)	1.27 (0.32)	1.57 (0.39)	1.41 (0.40)	1.21 (0.30)	1.56 (0.40)
Diabetes	507 (6.3%)	243 (6.9%)	264 (5.8%)	125 (3.8%)	63 (4.4%)	62 (3.3%)
Atrial fibrillation	–	–	–	29 (0.9%)	13 (0.9%)	16 (0.8%)
Blood pressure medication	1158 (14%)	445 (13%)	713 (16%)	634 (19%)	222 (15%)	412 (22%)
Lipid-lowering medication	544 (6.7%)	233 (6.6%)	311 (6.9%)	78 (2.3%)	33 (2.3%)	45 (2.4%)
Antithrombotic medication	–	–	–	637 (19%)	223 (16%)	414 (22%)
AusCVDRisk group						
High	534 (6.6%)	397 (11%)	137 (3.0%)	427 (13%)	271 (19%)	156 (8.2%)
Intermediate	1193 (15%)	728 (21%)	465 (10%)	561 (17%)	292 (20%)	269 (14%)
Low	6355 (79%)	2420 (68%)	3935 (87%)	2340 (70%)	870 (61%)	1470 (78%)

N (%) for categorical variables; Mean (SD) for numerical variables.

AusDiab, Australian Diabetes, Obesity and Lifestyle Study; BHS, Busselton Health Study; CVD, cardiovascular disease; SEIFA, Australian Socio-Economic Index for Areas; SBP, systolic blood pressure; HDL, high-density lipoprotein.

**Table 2:** Baseline characteristics of participants in internal and external validation cohorts.

	AusDiab N = 5673	BHS N = 2230	MCCS^a^ N = 1980	ASPREE^a^ N = 2945	BioHeart N = 693
Incident CVD events	156 (2.7%)	137 (6.1%)	130 (3.0%)	275 (4.6%)	–
Mean follow-up time, years	4.90 (0.57)	4.81 (0.75)	4.88 (0.57)	4.79 (0.78)	–
Age, years	57.4 (9.3)	60.8 (9.7)	65.4 (8.1)	73.5 (2.5)	61.9 (8.9)
Sex, women	3164 (56%)	1245 (56%)	1106 (56%)	1731 (53%)	314 (45%)
Smoking					
Current smoker	711 (13%)	223 (10%)	99 (4.9%)	92 (2.9%)	38 (5.5%)
Ex smoker	1697 (30%)	729 (33%)	668 (34%)	1291 (43%)	282 (41%)
Never smoker	3265 (58%)	1278 (57%)	1213 (62%)	1562 (54%)	373 (54%)
SEIFA	3.33 (1.45)	3.00 (0.00)	3.33 (1.40)	3.33 (1.45)	3.00 (0.00)
SBP, mmHg	132.9 (18.5)	129.1 (17.5)	136.6 (18.2)	139.3 (15.8)	132.0 (16.1)
Total cholesterol, mmol/L	5.88 (1.05)	5.94 (1.07)	5.17 (0.94)	5.24 (0.97)	4.54 (0.96)
HDL cholesterol, mmol/L	1.45 (0.4)	1.40 (0.41)	1.61 (0.44)	1.58 (0.46)	1.34 (0.41)
Diabetes	491 (8.7%)	117 (5.2%)	211 (9.9%)	297 (10%)	54 (7.8%)
Atrial fibrillation	–	23 (1.0%)	–	0 (0.0%)	76 (11%)
Blood pressure medication	1057 (19%)	543 (24%)	328 (16%)	1662 (56%)	286 (41%)
Lipid-lowering medication	513 (9.0%)	77 (3.5%)	41 (2.0%)	1138 (38%)	231 (33%)
Antithrombotic medication	–	544 (24%)	201 (10%)	186^b^ (5.4%)	152 (22%)
AusCVDRisk group					
High	421 (7.4%)	322 (14%)	254 (12%)	835 (27%)	80 (12%)
Intermediate	1169 (21%)	555 (25%)	619 (31%)	1503 (52%)	213 (31%)
Low	4083 (72%)	1353 (61%)	1107 (58%)	607 (21%)	400 (58%)

N (%) for categorical variables; Mean (SD) for numerical variables.

MCCS, Melbourne Collaborative Cohort Study; ASPREE, Aspirin in Reducing Events in the Elderly.

a Unweighted N (weighted %) for categorical variables; Weighted mean (SD) for numerical variables.

b 49.3% of participants in the ASPREE case-cohort were subsequently randomised to aspirin.

Lipidomic data were harmonised to a common panel of 689 lipid species spanning 36 classes, including the major glycerophospholipid, sphingolipid, glycerolipid, sterol, and fatty acyl classes (Supplementary Table S4). AusDiab and BHS measured 743 and 595 lipid species, respectively, with 575 overlapping; MCCS, ASPREE, and BioHEART, each contained the full harmonised panel of 689 lipid species. To align BHS with the common panel, 139 missing lipid species were imputed using models developed in the AusDiab cohort. Imputation performance was evaluated across independent cohorts and demonstrated high agreement between measured and imputed lipid concentrations, while sensitivity analyses showed that inclusion of the imputed lipid species had negligible impact on the predictive performance of the L.CVDRisk model (Supplementary Fig. S3).

From published coronary artery- and cerebrovascular-disease polygenic scores (Supplementary Table S5), we selected PGS002048^51^ for its broad genomic coverage and good incremental improvement in model fit and predictive performance over AusCVDRisk and L.CVDRisk. The score was derived in 391,124 UK Biobank participants and comprises 762,124 variants.

Fig. 1 outlines the stepwise development of L.CVDRisk and LG.CVDRisk, in which adjusted lipidomic and genomic CVD risk components were layered onto AusCVDRisk. Coefficients (log hazard ratios) for individual lipid species from the initial LS regularised Cox regression model (step 1), along with mean centring values, are shown in Supplementary Fig. S4 and Table S4. In the proto-models, prior to residualisation (step 2), hazard increased by 41% per standard-deviation increase in LS (HR 1.41, 95% CI 1.22–1.63) and by 32% per standard-deviation increase in PGS (HR 1.32, 1.13–1.54). Coefficients for AusCVDRisk predictors used in the linear modelling and residualisation of the LS and PGS (step 3) are shown in Supplementary Tables S6 and S7, while Supplementary Fig. S5 summarises the distribution of observed and predicted LS across cohorts. Diagnostic checks supported the proportional-hazards assumption and adequate model fit (Supplementary Fig. S6).

**Fig. 2:**
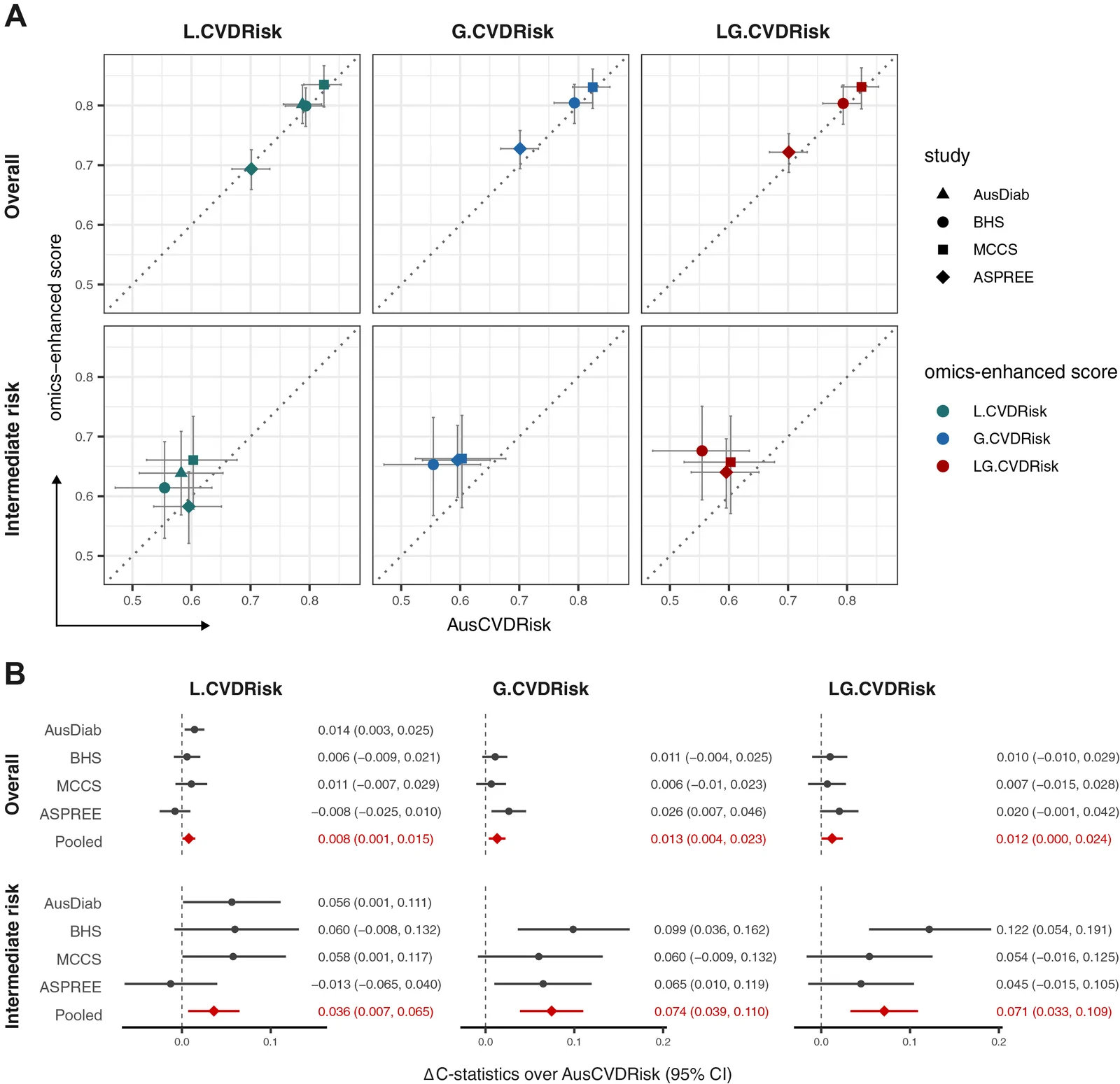
**Predictive discrimination with AusCVDRsik, L.CVDRisk, G.CVDRisk, and LG.CVDRisk.** (A) C-statistics across individual cohorts. In MCCS and ASPREE, C-statistics were weighted to account for case-cohort designs. (B) Improvements in C-statistics over AusCVDRisk (ΔC) in individual cohorts as well as pooled estimates. 95% confidence intervals (CI) were based on 10,000 bootstrap samples.

Across cohorts, lipidomic-enhanced or genomic-enhanced scores generally showed higher C-statistic point estimates than AusCVDRisk, except for L.CVDRisk in ASPREE (Fig. 2A and Supplementary Table S8). When validation assessments were pooled, discrimination gains over AusCVDRisk were modest overall (LG.CVDRisk ΔC 0.012, 95% CI 0.000 to 0.024) but more pronounced among individuals at intermediate baseline risk, AusCVDRisk 5% to <10%, (LG.CVDRisk ΔC 0.071, 0.033–0.109) (Fig. 2B). In individual cohorts, changes in discrimination were generally small and often not statistically significant, which was not unexpected given the strong baseline performance of AusCVDRisk and the shared variance between conventional risk factors and molecular scores (Supplementary Tables S6 and S7). Discrimination gains were significant among BHS participants at intermediate risk (ΔC 0.122, 0.054–0.191).

Moderate calibration demonstrated generally good agreement between predicted and observed risk across cohorts, with some departure from unity evident in case-cohorts, MCCS and ASPREE (Supplementary Fig. S7). Calibration-in-the-large showed no significant departure from zero, except in AusDiab, where observed event rates were lower than those in the AusCVDRisk derivation cohort (AUS-PREDICT). Calibration slopes were close to or above 1.0, indicating no evidence of overfitting to the derivation cohorts, as expected given that the models were developed by augmenting rather than refitting the underlying clinical model. In MCCS and ASPREE, calibration slopes were estimated in the original case-cohort analytic sample and should therefore be interpreted alongside the weighted calibration curves, which provide a more informative assessment of moderate calibration in these studies.

Differences in calibration between cohorts likely reflect cohort-specific features, including healthy participant bias in AusDiab and ASPREE, the availability of fatal events only in MCCS, and partly community-recruited composition of the BHS cohort. Some degree of miscalibration was anticipated, as all estimates were anchored to absolute 5-year risk in the contemporary Australian population by using AusCVDRisk baseline survival and recalibration factors. Although cohort-specific recalibrations could improve alignment between predicted and locally observed risk, this would also compromise the absolute risk interpretation of scores required for consistent application of 5% and 10% clinical decision thresholds.

The primary intended application of the lipidomic-enhanced and genomic-enhanced scores was improved risk stratification among individuals at intermediate baseline risk (AusCVDRisk 5% to <10%). Focusing on this group is clinically pragmatic, as these patients are most likely to benefit from or be prioritised for more expensive molecular profiling once AusCVDRisk has been applied. When validation assessments were pooled, LG.CVDRisk yielded substantial net improvements in classification, with a categorical NRI of 0.080 (0.031–0.129) overall and 0.305 (0.212–0.397) in the intermediate-risk group (Table 3). Event-specific NRI was 0.138 (0.091–0.185) overall and 0.382 (0.293–0.470) among intermediate-risk individuals. Along with the modestly negative non-event NRI, −0,069 (−0.081, −0.056) overall and −0.068 (−0.093, −0.042), this pattern indicated a substantial gain in sensitivity for future CVD events at the expense of only a small reduction in specificity.

**Table 3:** Net reclassification improvement (NRI) including overall NRI, event NRI (NRI+), and non-event NRI (NRI−).

Data view	Cohort	Statistic	L.CVDRisk	G.CVDRisk	LG.CVDRisk
Overall	Pooled estimates	NRI	0.072 (0.033, 0.111)	0.044 (0.000, 0.089)	0.080 (0.031, 0.129)
		NRI+	0.103 (0.065, 0.141)	0.064 (0.022, 0.107)	0.138 (0.091, 0.185)
		NRI−	−0.030 (−0.036, −0.023)	−0.016 (−0.027, −0.006)	−0.069 (−0.081, −0.056)
	AusDiab	NRI	0.153 (0.079, 0.232)	–	–
		NRI+	0.167 (0.092, 0.245)	–	–
		NRI−	−0.013 (−0.022, −0.005)	–	–
	BHS	NRI	0.059 (−0.024, 0.143)	0.048 (−0.034, 0.130)	0.065 (−0.032, 0.162)
		NRI+	0.110 (0.028, 0.192)	0.044 (−0.037, 0.124)	0.110 (0.014, 0.206)
		NRI−	−0.050 (−0.066, −0.035)	0.005 (−0.012, 0.021)	−0.045 (−0.064, −0.026)
	MCCS^a^	NRI	0.090 (−0.004, 0.187)	0.010 (−0.075, 0.093)	0.085 (−0.013, 0.185)
		NRI+	0.214 (0.122, 0.307)	0.045 (−0.037, 0.125)	0.239 (0.144, 0.338)
		NRI−	−0.124 (−0.144, −0.103)	−0.035 (−0.054, −0.016)	−0.154 (−0.178, −0.131)
	ASPREE^a^	NRI	0.013 (−0.052, 0.078)	0.063 (−0.003, 0.131)	0.086 (0.017, 0.155)
		NRI+	0.010 (−0.052, 0.071)	0.089 (0.026, 0.155)	0.105 (0.039, 0.171)
		NRI−	0.003 (−0.017, 0.023)	−0.026 (−0.046, −0.006)	−0.019 (−0.042, 0.004)
Intermediate risk group	Pooled estimates	NRI	0.264 (0.194, 0.334)	0.231 (0.147, 0.315)	0.305 (0.212, 0.397)
		NRI+	0.286 (0.219, 0.354)	0.228 (0.147, 0.308)	0.382 (0.293, 0.47)
		NRI−	−0.014 (−0.032, 0.004)	−0.006 (−0.027, 0.016)	−0.068 (−0.093, −0.042)
	AusDiab	NRI	0.369 (0.229, 0.511)	–	–
		NRI+	0.313 (0.176, 0.450)	–	–
		NRI−	0.056 (0.025, 0.089)	–	–
	BHS	NRI	0.235 (0.083, 0.397)	0.250 (0.072, 0.428)	0.391 (0.194, 0.576)
		NRI+	0.261 (0.113, 0.414)	0.174 (0.001, 0.342)	0.391 (0.203, 0.567)
		NRI−	−0.026 (−0.072, 0.021)	0.076 (0.028, 0.125)	0.000 (−0.054, 0.055)
	MCCS^a^	NRI	0.215 (0.028, 0.386)	0.160 (0.008, 0.315)	0.262 (0.083, 0.428)
		NRI+	0.377 (0.198, 0.543)	0.138 (−0.010, 0.284)	0.451 (0.281, 0.608)
		NRI−	−0.163 (−0.212, −0.114)	0.022 (−0.023, 0.067)	−0.189 (−0.243, −0.133)
	ASPREE^a^	NRI	0.234 (0.126, 0.345)	0.267 (0.143, 0.386)	0.287 (0.153, 0.420)
		NRI+	0.249 (0.145, 0.356)	0.311 (0.192, 0.427)	0.334 (0.204, 0.462)
		NRI−	−0.015 (−0.043, 0.014)	−0.044 (−0.072, −0.016)	−0.046 (−0.081, −0.012)

NRI 95% confidence intervals were based on 10,000 bootstrap samples.

a NRI were weighted to account for case-cohort design of these studies.

**Fig. 3:**
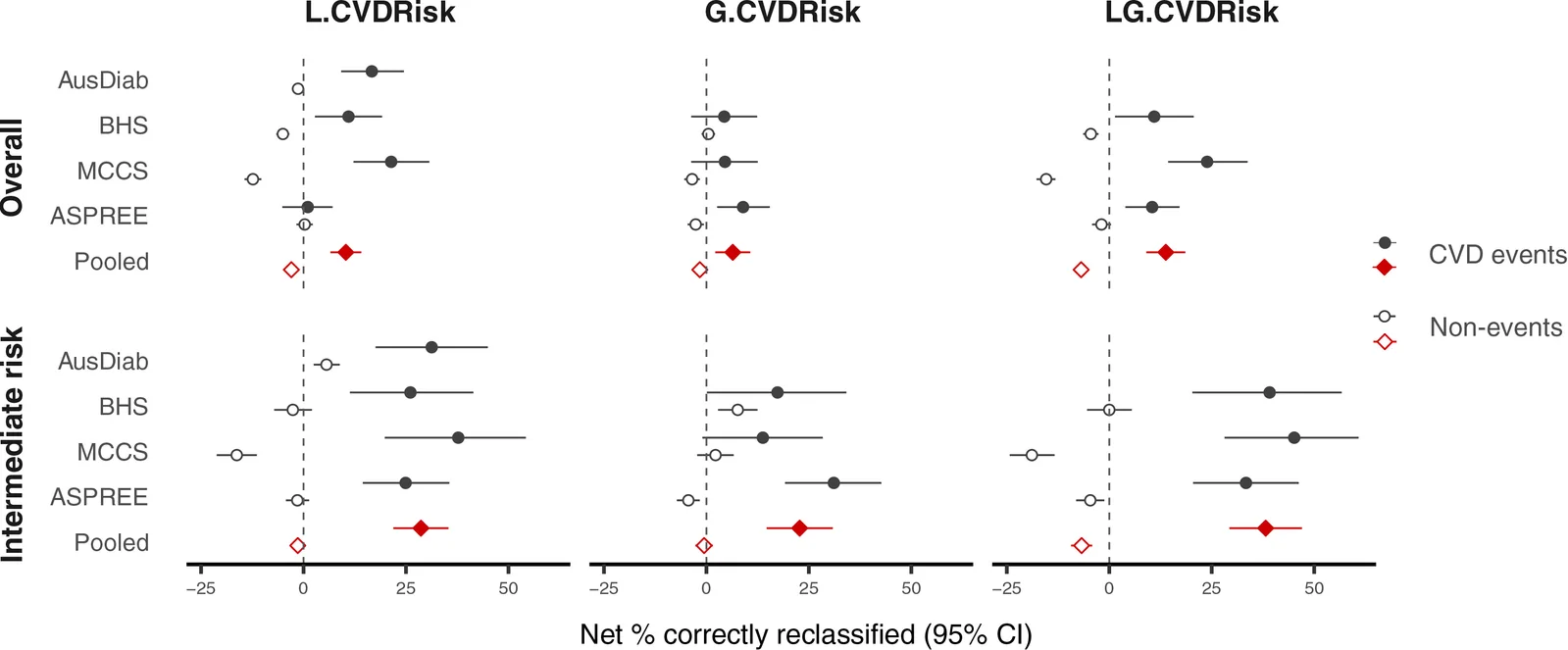
**Net reclassification improvement (NRI) with L.CVDRisk, G.CVDRisk, and LG.CVDRisk relative to AusCVDRsik.** Net % of CVD events correctly reclassified up and non-events correctly reclassified down in individual cohorts as well as their pooled estimates. In MCCS and ASPREE, statistics were weighted to account for case-cohort designs. 95% CI were based on 10,000 bootstrap samples.

**Fig. 4:**
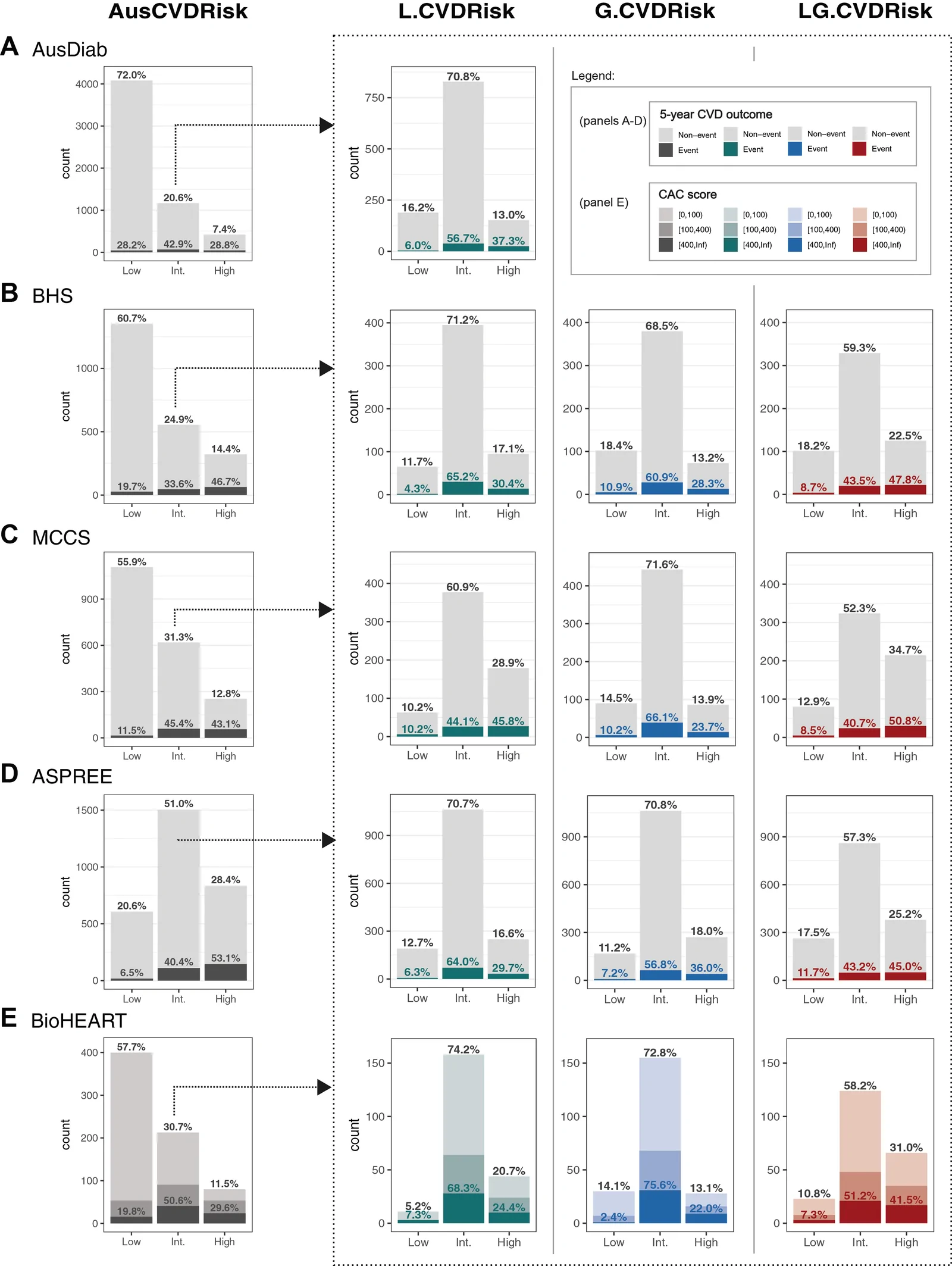
**Reclassification of intermediate-risk individuals (AusCVDRisk 5 to <10%).** Distribution of participants across low-, intermediate-, and high-risk CVD categories in five cohorts (A–E: AusDiab, BHS, MCCS, ASPREE, and BioHEART). Initial AusCVDRisk stratification was performed in the full validation cohorts (ages 45–79, or 35–79 for individuals with diabetes); intermediate-risk participants were then reclassified using L.CVDRisk, G.CVDRisk, or LG.CVDRisk. Percentages of participants in each risk category are shown at the top (black text), and percentages of total CVD events (A–D) or high-CAC cases (E) in each risk category are shown at the bottom (coloured text). Abbreviations: CAC, coronary artery calcium.

Across individual cohorts, LG.CVDRisk also showed significantly positive NRI values in the intermediate-risk group, ranging from 0.262 to 0.391, largely driven by correct upward reclassification of future events (absolute event NRI > absolute non-event NRI), with the contribution of event and non-event net reclassification shown in Fig. 3. Fig. 4A–D Further illustrates that 30–46% of events in the intermediate-risk group were reclassified as high risk by L.CVDRisk and 45–51% by LG.CVDRisk (corresponding to 33 of 111 and 27 of 59 events for L.CVDRisk, and 50 of 111 and 30 of 59 events for LG.CVDRisk in ASPREE and MCCS, respectively). In BioHEART, LG.CVDRisk reclassified 41%, or 17 of the 41 intermediate-risk patients with extensive atherosclerotic coronary artery disease burden (CAC > 400) into the high-risk group (Fig. 4E). Overall risk stratification across full validation cohorts and models is shown in Supplementary Fig. S8, corresponding to the cohort-specific overall NRI estimates in Table 3. Supplementary Tables S9 and S10 provide the underlying counts of events and non-events within risk categories and reclassification cells.

**Fig. 5:**
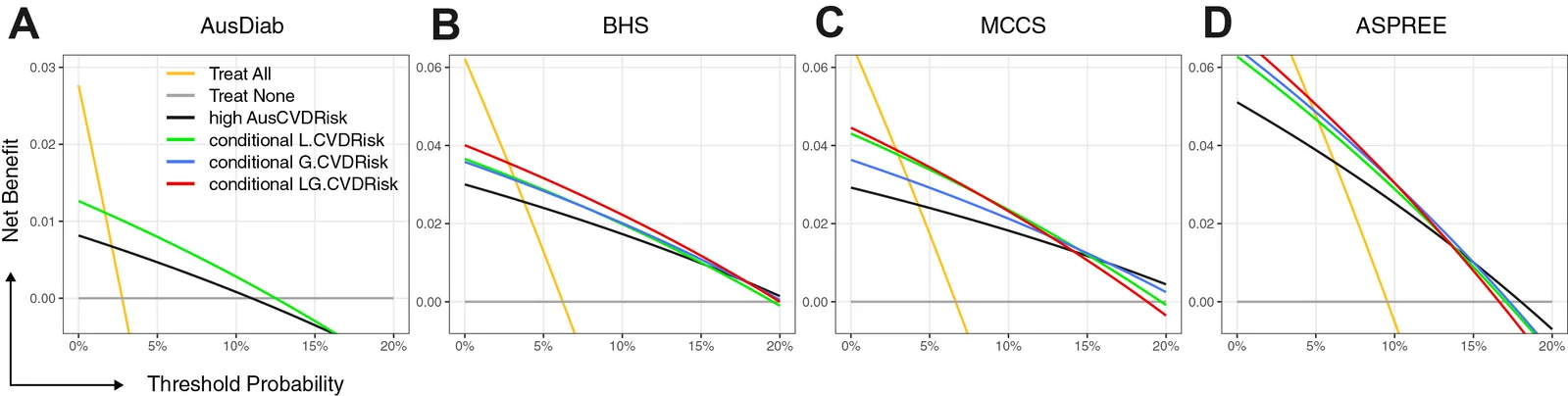
**Decision-curve analysis of current practice and conditional molecular testing strategies.** Net benefit curves compare current practice (treating individuals with AusCVDRisk ≥ 10%) with a two-step approach in which intermediate-risk individuals (AusCVDRisk 5 to <10%) undergo additional lipidomic and/or genomic risk assessment and are treated if reclassified as high risk by L.CVDRisk, G.CVDRisk, or LG.CVDRisk. Results are shown for four cohorts (A–D: AusDiab, BHS, MCCS, and ASPREE).

Decision-curve analysis compared two primary prevention strategies arising from the reclassification results: (1) current practice—treat all patients with AusCVDRisk ≥ 10% (blood pressure-lowering or lipid-modifying medications); and (2) conditional testing—in addition to current practice, apply LS/PGS profiling to patients with AusCVDRisk 5–<10% and treat those reclassified to ≥10% risk. At the recommended 10% threshold, the conditional LG.CVDRisk (as well as L.CVDRisk and G.CVDRisk) approach realised greater net benefit than AusCVDRisk alone across all cohorts (Fig. 5).

We also performed exploratory analyses comparing AusCVDRisk, L.CVDRisk, G.CVDRisk, and LG.CVDRisk as stand-alone primary CVD risk assessment tools (Supplementary Fig. S9). Although omics-enhanced scores again showed higher net benefit at the 10% risk threshold, this scenario is less clinically relevant since population-wide lipidomic, or genomic testing is not currently envisaged.

**Fig. 6:**
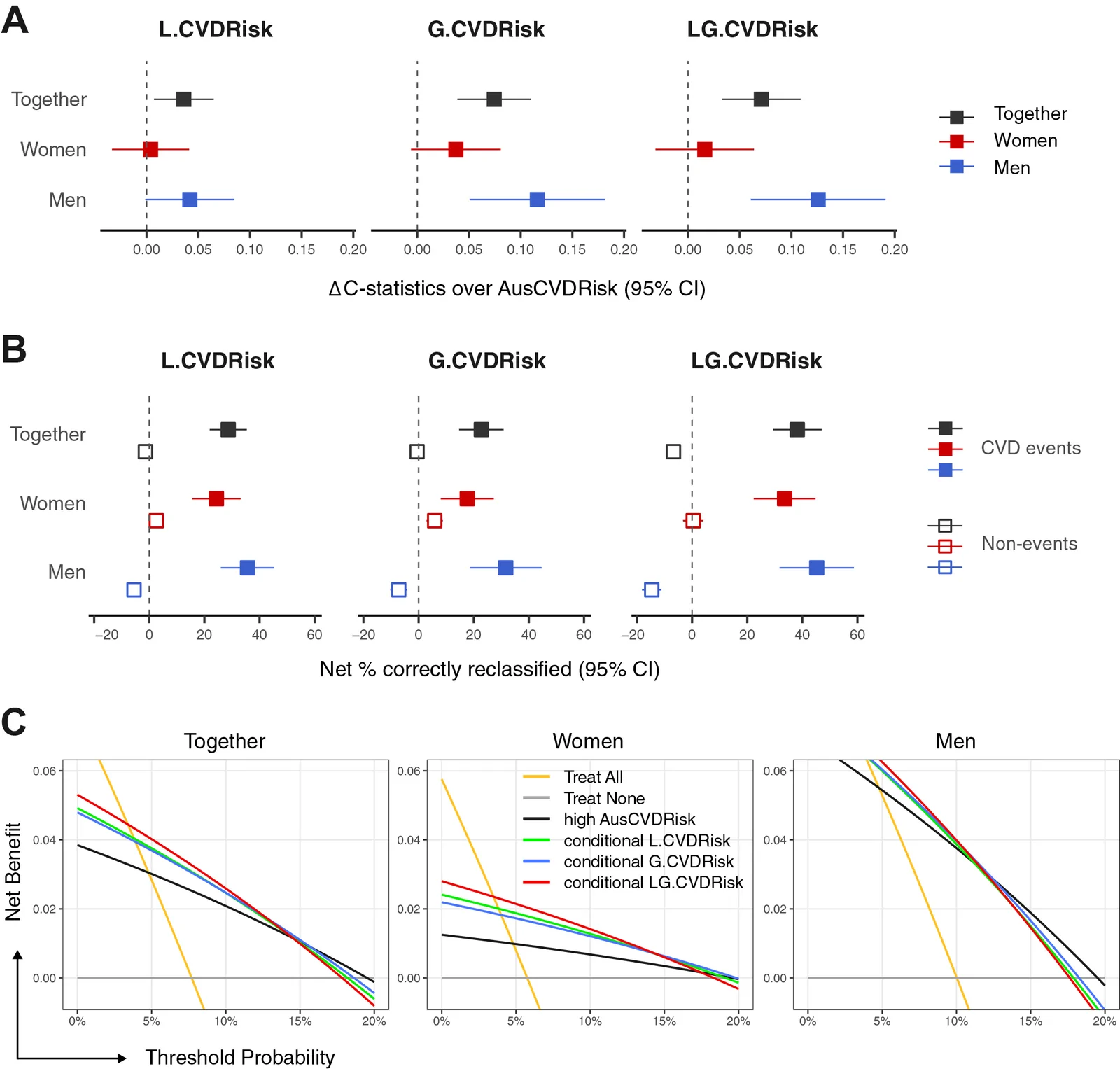
**Sex-segregated analysis of predictive performance in intermediate-risk individuals, pooled across cohorts.** (A) Improvement in discrimination (ΔC statistic) for L.CVDRisk, G.CVDRisk, and LG.CVDRisk relative to AusCVDRisk. (B) Net reclassification improvement (NRI) relative to AusCVDRisk. (C) Decision-curve analysis comparing current practice with conditional molecular testing and treatment strategies.

Sex-segregated analyses within individual cohorts were generally imprecise because of limited event numbers; however, pooling across validation cohorts revealed a consistent pattern of larger performance gains in men than women (Fig. 6). Improvements in discrimination were significant in men but not women within the intermediate-risk group: LG.CVDRisk increased C-statistic by 0.126 (95% CI 0.061–0.191) in men compared with 0.016 (−0.031 to 0.064) in women (Supplementary Table S11). Reclassification improvements remained significant in both sexes, although they were more pronounced in men. Among intermediate-risk individuals, LG.CVDRisk achieved an NRI of 0.313 (0.174–0.453) in men and 0.295 (0.177–0.414) in women, driven primarily by improved identification of future CVD events (Supplementary Table S12).

Decision-curve analysis demonstrated positive net benefit for conditional molecular testing strategies in both sexes (Fig. 6C). Absolute net benefit was higher in men, whereas the incremental gain over AusCVDRisk was proportionally larger in women. For example, conditional LG.CVDRisk testing increased net benefit from 0.007 to 0.014 in women and from 0.038 to 0.040 in men, corresponding to incremental gains of 0.007 and 0.002, respectively. Additional details on discrimination, calibration, and reclassification performance are provided in Supplementary Tables S11–13.

**Fig. 7:**
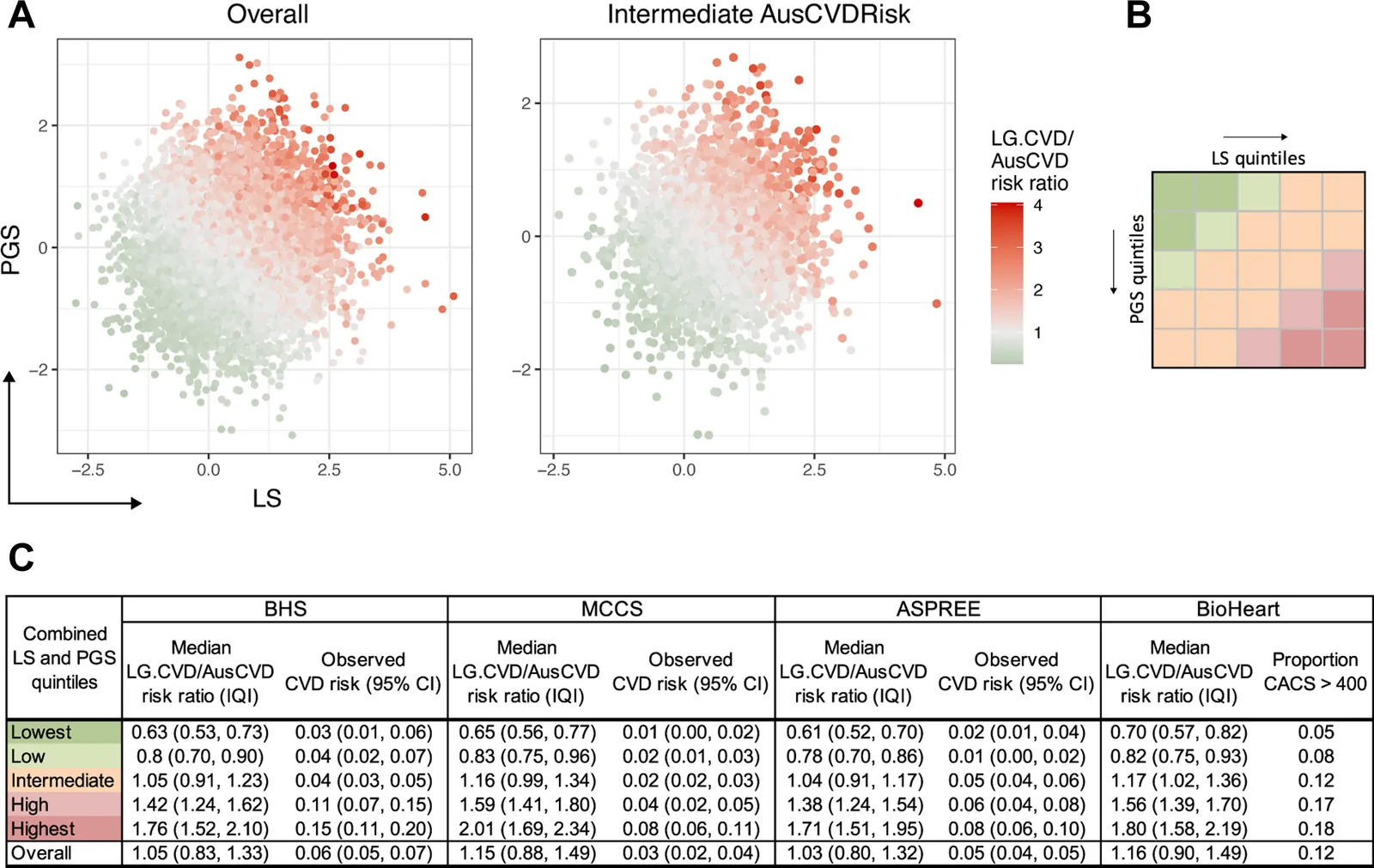
**Independent and joint contribution of lipidomic and polygenic scores when integrated into AusCVDRisk.** (A) Continuous relationship between the lipidomic score (LS), polygenic score (PGS), and the LG.CVDRisk/AusCVDRisk ratio, pooled across validation cohorts (BHS, MCCS, ASPREE and BioHEART) or restricted to intermediate-risk individuals in those cohorts, as indicated. (B) Schematic showing the combinations of LS and PGS quintiles. (C) Median predicted LG.CVDRisk/AusCVDRisk ratio and observed 5-year CVD risk (Kaplan–Meier estimates) across LS-PGS quintile combinations in individual validation cohorts. Abbreviations: IQI, interquartile interval.

The independent and joint contributions of LS and PGS to AusCVDRisk-defined risk were assessed by analysing their effect on the LG.CVDRisk/AusCVDRisk ratio. Fig. 7A explores the continuous relationship between LS, PGS, and this ratio: concordantly high LS and PGS values produced the largest upward shifts in the risk ratio, whereas opposing extremes offset each other, leaving the ratio near 1.0. The scatterplot pattern also indicates statistical independence of the two omics signals. Fig. 7B and C summarise these relationships across LS-PGS quintile combinations, showing median predicted risk ratio and observed CVD risk in combinations of lower, intermediate, and higher quintile pairs. Across validation cohorts, the highest LS and PGS quintile pairs generated the greatest median risk ratios (1.71–2.01), and observed 5-year event rates (8–15%), whereas the lowest quintile pairs were associated with risk ratios below 0.7 and event rates under 3%.

## Discussion

In this multi-cohort investigation, we showed that augmenting the AusCVDRisk equation with independent lipidomic and polygenic components provides more accurate clinical risk stratification at intermediate baseline risk. To our knowledge, this is the first study to integrate both deep-coverage lipidomics and a genome-wide polygenic score on top of a contemporary, recalibrated clinical algorithm (AusCVDRisk) in an Australian context. The combined model, LG.CVDRisk, improved risk stratification among individuals at intermediate baseline risk, with net CVD event and net non-event reclassification of 38% (95% CI 29–47%) and −6.8% (−9.3 to −4.2%), respectively. Taken together, these findings suggest substantially improved identification of future CVD events, offset by only a modest increase in false-positive classifications. Across individual studies, up to half of intermediate-risk events were reclassified to high risk. Gains in discrimination were modest (ΔC 0.071; 95% CI 0.033–0.109 within the intermediate-risk group), as anticipated given the strong baseline performance of AusCVDRisk and the fact that the enhanced models were designed to preserve the AusCVDRisk framework rather than re-estimate all clinical predictor effects. The decision-curve analysis demonstrated a clear clinical advantage for a conditional-testing strategy in which lipidomic and/or genomic profiling is applied only to patients with AusCVDRisk 5 to <10% before treatment decisions are finalised.

Our findings build on a growing body of work showing that molecular profiles can enhance CVD risk prediction beyond established clinical scores. Early efforts using nuclear magnetic resonance (NMR) metabolomics in the Atherosclerosis Risk in Communities (ARIC) cohort^52^ and more recently in the UK Biobank^53^ reported improved classification when panels of metabolites were added to the ASCVD-PCE and SCORE2, respectively. More recently, Ritchie et al. combined broad NMR-derived metabolic profiles with polygenic cardiovascular scores on top of SCORE2 in ∼187,000 UK Biobank participants, reporting ΔC 0.02 and NRI 12% for major CVD events.^22^ Their study confirms the complementary value of metabolomics and genomics, but differs from ours in two key respects: (i) the metabolic coverage was limited to NMR-detectable molecules, which largely represent lipoprotein compositions and a smaller number of polar metabolites, whereas our LC-MS/MS platform quantifies 689 lipid species across 36 classes; and (ii) the analyses were performed in a single cohort rather than across multiple Australian populations with external validation. Polygenic models alone have also shown reclassification gains when layered onto ASCVD-PCE, QRISK2 and SCORE2,^19^^,^^20^ underscoring the independent contribution of inherited risk. The methodology of our study aligns with recent kidney-function add-ons to SCORE2 and PCE-ASCVD,^54^, ^55^, ^56^ but leverages two orthogonal biological domains: circulating lipids reflecting short- to mid-term metabolic perturbation influenced by diet and metabolic state, and genomic variation representing lifetime susceptibility to inflammatory, pro-atherogenic, and thrombotic pathways. Their near-complete independence (Fig. 7, and previously reported^57^) underpins the greater improvement in risk stratification when both domains were combined.

Global discrimination metrics such as the C-statistic are relatively insensitive when new predictors correlate with strong baseline factors; nonetheless, even modest increases in discrimination may translate into meaningful clinical benefit when treatment decisions are threshold-based.^58^ The categorical NRI and decision-curve analyses therefore provide a more tangible assessment of clinical impact. At the recommended 10% 5-year risk threshold, the conditional LG.CVDRisk testing strategy delivered the highest net benefit across cohorts, implicitly accepting the 1:9 trade-off between true positives (high-risk reclassified future events) and false positives (high-risk reclassified non-events) inherent in that threshold.^48^

The intended clinical application of LG.CVDRisk is targeted upward reclassification of intermediate-risk patients (AusCVDRisk 5% to <10%) whose true risk is underestimated by standard clinical factors. In everyday practice intermediate-risk patients are already advised to adopt lifestyle changes,^8^ so a molecular score that downgrades someone to ‘low risk’ has little practical consequence—healthy-behaviour advice would not be withdrawn. In contrast, an upward shift to higher risk directly influences management: Australian guidelines allow initiation of lipid-modifying and/or blood-pressure–lowering therapy in intermediate-risk patients at clinician discretion.^8^ In this context, LG.CVDRisk serves as an additional molecular risk layer to support treatment decisions, with the explicit aim of identifying the high-risk fraction missed by conventional assessment.

Sex-segregated analyses suggested smaller performance gains in women than in men, although reclassification improvements remained evident in both sexes. Cardiovascular risk assessment in women presents recognised challenges, including lower event rates and the incomplete capture of female-specific risk factors by conventional prediction models.^59^ Interestingly, while absolute net benefit was higher in men, the proportional gain over AusCVDRisk achieved by conditional molecular testing was larger in women. This likely reflects differences in baseline risk and event rates between sexes, as net benefit is inherently dependent on the underlying prevalence of events.

Our findings in the BioHEART-CT cohort referred for coronary CT reinforce this point. Here, LG.CVDRisk reclassified more patients with extensive atherosclerotic coronary artery disease burden (CAC > 400) into the high-risk category compared with AusCVDRisk, correctly up-classifying 41% (17/41) of intermediate-risk patients with extensive coronary artery disease burden. These results support conditional use of omics-enhanced testing to identify individuals with significant subclinical disease who may benefit from further diagnostic imaging. Such an approach aligns with the growing emphasis on developing clinical pathways and diagnostic tools to detect and treat coronary artery disease before symptoms emerge.^60^^,^^61^

Economic modelling (Morton JI, unpublished) suggests that the incremental cost of high-throughput lipidomics plus array-based genotyping is justified when testing is restricted to the intermediate-risk population. This aligns with the roll-out of a scalable clinical lipidomic platform currently in translational development.^13^^,^^62^ Together, our results support a pragmatic pathway: (i) universal AusCVDRisk assessment in primary care; (ii) streamlined lipidomics and genotyping for the subset of patients in the 5–<10% risk band; and (iii) targeted pharmacotherapy for those (re)classified to ≥10% risk.

Strengths of this study include the large sample size (>13,000 participants), use of multiple cohorts spanning different risk profiles, rigorous, nested cross-validation, and preservation of the AusCVDRisk baseline hazard, thus maintaining calibration and absolute-risk interpretation in the target population. Several limitations should also be acknowledged. First, cohorts varied in composition and data availability. AusDiab and BHS were recruited two decades ago; genomic data were unavailable in AusDiab, precluding joint lipidomic–genomic model development in a single cohort; BHS included participants recruited through community clinics; ASPREE was restricted to older adults aged 70–79 years; and BioHEART comprised patients referred for imaging. Second, only fatal events were available in MCCS, reducing discrimination and non-event reclassification performance in that cohort and, consequently, to the pooled result. Third, atrial fibrillation and several optional AusCVDRisk reclassification factors were unavailable in most cohorts, potentially underestimating baseline clinical risk in a small proportion of participants. Finally, participants were predominantly of European ancestry, and no cohort adequatly represented Indigenous Australians, who experience a higher burden of CVD. Further evaluation and recalibration in more diverse populations, including First Nations communities, will be essential before broader implementation.

Together, these findings support further evaluation of omics-enhanced risk assessment in real-world primary care settings. Future studies will need to examine feasibility of point-of-care integration, including streamlined sample collection, centralised LC-MS/MS and genotyping, and secure reporting within existing pathology workflows. Complementary health-economic analyses will be required to assess cost-effectiveness and inform wider implementation.

In conclusion, integrating plasma lipidomic and polygenic risk with AusCVDRisk improves cardiovascular risk stratification, mainly by up-classifying a sizeable fraction of intermediate-risk individuals who go on to experience cardiovascular events. Conditional deployment of the LG.CVDRisk maximises clinical utility and aligns with existing cardiovascular risk assessment pathways in general practice. These findings support further evaluation of molecular risk profiling as a pragmatic precision-prevention strategy for individuals whose treatment decisions remain uncertain after conventional risk assessment.

## Contributors

PJM supervised and coordinated the study. PJP, CG, and AD led the study design. AD performed data analysis, visualisation, interpretation, and wrote the manuscript. JW, TW, KH, CY, AS, MJC, MI provided input into the study design or statistical analyses. KH, CG and TGM developed LC-MS/MS methods and provided support for the LC-MS/MS analysis. MC, NM, TD, ANF, MBM, HBB and TGM performed the acquisition, processing, and curation of lipidomic data for the cohorts. AB was data manager. JES and DJM were key investigators for the AusDiab cohort. GFW, JH, JH, JB, JB, and EKM were key investigators for the BHS cohort. MCS, RLM, and AMH were key investigators for the MCCS cohort. JJM, PL, CY, RW were key investigators for the ASPREE study. GAF, SMG, STV, CKC, MPG, JYHY were key investigators for the BioHEART-CT study. JIM and PS provided advice regarding health economic analysis. MW, LK, and EP were key contributors from the AusCVDRisk study group. All authors contributed to the critical revision and editing and approved the final version of the manuscript. AD and CG directly accessed and verified the underlying data. PJP and CG were responsible for the final decision to submit.

## Data sharing statement

All aggregate and derived data supporting the findings are provided within the manuscript and its Supplementary materials. Due to consent restrictions, individual-level participant data cannot be made publicly available. De-identified data may be shared with qualified investigators for analyses that do not conflict with ongoing projects upon request to the corresponding author and completion of a data-sharing agreement. Aggregated data supporting the findings and analytic code are available from the corresponding author upon reasonable request.

## Declaration of interests

CG, KH, HBB, JW, TW, AD, TGM and PJM are inventors on the pending patent No. PCT/AU2024/050249, “METHODS OF ASSESSING METABOLIC HEALTH”. JES received consulting fees from GSK, Astra Zeneca, Sanofi, Novo Nordisk, Eli Lilly, Roche, and Abbot; payments for lectures and program committee from Astra Zeneca, and for lectures from Boehringer Ingelheim, Novo Nordisk, Roche, Zuellig Pharmaceutical, and Eli Lilly. GFW receives grants or contracts from Amgen and Arrowhead for clinical trials via Linear, and from Novartis for Clinical trials via Royal Perth Hospital. GFW declares receiving consulting fees from Amgen, CSL Sequirus, Novartis, Novo Nordisk, and Arrowhead; as well for lectures and attending meetings from Amgen, CSL Sequirus, Novartis, Novo Nordisk, Arrowhead, and MSD. CKC is on the Board of the National Heart Foundation Australia, and Western Sydney Local Health District. STV was supported by a Postdoctoral Fellowship No. 106742-2022 from the National Heart Foundation of Australia. STV received support from Amgen for attending Amgen Cardiometabolic Academy in Melbourne, Australia, and honorarium for lecture. STV received support from Novartis Pharmaceuticals Australia for attending Novartis Cardiology Congress in Melbourne, Australia, and honorarium for educational presentation. GAF was supported by Investigator grant from the National Health and Medical Research Council. GAF received grants or contracts from NSW Health (NSW Cardiovascular Research Capacity Program, Senior Researcher Grant), Abbott Diagnostic, North Foundation, Australian Cardiovascular Alliance, Sanofi, Janssen Pharmaceuticals, Roche Diagnostics International Ltd, and Novartis Foundation. GAF received honoraria for presentations from CSL, CPC Clinical Research, Sanofi, GSK, Vascentis, Boehringer-Ingelheim, Heart Foundation, Abbott, and National Heart Centre Singapore; and support for attending meetings from Novo Nordisk, Amgen, and World Heart Foundation. GAF is inventor of the following patents issued to the University of Sydney and Northern Sydney Local Health District: “Fluorinated adamantyl P2X7 receptor antagonists and uses thereof” (Application No. PCT/AU2024/050799; PCT application filed July 2023); “Biomarkers of Oxidative Stress”, (Application No. 14/069,913; US Patent 9,638,699 B2 awarded 2 May 2017); “Adamantyl P2X7 receptor antagonists and their use in the treatment of cardiovascular diseases” (Application No. PCT/AU2024/050798; PCT application filed July 2023); “Methods for treatment and prevention of vascular disease” (Application No. PCT/AU2015/000548). G.A.F serves as CMO for the non-profit, CAD Frontiers, with industry partners including, Novartis, Amgen, AstraZeneca, Siemens Healthineers, ELUCID, Foresite Labs LLC, HeartFlow, Canon, Cleerly, Caristo, Genetech, Artyra, Bitterroot Bio, New Amsterdam and Allelica. G.A.F serves as Board Director for the Australian Cardiovascular Alliance (past President); as Executive Committee Member for CPC Clinical Research; as Founding Director and CMO for Prokardia and Kardiomics, and Executive Committee member for the CAD Frontiers A2D2 Consortium. JIM was supported by a Postdoctoral Fellowship (No. 108269-2024) from the National Heart Foundation of Australia. MW received consulting fees from Perisphere. LK is a volunteer member in the following entities: Australian Digital health Agency Standards Advisory Group, ISOIEC 22989-22026 Terminology and Concepts for AI in Health, and Standards Australia IT-014-21. EP is supported by a Heart Foundation Future Fellowship (2024-28, ref: 107210), a commissioned research project (2024, $157,380), and a National Health and Medical Research Council, NHMRC, Synergy Grant (2024-28. ref: 2026319). EP is a member of the PHASES (preventing heart attacks and stroke through surveillance) Evaluation and Research Advisory Group, 2025-current (unpaid)—Country to Coast Primary Health Network; a member of the Heart Foundation Dynamics Simulation Model Expert Advisory Panel, 2024 (unpaid)—National Heart Foundation of Australia; and a member of the Australian Cardiovascular Alliance Big Data Flagship Advisory Group, 2020-current (unpaid). MI is a trustee of the Public Health Genomics (PHG) Foundation, member of the Scientific Advisory Board of Open Targets, and has research collaborations with AstraZeneca and Nightingale Health.
