# Supplementary material for: Integration of lipidomic and polygenic risk scores within contemporary clinical cardiovascular risk assessment pathways: a multi-cohort development and validation study
Source: eClinicalMedicine. 2026 Aug 24;99:104159. doi: 10.1016/j.eclinm.2026.104159 (PMC13528312; doi:10.1016/j.eclinm.2026.104159)
Supplement: Supplementary file 1 [file mmc1.pdf]

# Supplementary Material

## Table of Contents


**Supplementary Methods** .......... 1

**Imputation of lipid species not measured in BHS** .......... 1

**Rationale for residualisation of LS and PGS** .......... 1

**Calibration** .......... 1

**Net Reclassification Improvement (NRI)** .......... 2

**Decision-Curve Analysis and Net Benefit** .......... 2

**Additional sample size considerations** .......... 2

**Supplementary Tables** .......... 4

**Table S1. ICD-10-AM codes used to define total cardiovascular outcome.** .......... 4

**Table S2. Baseline survival, mean risk factor values, and recalibration factors for AusCVDRisk.** .......... 6

**Table S3. Cohort-specific genotyping and imputation details.** .......... 6

**Table S4. Lipid species included in the LS model, regularised Cox coefficients, and centring values.** .......... 6

**Table S5. Incremental predictive value of coronary artery and cerebrovascular disease polygenic scores beyond AusCVDRisk and L.CVDRisk.** .......... 16

**Table S6. Regression coefficients for AusCVDRisk predictors used to model the LS.** .......... 17

**Table S7. Regression coefficients for AusCVDRisk predictors used to model the PGS.** .......... 17

**Table S8. Discrimination (Harrell's C-statistic) of AusCVDRisk, L.CVDRisk, G.CVDRisk, and LG.CVDRisk.** .......... 17

**Table S9. Risk stratification by AusCVDRisk, L.CVDRisk, G.CVDRisk, and LG.CVDRisk.** .......... 18

**Table S10. Risk reclassification tables underlying net reclassification improvement analyses.** .......... 19

**Table S11. Incremental discrimination relative to AusCVDRisk: sex-segregated pooled analysis.** .......... 20

**Table S12. Net reclassification improvement relative to AusCVDRisk: sex-segregated pooled analysis.** .......... 20

**Table S13. Calibration intercepts and slopes for four risk models: sex-segregated pooled analysis.** .......... 20

**Supplementary Figures** .......... 21

**Figure S1. Flowcharts for derivation cohorts (ages 35-85). Participant selection and exclusion for AusDiab and BHS.** .......... 21

**Figure S2. Flowcharts for internal and external validation cohorts (ages 45-79, or 35-79 for participants with diabetes). Participant selection and exclusion for AusDiab, BHS, MCCS, ASPREE, and BioHEART.** .......... 22

**Figure S3. Evaluation of lipid imputation performance.** .......... 23

**Figure S4. Standardised coefficients from the LS regularised Cox regression model.** .......... 24

**Figure S5. Distribution of observed and predicted lipidomic scores (LS) across five cohorts.** .......... 25

**Figure S6. Diagnostics for Cox proportional hazard regression models.** .......... 25

**Figure S7. Calibration plots.** .......... 26

**Figure S8. CVD risk stratification using AusCVDRisk, L.CVDRisk, G.CVDRisk, and LG.CVDRisk in full cohorts (ages 45-79, or 35-79 for those with diabetes).** .......... 27

**Figure S9. Decision-curve analysis of AusCVDRisk, L.CVDRisk, G.CVDRisk, and LG.CVDRisk as stand-alone risk assessment tools.** .......... 28

## Supplementary Methods

### Imputation of lipid species not measured in BHS

The lipidomic platform used in the Busselton Health Study (BHS) quantified 550 lipid species that were also measured in the AusDiab cohort but did not include 139 additional lipid species available in AusDiab. To facilitate application of the lipidomic score across cohorts, these 139 lipid species were imputed using elastic-net regression models, following a strategy similar to that described by Dakic et al. for harmonisation of lipidomic datasets.[1]

For each of the 139 lipid species, a separate elastic-net model was developed in the AusDiab cohort. The target lipid species served as the outcome, while the 550 lipid species measured in both AusDiab and BHS, together with age, sex, and diabetes status, were used as predictors. Models were fitted using the glmnet package in R, with regularisation parameters selected by cross-validation. The final models were subsequently applied to BHS to generate predicted concentrations for the 139 unmeasured lipid species.

Prediction accuracy was assessed by comparing measured and predicted lipid concentrations in external cohorts where all 689 lipid species were available (MCCS, ASPREE, and BioHEART). Because the 139 target lipid species were not measured in BHS, direct validation was not possible in this cohort. Therefore, we additionally evaluated prediction performance using lipid species measured in BHS, under the assumption that prediction accuracy would be similar for lipid species with comparable correlation structure. Results of these validation analyses are shown in Figure S3.

### Rationale for residualisation of LS and PGS

Residualisation was performed to estimate the predictive contribution of the lipidomic score (LS) and polygenic score (PGS) beyond information already contained within the AusCVDRisk model. This approach is conceptually similar to the "predictor add-on" methodology described by Matsushita et al.,[2–4] where new predictors are added to an established risk equation while preserving the original model structure.

The need for residualisation arises because AusCVDRisk was incorporated into the models as a fixed offset. The molecular scores may be associated with variables already included in AusCVDRisk and therefore partly reflect the same underlying risk information. Because the AusCVDRisk coefficients are fixed in this setting, this overlap cannot be accounted for during model fitting. Without residualisation, part of the predictive contribution attributed to LS or PGS may therefore reflect information already captured by the clinical model, effectively resulting in double counting of risk information.

This issue was particularly relevant for the LS, which showed substantial associations with age, sex, diabetes, and the total-to-HDL cholesterol ratio. Provided the residualisation model adequately captures the relationships between LS and conventional risk factors, it removes the component of LS that can be predicted from these variables, while retaining the remaining variation not explained by them. The resulting residual score (ΔLS) therefore represents lipidomic information beyond that already captured by AusCVDRisk. A similar procedure was applied to the PGS, although the overlap between genomic and clinical predictors was substantially weaker.

When residualisation models are applied to external cohorts, differences in participant characteristics may occasionally result in systematic over- or under-prediction of the molecular score. In such situations, recalibration by centring predicted values to the cohort-specific mean has been recommended and applied in previous predictor add-on studies.[3,4] This adjustment reflects the principle that a well-calibrated prediction model should, on average, reproduce the mean value of the target variable in the population to which it is applied.

In the present study, predicted LS values in ASPREE were systematically higher than the corresponding observed LS values (Figure S5), indicating a location shift between predicted and observed score distributions. This likely reflects differences between ASPREE and the AusDiab derivation cohort, particularly the substantially older age of ASPREE participants. Therefore, predicted LS values were centred to the cohort-specific mean prior to calculation of ΔLS. This adjustment corrected the systematic bias in predicted LS while preserving the between-individual variation used for risk prediction.

### Calibration

Calibration-in-the-large (intercept) and calibration slope were estimated using Poisson calibration models applied to the predicted log cumulative hazard at 5 years, as described by Crowson et al.[5] Predicted risks ($p_t$) were transformed to the cumulative hazards ($H_t = -log(1 - p_t)$) and log transformed ($\eta = log(H_t)$). Calibration-in-the-large was estimated from a Poisson model with $\eta$ included as an offset term, whereas the calibration slope was estimated by fitting $\eta$ as a predictor.

**Net Reclassification Improvement (NRI)**

Net reclassification improvement (NRI) was used to quantify changes in risk classification when L.CVDRisk, G.CVDRisk, and LG.CVDRisk were compared with AusCVDRisk.

For uncensored data, overall NRI is defined as $\text{NRI} = \text{NRI}_{+} + \text{NRI}_{-}$

The event and non-event components are

$$\text{NRI}_{+} = P(up|event) - P(down|event)$$
$$\text{NRI}_{-} = P(down|non\ event) - P(up|non\ event)$$

where "up" and "down" denote movement to a higher or lower risk category under the enhanced model relative to AusCVDRisk.

Event NRI quantifies the net proportion of future CVD events correctly reclassified to higher risk categories and may be interpreted as a measure of improvement in sensitivity. Non-event NRI quantifies the net proportion of non-events correctly reclassified to lower risk categories and may be interpreted as a measure of improvement in specificity. Because overall NRI combines gains in sensitivity and specificity into a single metric, it could not be expresses as a % improvement.

For time-to-event outcomes, direct classification of participants as events or non-events is complicated by censoring. Therefore, NRI was estimated using the survival-data extension proposed by Pencina et al.,[6] in which event probabilities at the 5-year time horizon are estimated using survival methods rather than simple proportions. In practice, observed event probabilities were estimated using inverse probability of censoring weighting, following the framework of Uno et al.,[7] which provides consistent estimation and inference for reclassification measures in the presence of censoring.

**Decision-Curve Analysis and Net Benefit**

Decision-curve analysis was used to evaluate the potential clinical utility of molecular testing strategies across a range of treatment thresholds. Net benefit combines true-positive and false-positive classifications into a single metric that reflects the clinical consequences of treatment decisions. It is defined as:

$$Net\ benefit = \frac{True\ positives}{N} - \frac{False\ positives}{N}\frac{p_T}{1 - p_T}$$

where $N$ is the total sample size, and $p_T$ is the risk threshold at which treatment would be recommended. The weighting term $\frac{p_T}{1-p_T}$ represents the relative harm of a false-positive classification compared with the benefit of correctly identifying an individual who will experience a CVD event. For example, at a treatment threshold of $p_T = 0.1$, the benefit of correctly identifying an individual who would experience a CVD event is considered approximately nine times greater than the harm of unnecessarily classifying/treating an individual who would not experience an event. Equivalently, this threshold implies that approximately nine false-positive classifications are considered acceptable for each additional future CVD event correctly identified. The 10% threshold corresponds to the current treatment threshold used in the Australian CVD prevention guidelines.

Net benefit may therefore be interpreted as the proportion of additional true-positive classifications achieved after accounting for the clinical consequences of unnecessary treatment. For example, a net benefit of 0.01 corresponds to the equivalent of 10 additional correctly identified events per 1,000 individuals without increasing the number of unnecessary treatments.

**Additional sample size considerations**

Conventional sample size calculations for prediction models focus on avoiding overfitting, obtaining stable predictor estimates, and ensuring accurate estimation of absolute risk. These considerations are less directly applicable to the present study because the omics-enriched models were not developed de novo. Instead, the established AusCVDRisk linear predictor, originally derived in approximately 300,000 individuals, was incorporated as a fixed offset. Consequently, the derivation cohorts were used only to estimate the incremental contribution of lipidomic and genomic information beyond the guideline-recommended risk equation, rather than to re-estimate all clinical predictor effects. This approach substantially reduces model complexity and the risk of overfitting.

As a formal assessment, we additionally applied the sample size framework proposed by Riley et al. using the R package pmsampsize.[8,9] Assuming an event rate of 0.011 events per person-year, mean follow-up of 3.5 years (AUS-PREDICT cohort[10]), and Nagelkerke $R^2$ values of 0.099 and 0.161 (corresponding to validation performance of AusCVDRisk in AusDiab and BHS, respectively), the minimum required sample size was estimated as 317 participants with 13 events and 195 participants with 8 events. Applying cohort-specific event rates and follow-up durations yielded similarly modest requirements (AusDiab: n = 405 and 12 events; BHS: n = 165 and 9 events). These requirements were substantially lower than the available derivation sample sizes and event counts.

Finally, external validation provided empirical evidence that model optimism was limited. Calibration slopes were close to or above 1.0 across validation cohorts, providing no evidence of substantial overfitting. Together, these findings indicate that the available derivation sample sizes were more than adequate for estimating the incremental predictive value of lipidomic and genomic information beyond the established AusCVDRisk model.

**References:**


1. Dakic A, Wu J, Wang T, et al. Imputation of plasma lipid species to facilitate integration of lipidomic datasets. *Nat Commun*. 2024;15:1540.

2. Matsushita K, Sang Y, Chen J, et al. Novel "Predictor Patch" Method for Adding Predictors Using Estimates From Outside Datasets — A Proof-of-Concept Study Adding Kidney Measures to Cardiovascular Mortality Prediction —. *Circulation Journal*. 2019;83:1876–1882.

3. Matsushita K, Jassal SK, Sang Y, et al. Incorporating kidney disease measures into cardiovascular risk prediction: Development and validation in 9 million adults from 72 datasets. *eClinicalMedicine*. 2020;27.

4. Matsushita K, Kaptoge S, Hageman SHJ, et al. Including measures of chronic kidney disease to improve cardiovascular risk prediction by SCORE2 and SCORE2-OP. *European Journal of Preventive Cardiology*. 2023;30:8–16.

5. Crowson CS, Atkinson EJ, Therneau TM. Assessing calibration of prognostic risk scores. *Stat Methods Med Res*. 2016;25:1692–1706.

6. Pencina MJ, D'Agostino Sr RB, Steyerberg EW. Extensions of net reclassification improvement calculations to measure usefulness of new biomarkers. *Statistics in Medicine*. 2011;30:11–21.

7. Uno H, Tian L, Cai T, Kohane IS, Wei LJ. A unified inference procedure for a class of measures to assess improvement in risk prediction systems with survival data. *Statistics in Medicine*. 2013;32:2430–2442.

8. Riley RD, Snell KI, Ensor J, et al. Minimum sample size for developing a multivariable prediction model: PART II - binary and time-to-event outcomes. *Stat Med*. 2019;38:1276–1296.

9. Riley RD, Ensor J, Snell KIE, et al. Calculating the sample size required for developing a clinical prediction model. *BMJ*. 2020;368:m441.

10. Paige E, Banks Am E, Zhang Y, et al. Development and calibration of the 2023 Australian cardiovascular disease risk prediction equations: a model updating study. *Med J Aust*. 2025;223:197–204.

## Supplementary Tables

### Table S1. ICD-10-AM codes used to define total cardiovascular outcome.

| Outcome type | Code | ICD-10-AM code descriptor |
|---|---|---|
| Myocardial infarction (MI) | I21.0 | Acute transmural myocardial infarction of anterior wall |
| | I21.1 | Acute transmural myocardial infarction of inferior wall |
| | I21.2 | Acute transmural myocardial infarction of other sites |
| | I21.3 | Acute transmural myocardial infarction of unspecified site |
| | I21.4 | Acute subendocardial myocardial infarction |
| | I21.9 | Acute myocardial infarction, unspecified |
| | I22.0 | Subsequent myocardial infarction of anterior wall |
| | I22.1 | Subsequent myocardial infarction of inferior wall |
| | I22.8 | Subsequent myocardial infarction of other sites |
| | I22.9 | Subsequent myocardial infarction of unspecified site |
| Unstable angina | I20.0 | Unstable angina |
| Other coronary heart disease | I20.1 | Angina pectoris with documented spasm |
| | I20.8 | Other forms of angina pectoris |
| | I20.9 | Angina pectoris, unspecified |
| | I23.0 | Haemopericardium as current complication following acute myocardial infarction |
| | I23.1 | Atrial septal defect as current complication following acute myocardial infarction |
| | I23.2 | Ventricular septal defect as current complication following acute myocardial infarction |
| | I23.3 | Rupture of cardiac wall without haemopericardium as current complication following acute MI |
| | I23.4 | Rupture of chordae tendineae as current complication following acute myocardial infarction |
| | I23.5 | Rupture of papillary muscle as current complication following acute myocardial infarction |
| | I23.6 | Thrombosis of atrium, auricular appendage, and ventricle as current complications following acute MI |
| | I23.8 | Other current complications following acute myocardial infarction |
| | I24.0 | Coronary thrombosis not resulting in myocardial infarction |
| | I24.1 | Dressler's syndrome |
| | I24.8 | Other forms of acute ischaemic heart disease |
| | I24.9 | Acute ischaemic heart disease, unspecified |
| | I25.3 | Aneurysm of heart |
| | I25.4 | Coronary artery aneurysm and dissection |
| | I25.5 | Ischaemic cardiomyopathy |
| | I25.6 | Silent myocardial ischaemia |
| | I46.0 | Cardiac arrest with successful resuscitation |
| | I46.9 | Cardiac arrest, unspecified |
| Ischaemic stroke | I63.0 | Cerebral infarction due to thrombosis of precerebral arteries |
| | I63.1 | Cerebral infarction due to embolism of precerebral arteries |
| | I63.2 | Cerebral infarction due to unspecified occlusion or stenosis of precerebral arteries |
| | I63.3 | Cerebral infarction due to thrombosis of cerebral arteries |
| | I63.4 | Cerebral infarction due to embolism of cerebral arteries |
| | I63.5 | Cerebral infarction due to unspecified occlusion or stenosis of cerebral arteries |
| | I63.6 | Cerebral infarction due to cerebral venous thrombosis, nonpyogenic |
| | I63.8 | Other cerebral infarction |
| | I63.9 | Cerebral infarction, unspecified |
| | I64 | Stroke, not specified as haemorrhage or infarction |
| Haemorrhagic stroke | I60.0 | Subarachnoid haemorrhage from carotid siphon and bifurcation |
| | I60.1 | Subarachnoid haemorrhage from middle cerebral artery |
| | I60.2 | Subarachnoid haemorrhage from anterior communicating artery |
| | I60.3 | Subarachnoid haemorrhage from posterior communicating artery |
| | I60.4 | Subarachnoid haemorrhage from basilar artery |
| | I60.5 | Subarachnoid haemorrhage from vertebral artery |
| | I60.6 | Subarachnoid haemorrhage from other intracranial arteries |
| | I60.7 | Subarachnoid haemorrhage from intracranial artery, unspecified |
| | I60.8 | Other subarachnoid haemorrhage |
| | I60.9 | Subarachnoid haemorrhage, unspecified |
| | I61.0 | Intracerebral haemorrhage in hemisphere, subcortical |
| | I61.1 | Intracerebral haemorrhage in hemisphere, cortical |
| | I61.2 | Intracerebral haemorrhage in hemisphere, unspecified |
| | I61.3 | Intracerebral haemorrhage in brain stem |
| | I61.4 | Intracerebral haemorrhage in cerebellum |
| | I61.5 | Intracerebral haemorrhage, intraventricular |
| | I61.6 | Intracerebral haemorrhage, multiple localised |
| | I61.8 | Other intracerebral haemorrhage |
| | I61.9 | Intracerebral haemorrhage, unspecified |
| Transient ischaemic attack | G45.0 | Vertebro-basilar artery syndrome |
| | G45.1 | Carotid artery syndrome (hemispheric) |
| | G45.2 | Multiple and bilateral precerebral artery syndromes |
| | G45.3 | Amaurosis fugax |
| | G45.8 | Other transient cerebral ischaemic attacks and related syndromes |
| | G45.9 | Transient cerebral ischaemic attack, unspecified |
| | G46.0 | Middle cerebral artery syndrome (I66.0+) |
| | G46.1 | Anterior cerebral artery syndrome (I66.1+) |
| | G46.2 | Posterior cerebral artery syndrome (I66.2+) |
| | G46.3 | Brain stem stroke syndrome (I60-I67+) |
| | G46.4 | Cerebellar stroke syndrome (I60-I67+) |
| | G46.5 | Pure motor lacunar syndrome (I60-I67+) |
| | G46.6 | Pure sensory lacunar syndrome (I60-I67+) |

| Outcome type | Code | ICD-10-AM code descriptor |
|---|---|---|
| | G46.7 | Other lacunar syndromes (I60-I67+) |
| | G46.8 | Other vascular syndromes of brain in cerebrovascular diseases (I60-I67+) |
| Peripheral vascular disease | E10.51 | Type 1 diabetes mellitus with peripheral angiopathy, without gangrene |
| | E10.52 | Type 1 diabetes mellitus with peripheral angiopathy, with gangrene |
| | E11.51 | Type 2 diabetes mellitus with peripheral angiopathy, without gangrene |
| | E11.52 | Type 2 diabetes mellitus with peripheral angiopathy, with gangrene |
| | E14.51 | Unspecified diabetes mellitus with peripheral angiopathy, without gangrene |
| | E14.52 | Unspecified diabetes mellitus with peripheral angiopathy, with gangrene |
| | I70.21 | Atherosclerosis of arteries of extremities with intermittent claudication |
| | I70.22 | Atherosclerosis of arteries of extremities with rest pain |
| | I70.23 | Atherosclerosis of arteries of extremities with ulceration |
| | I70.24 | Atherosclerosis of arteries of extremities with gangrene |
| | I71.00 | Dissection of aorta, unspecified site |
| | I71.01 | Dissection of thoracic aorta |
| | I71.02 | Dissection of abdominal aorta |
| | I71.03 | Dissection of thoracoabdominal aorta |
| | I71.1 | Thoracic aortic aneurysm, ruptured |
| | I71.3 | Abdominal aortic aneurysm, ruptured |
| | I71.5 | Thoracoabdominal aortic aneurysm, ruptured |
| | I71.8 | Aortic aneurysm of unspecified site, ruptured |
| | I73.9 | Peripheral vascular disease, unspecified |
| | I74.0 | Embolism and thrombosis of abdominal aorta |
| | I74.1 | Embolism and thrombosis of other and unspecified parts of aorta |
| | I74.2 | Embolism and thrombosis of arteries of upper extremities |
| | I74.3 | Embolism and thrombosis of arteries of lower extremities |
| | I74.4 | Embolism and thrombosis of arteries of extremities, unspecified |
| | I74.5 | Embolism and thrombosis of iliac artery |
| | I74.8 | Embolism and thrombosis of other arteries |
| | I74.9 | Embolism and thrombosis of unspecified artery |
| Congestive heart failure | I11.0 | Hypertensive heart disease with (congestive) heart failure |
| | I13.0 | Hypertensive heart and kidney disease with (congestive) heart failure |
| | I13.2 | Hypertensive heart and kidney disease with both (congestive) heart failure and kidney failure |
| | I50.0 | Congestive heart failure |
| | I50.1 | Left ventricular failure |
| | I50.9 | Heart failure, unspecified |
| Other ischaemic CVD-related (Deaths only) | E10.59 | Type 1 diabetes mellitus with other circulatory complications |
| | E11.59 | Type 2 diabetes mellitus with other circulatory complications |
| | E14.59 | Unspecified diabetes mellitus with other specified circulatory complication |
| | I25.0 | Atherosclerotic cardiovascular disease, so described |
| | I25.10 | Atherosclerotic heart disease, of unspecified vessel |
| | I25.11 | Atherosclerotic heart disease, of native coronary artery |
| | I25.12 | Atherosclerotic heart disease, of autologous bypass graft |
| | I25.13 | Atherosclerotic heart disease, of nonautologous bypass graft |
| | I25.2 | Old myocardial infarction |
| | I25.8 | Other forms of chronic ischaemic heart disease |
| | I25.9 | Chronic ischaemic heart disease, unspecified |
| | I46.1 | Sudden cardiac death, so described |
| | I65.0 | Occlusion and stenosis of vertebral artery |
| | I65.1 | Occlusion and stenosis of basilar artery |
| | I65.2 | Occlusion and stenosis of carotid artery |
| | I65.3 | Occlusion and stenosis of multiple and bilateral precerebral arteries |
| | I65.8 | Occlusion and stenosis of other precerebral artery |
| | I65.9 | Occlusion and stenosis of unspecified precerebral artery |
| | I66.0 | Occlusion and stenosis of middle cerebral artery |
| | I66.1 | Occlusion and stenosis of anterior cerebral artery |
| | I66.2 | Occlusion and stenosis of posterior cerebral artery |
| | I66.3 | Occlusion and stenosis of cerebellar arteries |
| | I66.4 | Occlusion and stenosis of multiple and bilateral cerebral arteries |
| | I66.8 | Occlusion and stenosis of other cerebral artery |
| | I66.9 | Occlusion and stenosis of unspecified cerebral artery |
| | I67.0 | Dissection of cerebral arteries, nonruptured |
| | I67.2 | Cerebral atherosclerosis |
| | I69.0 | Sequelae of subarachnoid haemorrhage |
| | I69.1 | Sequelae of intracerebral haemorrhage |
| | I69.3 | Sequelae of cerebral infarction |
| | I69.4 | Sequelae of stroke, not specified as haemorrhage or infarction |
| | I69.8 | Sequelae of other and unspecified cerebrovascular diseases |
| | I70.0 | Atherosclerosis of aorta |
| | I70.1 | Atherosclerosis of renal artery |
| | I70.20 | Atherosclerosis of arteries of extremities, unspecified |
| | I70.8 | Atherosclerosis of other arteries |
| | I70.9 | Generalised and unspecified atherosclerosis |
| | I71.4 | Abdominal aortic aneurysm, without mention of rupture |
| | Z95.1 | Presence of aortocoronary bypass graft |
| | Z95.5 | Presence of coronary angioplasty implant and graft |
| | Z95.8 | Presence of other cardiac and vascular implants and grafts |
| | Z95.9 | Presence of cardiac and vascular implant and graft, unspecified |

ICD-10-AM, International Statistical Classification of Diseases and Related Health Problems, Tenth Revision, Australian Modification

**Table S2. Baseline survival, mean risk factor values, and recalibration factors for AusCVDRisk.**

| | | Women | Men |
|---|---|---|---|
| **Baseline survival function (5 year)** | | 0.9828361 | 0.9728782 |
| **Mean values used for centring** | Age (years) | 58.47472 | 53.77579 |
| | SEIFA (quintile) | 2.625575 | 2.628206 |
| | SBP (mmHg) | 129.2052 | 129.1706 |
| | Total/HDL cholesterol ratio | 3.641988 | 4.325589 |
| **Recalibration factors in AusCVDRisk equation** | 30-44 years age group | 1.3 | 1.1 |
| | 45-49 | 1.2 | 1.1 |
| | 50-54 | 1.2 | 1.1 |
| | 55-59 | 1.0 | 1.0 |
| | 60-64 | 0.9 | 0.9 |
| | 65-69 | 0.8 | 0.8 |
| | 70-74 | 0.7 | 0.8 |
| | 75-79 | 0.7 | 0.8 |

**Table S3. Cohort-specific genotyping and imputation details.**

| Cohort | Genotyping array | Reference panel | Genome build | Post-imputation QC cutoff |
|---|---|---|---|---|
| BHS | Illumina Human 610K Quad-Bead Chip; Illumina 660 W Quad Array | HRC r1.1 | GRCh37 (hg19) | $r^2 > 0.80$ |
| MCCS | Illumina Global Screening Array-24 v3 (GSA-24v3) | HRC r1.1 | GRCh37 (hg19) | $r^2 > 0.30$ |
| ASPREE | Axiom 2.0 Precision Medicine Diversity Research Array | TOPMed r2 | GRCh38 (hg38) | $r^2 > 0.30$ |
| BioHeart | Standard Germline Exome v6 Plus GSA v1 Array | TOPMed r2 | GRCh38 (hg38) | $r^2 > 0.80$ |

QC, quality control; HRC, Haplotype Reference Consortium; TOPMed, Trans-Omics for Precision Medicine; GRCh37/38, Genome Reference Consortium Human Build 37/39; hg19/38, Human Genome version 19/38

**Table S4. Lipid species included in the LS model, regularised Cox coefficients, and centring values.**

| Lipid | Class | Imputed in BHS | Coefficient | Mean log-value |
|---|---|---|---|---|
| AC 12:0 | AC | 0 | 0.092 | 3.883 |
| AC 13:0 | AC | 0 | 0.016 | 2.691 |
| AC 14:0 | AC | 0 | 0.002 | 2.896 |
| AC 14:1 | AC | 0 | 0.046 | 3.702 |
| AC 14:2 | AC | 0 | 0.056 | 2.889 |
| AC 15:0 [a] | AC | 0 | 0.052 | 0.916 |
| AC 15:0 [b] | AC | 0 | 0.000 | 0.512 |
| AC 16:0 | AC | 0 | 0.000 | 4.266 |
| AC 16:1 | AC | 0 | 0.053 | 2.837 |
| AC 18:0 | AC | 0 | 0.089 | 3.067 |
| AC 18:1 | AC | 0 | 0.113 | 4.368 |
| AC 18:2 | AC | 0 | 0.053 | 3.201 |
| CE 14:0 | CE | 0 | 0.139 | 10.247 |
| CE 15:0 | CE | 0 | 0.125 | 9.278 |
| CE 16:0 | CE | 0 | 0.225 | 12.427 |
| CE 16:1 | CE | 0 | 0.000 | 11.635 |
| CE 16:2 | CE | 0 | 0.027 | 7.539 |
| CE 17:0 | CE | 0 | -0.005 | 9.506 |
| CE 17:1 | CE | 0 | 0.014 | 9.494 |
| CE 18:0 | CE | 0 | -0.051 | 10.403 |
| CE 18:1 | CE | 0 | 0.006 | 13.511 |
| CE 18:2 | CE | 0 | 0.000 | 14.522 |
| CE 18:3 | CE | 0 | 0.063 | 11.619 |
| CE 20:0 | CE | 0 | 0.023 | 5.886 |
| CE 20:1 | CE | 0 | 0.000 | 6.339 |
| CE 20:2 | CE | 0 | 0.290 | 7.195 |
| CE 20:3 | CE | 0 | 0.019 | 10.834 |
| CE 20:4 | CE | 0 | 0.000 | 12.424 |
| CE 20:5 | CE | 0 | 0.034 | 10.870 |
| CE 22:0 | CE | 0 | -0.050 | 4.652 |
| CE 22:1 | CE | 0 | 0.000 | 4.556 |
| CE 22:4 | CE | 0 | 0.000 | 7.228 |
| CE 22:6 | CE | 0 | -0.001 | 11.298 |
| CE 24:0 | CE | 0 | 0.073 | 4.256 |
| CE 24:1 | CE | 0 | -0.009 | 4.583 |
| CE 24:4 | CE | 0 | -0.103 | 3.905 |
| CE 24:5 | CE | 0 | -0.047 | 4.446 |
| CE 24:6 | CE | 0 | 0.000 | 4.917 |
| Cer 16:1;O2/16:0 | Cer | 0 | -0.106 | 3.189 |
| Cer 16:1;O2/18:0 | Cer | 0 | -0.025 | 3.233 |
| Cer 16:1;O2/20:0 | Cer | 0 | 0.064 | 3.345 |
| Cer 16:1;O2/22:0 | Cer | 0 | 0.000 | 4.821 |
| Cer 16:1;O2/23:0 | Cer | 1 | 0.000 | 4.366 |
| Cer 16:1;O2/24:0 | Cer | 0 | -0.055 | 5.563 |

| Lipid | Class | Imputed in BHS | Coefficient | Mean log-value |
|---|---|---|---|---|
| Cer 16:1;O2/24:1 | Cer | 0 | 0.000 | 4.503 |
| Cer 17:1;O2/16:0 | Cer | 0 | 0.000 | 2.987 |
| Cer 17:1;O2/18:0 | Cer | 0 | -0.096 | 2.040 |
| Cer 17:1;O2/20:0 | Cer | 0 | 0.073 | 1.725 |
| Cer 17:1;O2/22:0 | Cer | 0 | 0.000 | 4.046 |
| Cer 17:1;O2/23:0 | Cer | 1 | 0.004 | 4.078 |
| Cer 17:1;O2/24:0 | Cer | 0 | 0.000 | 5.104 |
| Cer 17:1;O2/24:1 | Cer | 0 | 0.032 | 4.498 |
| Cer 18:0;O/20:0 | deoxyCer | 1 | 0.000 | 2.762 |
| Cer 18:0;O/22:0 | deoxyCer | 1 | 0.010 | 3.961 |
| Cer 18:0;O/23:0 | deoxyCer | 1 | 0.072 | 3.271 |
| Cer 18:0;O/24:0 | deoxyCer | 1 | 0.000 | 4.151 |
| Cer 18:0;O/24:1 | deoxyCer | 1 | 0.029 | 3.868 |
| Cer 18:0;O2/16:0 | dhCer | 0 | 0.114 | 3.974 |
| Cer 18:0;O2/20:0 | dhCer | 0 | -0.015 | 3.560 |
| Cer 18:0;O2/22:0 | dhCer | 0 | -0.085 | 5.042 |
| Cer 18:0;O2/24:0 | dhCer | 0 | -0.152 | 5.248 |
| Cer 18:0;O2/24:1 | dhCer | 0 | 0.082 | 4.935 |
| Cer 18:1;O/18:0 | deoxyCer | 1 | 0.064 | 2.107 |
| Cer 18:1;O/20:0 | deoxyCer | 1 | 0.178 | 3.688 |
| Cer 18:1;O/22:0 | deoxyCer | 1 | 0.104 | 4.571 |
| Cer 18:1;O/23:0 | deoxyCer | 1 | 0.121 | 3.846 |
| Cer 18:1;O/24:0 | deoxyCer | 1 | 0.122 | 4.695 |
| Cer 18:1;O/24:1 | deoxyCer | 1 | 0.125 | 4.329 |
| Cer 18:1;O2/14:0 | Cer | 0 | -0.007 | 2.353 |
| Cer 18:1;O2/16:0 | Cer | 0 | 0.062 | 5.613 |
| Cer 18:1;O2/18:0 | Cer | 0 | 0.000 | 4.473 |
| Cer 18:1;O2/19:0 | Cer | 1 | 0.148 | 1.420 |
| Cer 18:1;O2/20:0 | Cer | 0 | 0.111 | 4.906 |
| Cer 18:1;O2/21:0 | Cer | 1 | 0.171 | 3.239 |
| Cer 18:1;O2/22:0 | Cer | 0 | 0.000 | 6.989 |
| Cer 18:1;O2/23:0 | Cer | 1 | 0.101 | 6.858 |
| Cer 18:1;O2/24:0 | Cer | 0 | -0.053 | 7.895 |
| Cer 18:1;O2/24:1 | Cer | 0 | 0.067 | 7.260 |
| Cer 18:1;O2/26:0 | Cer | 0 | 0.074 | 3.834 |
| Cer 18:2;O2/14:0 | Cer | 0 | -0.063 | 1.163 |
| Cer 18:2;O2/16:0 | Cer | 0 | 0.000 | 3.544 |
| Cer 18:2;O2/18:0 | Cer | 0 | 0.000 | 3.123 |
| Cer 18:2;O2/20:0 | Cer | 0 | 0.000 | 2.955 |
| Cer 18:2;O2/21:0 | Cer | 1 | 0.072 | 1.254 |
| Cer 18:2;O2/22:0 | Cer | 0 | -0.061 | 5.333 |
| Cer 18:2;O2/23:0 | Cer | 1 | -0.019 | 5.214 |
| Cer 18:2;O2/24:0 | Cer | 0 | -0.093 | 6.393 |
| Cer 18:2;O2/24:1 | Cer | 0 | -0.030 | 5.478 |
| Cer 18:2;O2/26:0 | Cer | 0 | 0.030 | 2.213 |
| Cer 19:1;O2/16:0 | Cer | 0 | -0.050 | 1.196 |
| Cer 19:1;O2/18:0 | Cer | 0 | 0.116 | 1.575 |
| Cer 19:1;O2/20:0 | Cer | 0 | 0.026 | 2.177 |
| Cer 19:1;O2/22:0 | Cer | 0 | -0.052 | 4.561 |
| Cer 19:1;O2/23:0 | Cer | 1 | 0.020 | 4.532 |
| Cer 19:1;O2/24:0 | Cer | 0 | 0.060 | 5.659 |
| Cer 19:1;O2/24:1 | Cer | 0 | 0.031 | 5.080 |
| Cer 19:1;O2/26:0 | Cer | 0 | -0.023 | 1.809 |
| Cer 20:1;O2/22:0 | Cer | 0 | 0.034 | 2.351 |
| Cer 20:1;O2/23:0 | Cer | 1 | 0.056 | 2.222 |
| Cer 20:1;O2/24:0 | Cer | 0 | 0.158 | 3.328 |
| Cer 20:1;O2/24:1 | Cer | 0 | 0.170 | 2.704 |
| COH | COH | 0 | -0.109 | 13.689 |
| DE 16:0 \| SE 27:2/16:0 | DE | 0 | 0.115 | 5.099 |
| DE 18:1 \| SE 27:2/18:1 | DE | 0 | 0.000 | 7.637 |
| DE 18:2 \| SE 27:2/18:2 | DE | 0 | 0.037 | 8.723 |
| DE 20:4 \| SE 27:2/20:4 | DE | 0 | 0.041 | 7.420 |
| DE 20:5 \| SE 27:2/20:5 | DE | 0 | 0.047 | 6.135 |
| DE 22:6 \| SE 27:2/22:6 | DE | 0 | 0.025 | 6.079 |
| DG 14:0_16:0 | DG | 0 | 0.011 | 5.990 |
| DG 16:0_16:1 | DG | 0 | 0.000 | 7.189 |
| DG 16:0_18:1 | DG | 0 | 0.000 | 9.929 |
| DG 16:0_18:2 | DG | 0 | -0.011 | 8.721 |
| DG 16:0_20:4 | DG | 0 | -0.024 | 6.630 |
| DG 16:0_22:5 | DG | 0 | -0.081 | 5.457 |
| DG 16:0_22:6 | DG | 0 | -0.061 | 6.051 |
| DG 16:1_18:1 | DG | 0 | -0.015 | 8.142 |
| DG 18:0_18:1 | DG | 0 | -0.006 | 8.639 |
| DG 18:0_18:2 | DG | 0 | 0.000 | 7.599 |
| DG 18:0_20:4 | DG | 0 | -0.086 | 6.822 |
| DG 18:1_18:1 | DG | 0 | -0.003 | 10.868 |
| DG 18:1_18:2 | DG | 0 | 0.009 | 9.219 |
| DG 18:1_18:3 | DG | 0 | 0.014 | 7.409 |
| DG 18:1_20:3 | DG | 0 | 0.086 | 7.286 |
| DG 18:1_20:4 | DG | 0 | 0.000 | 7.647 |
| DG 18:1_20:5 | DG | 1 | 0.000 | 6.203 |

| Lipid | Class | Imputed in BHS | Coefficient | Mean log-value |
|---|---|---|---|---|
| DG 18:1_22:5 | DG | 1 | 0.000 | 6.196 |
| DG 18:1_22:6 | DG | 1 | -0.014 | 6.753 |
| DG 18:2_18:2 | DG | 0 | 0.023 | 7.327 |
| DG 18:2_20:4 | DG | 0 | 0.003 | 6.680 |
| DG 18:2_22:6 | DG | 1 | 0.011 | 5.926 |
| GM1 18:1;O2/16:0 | GM1 | 0 | -0.137 | 2.582 |
| GM3 18:1;O2/16:0 | GM3 | 0 | 0.214 | 6.452 |
| GM3 18:1;O2/18:0 | GM3 | 0 | -0.291 | 5.713 |
| GM3 18:1;O2/20:0 | GM3 | 0 | 0.055 | 4.918 |
| GM3 18:1;O2/22:0 | GM3 | 0 | 0.063 | 6.222 |
| GM3 18:1;O2/24:0 | GM3 | 0 | -0.012 | 5.786 |
| GM3 18:1;O2/24:1 | GM3 | 0 | 0.067 | 6.451 |
| Hex-Cer 16:1;O2/18:0 | HexCer | 0 | -0.078 | 2.744 |
| Hex-Cer 16:1;O2/20:0 | HexCer | 0 | 0.053 | 2.792 |
| Hex-Cer 16:1;O2/22:0 | HexCer | 0 | -0.013 | 3.783 |
| Hex-Cer 16:1;O2/24:0 | HexCer | 0 | -0.032 | 3.808 |
| Hex-Cer 18:1;O2/16:0 | HexCer | 0 | 0.123 | 7.125 |
| Hex-Cer 18:1;O2/18:0 | HexCer | 0 | -0.001 | 4.167 |
| Hex-Cer 18:1;O2/20:0 | HexCer | 0 | 0.029 | 4.117 |
| Hex-Cer 18:1;O2/22:0 | HexCer | 0 | -0.031 | 6.452 |
| Hex-Cer 18:1;O2/24:0 | HexCer | 0 | 0.000 | 7.124 |
| Hex-Cer 18:1;O2/24:1 | HexCer | 0 | 0.000 | 6.509 |
| Hex-Cer 18:2;O2/18:0 | HexCer | 0 | -0.047 | 2.186 |
| Hex-Cer 18:2;O2/20:0 | HexCer | 0 | -0.017 | 2.487 |
| Hex-Cer 18:2;O2/22:0 | HexCer | 0 | -0.016 | 4.337 |
| Hex-Cer 18:2;O2/24:0 | HexCer | 0 | 0.000 | 4.962 |
| Hex2Cer 16:1;O2/16:0 | Hex2Cer | 0 | -0.032 | 4.736 |
| Hex2Cer 16:1;O2/24:1 | Hex2Cer | 0 | -0.087 | 3.092 |
| Hex2Cer 18:1;O2/16:0 | Hex2Cer | 0 | -0.075 | 7.816 |
| Hex2Cer 18:1;O2/20:0 | Hex2Cer | 0 | 0.085 | 3.181 |
| Hex2Cer 18:1;O2/22:0 | Hex2Cer | 0 | 0.163 | 4.825 |
| Hex2Cer 18:1;O2/24:0 | Hex2Cer | 0 | 0.047 | 5.496 |
| Hex2Cer 18:1;O2/24:1 | Hex2Cer | 0 | 0.000 | 6.112 |
| Hex2Cer 18:2;O2/16:0 | Hex2Cer | 0 | 0.045 | 5.131 |
| Hex2Cer 18:2;O2/24:1 | Hex2Cer | 0 | 0.000 | 4.460 |
| Hex3Cer 18:1;O2/16:0 | Hex3Cer | 0 | -0.061 | 6.362 |
| Hex3Cer 18:1;O2/18:0 | Hex3Cer | 0 | 0.179 | 5.486 |
| Hex3Cer 18:1;O2/20:0 | Hex3Cer | 0 | -0.056 | 3.541 |
| Hex3Cer 18:1;O2/22:0 | Hex3Cer | 0 | 0.183 | 4.632 |
| Hex3Cer 18:1;O2/24:0 | Hex3Cer | 0 | 0.083 | 4.938 |
| Hex3Cer 18:1;O2/24:1 | Hex3Cer | 0 | 0.133 | 5.268 |
| LPC 0:0/14:0 | LPC | 0 | 0.090 | 6.133 |
| LPC 0:0/15:0 | LPC | 0 | 0.000 | 6.201 |
| LPC 0:0/16:0 | LPC | 0 | -0.004 | 10.419 |
| LPC 0:0/16:0;Me | LPC | 0 | -0.079 | 5.749 |
| LPC 0:0/16:1 | LPC | 0 | -0.066 | 6.525 |
| LPC 0:0/17:1 [a] | LPC | 0 | 0.000 | 4.417 |
| LPC 0:0/18:0 | LPC | 0 | -0.046 | 9.301 |
| LPC 0:0/18:1 | LPC | 0 | -0.185 | 8.993 |
| LPC 0:0/18:2 | LPC | 0 | -0.224 | 8.887 |
| LPC 0:0/18:3 [a] | LPC | 0 | 0.006 | 4.528 |
| LPC 0:0/19:0 [a] | LPC | 0 | 0.033 | 2.540 |
| LPC 0:0/20:0 | LPC | 0 | -0.115 | 3.644 |
| LPC 0:0/20:1 | LPC | 0 | 0.012 | 4.392 |
| LPC 0:0/20:2 | LPC | 0 | 0.071 | 4.406 |
| LPC 0:0/20:3 | LPC | 0 | -0.128 | 6.675 |
| LPC 0:0/20:4 | LPC | 0 | -0.019 | 7.584 |
| LPC 0:0/20:5 | LPC | 0 | -0.008 | 5.511 |
| LPC 0:0/22:0 | LPC | 0 | -0.171 | 2.297 |
| LPC 0:0/22:1 | LPC | 0 | 0.194 | 1.962 |
| LPC 0:0/22:4 | LPC | 0 | 0.000 | 3.951 |
| LPC 0:0/22:5 [n3] | LPC | 0 | -0.072 | 5.154 |
| LPC 0:0/22:6 | LPC | 0 | 0.026 | 6.152 |
| LPC 0:0/24:0 | LPC | 0 | 0.006 | 2.972 |
| LPC 0:0/26:0 | LPC | 0 | 0.055 | 2.290 |
| LPC 14:0/0:0 | LPC | 0 | 0.069 | 7.268 |
| LPC 15:0/0:0 | LPC | 0 | 0.027 | 7.119 |
| LPC 16:0;Me/0:0 (PI-104) | LPC | 0 | 0.000 | 6.695 |
| LPC 16:0;Me/0:0 \| LPC 0:0/17:0 | LPC | 0 | 0.001 | 7.040 |
| LPC 16:0/0:0 | LPC | 0 | 0.000 | 11.563 |
| LPC 16:1/0:0 | LPC | 0 | -0.061 | 7.948 |
| LPC 17:0/0:0 | LPC | 0 | 0.000 | 7.037 |
| LPC 17:1/0:0 | LPC | 0 | -0.012 | 5.624 |
| LPC 17:1/0:0 [a] (PI-104) | LPC | 0 | 0.000 | 5.546 |
| LPC 17:1/0:0 [b] | LPC | 0 | 0.009 | 3.805 |
| LPC 18:0/0:0 | LPC | 0 | -0.013 | 10.312 |
| LPC 18:1/0:0 | LPC | 0 | -0.109 | 9.783 |
| LPC 18:2/0:0 | LPC | 0 | -0.254 | 9.722 |
| LPC 18:3/0:0 | LPC | 0 | -0.001 | 5.640 |
| LPC 18:3/0:0 [a] (PI-104) | LPC | 1 | -0.016 | 5.169 |
| LPC 18:3/0:0 [b] | LPC | 0 | -0.049 | 5.414 |

| Lipid | Class | Imputed in BHS | Coefficient | Mean log-value |
|---|---|---|---|---|
| LPC 19:0/0:0 | LPC | 0 | 0.000 | 4.284 |
| LPC 19:0/0:0 [a] (PI-104) | LPC | 0 | 0.000 | 3.512 |
| LPC 19:0/0:0 [b] | LPC | 0 | -0.022 | 4.580 |
| LPC 19:1 [a] | LPC | 1 | 0.087 | 3.153 |
| LPC 19:1 [b] | LPC | 1 | 0.049 | 4.298 |
| LPC 19:1 [c] | LPC | 1 | 0.040 | 1.597 |
| LPC 20:0/0:0 | LPC | 0 | -0.072 | 4.558 |
| LPC 20:1/0:0 | LPC | 0 | 0.043 | 5.233 |
| LPC 20:2/0:0 | LPC | 0 | 0.000 | 5.178 |
| LPC 20:3/0:0 | LPC | 0 | -0.045 | 7.313 |
| LPC 20:4/0:0 | LPC | 0 | 0.000 | 8.264 |
| LPC 20:5/0:0 | LPC | 0 | 0.000 | 6.385 |
| LPC 22:0/0:0 | LPC | 0 | -0.075 | 2.949 |
| LPC 22:1/0:0 | LPC | 0 | 0.061 | 2.740 |
| LPC 22:4/0:0 | LPC | 0 | -0.037 | 4.249 |
| LPC 22:5 [n3]/0:0 [PI-104] | LPC | 1 | -0.020 | 5.550 |
| LPC 22:5 [n3]/0:0 \| LPC 22:5 [n6]/0:0 | LPC | 0 | -0.058 | 5.682 |
| LPC 22:5 [n6]/0:0 | LPC | 0 | -0.122 | 4.185 |
| LPC 22:6/0:0 | LPC | 0 | -0.012 | 6.890 |
| LPC 24:0/0:0 | LPC | 0 | 0.039 | 3.532 |
| LPC 26:0/0:0 | LPC | 0 | 0.180 | 2.839 |
| LPC O-16:0/0:0 | LPC O | 0 | 0.030 | 7.264 |
| LPC O-18:0/0:0 | LPC O | 0 | 0.003 | 6.096 |
| LPC O-18:1/0:0 | LPC O | 0 | 0.140 | 6.885 |
| LPC O-20:0/0:0 | LPC O | 0 | -0.146 | 4.449 |
| LPC O-20:1/0:0 | LPC O | 0 | -0.136 | 4.086 |
| LPC O-22:0/0:0 | LPC O | 0 | -0.078 | 4.585 |
| LPC O-22:1/0:0 | LPC O | 0 | -0.193 | 4.541 |
| LPC O-24:0/0:0 | LPC O | 0 | 0.159 | 5.390 |
| LPC O-24:1/0:0 | LPC O | 0 | 0.000 | 5.325 |
| LPC O-24:2/0:0 | LPC O | 0 | -0.068 | 3.731 |
| LPC P-16:0/0:0 | LPC P | 0 | -0.072 | 6.419 |
| LPC P-18:0/0:0 | LPC P | 0 | -0.025 | 4.604 |
| LPC P-18:1/0:0 | LPC P | 0 | -0.071 | 4.137 |
| LPC P-20:0/0:0 | LPC P | 0 | 0.008 | 1.566 |
| LPE 0:0/16:0 | LPE | 0 | 0.031 | 5.643 |
| LPE 0:0/18:0 | LPE | 0 | 0.056 | 5.934 |
| LPE 0:0/18:1 | LPE | 0 | 0.002 | 5.412 |
| LPE 0:0/18:2 | LPE | 0 | 0.013 | 5.841 |
| LPE 0:0/20:4 | LPE | 0 | -0.008 | 5.361 |
| LPE 0:0/22:6 | LPE | 0 | 0.037 | 5.277 |
| LPE 16:0/0:0 | LPE | 0 | 0.000 | 6.589 |
| LPE 18:0/0:0 | LPE | 0 | 0.000 | 6.957 |
| LPE 18:1/0:0 | LPE | 0 | 0.125 | 6.354 |
| LPE 18:2/0:0 | LPE | 0 | 0.000 | 6.660 |
| LPE 20:4/0:0 | LPE | 0 | -0.068 | 6.015 |
| LPE 22:6/0:0 | LPE | 0 | 0.000 | 5.790 |
| LPE P-16:0/0:0 | LPE P | 0 | 0.015 | 4.553 |
| LPE P-18:0/0:0 | LPE P | 0 | 0.000 | 4.971 |
| LPE P-18:1/0:0 | LPE P | 0 | 0.057 | 3.647 |
| LPE P-20:0/0:0 | LPE P | 0 | 0.060 | 2.383 |
| LPI 0:0/18:1 | LPI | 0 | 0.013 | 4.104 |
| LPI 0:0/18:2 | LPI | 0 | 0.200 | 4.351 |
| LPI 0:0/20:4 | LPI | 0 | -0.030 | 4.863 |
| LPI 18:1/0:0 | LPI | 0 | 0.101 | 5.387 |
| LPI 18:2/0:0 | LPI | 0 | 0.165 | 5.506 |
| LPI 20:4/0:0 | LPI | 0 | 0.055 | 5.828 |
| PC 14:0_16:0 | PC | 0 | 0.101 | 8.606 |
| PC 14:0_20:4 | PC | 0 | 0.047 | 7.012 |
| PC 14:0_22:6 | PC | 0 | 0.016 | 6.121 |
| PC 15:0_20:3 | PC | 0 | 0.046 | 7.083 |
| PC 15:0_20:4 | PC | 0 | -0.001 | 7.024 |
| PC 15:0_22:6 | PC | 0 | 0.000 | 6.121 |
| PC 16:0_16:0 | PC | 0 | 0.199 | 9.669 |
| PC 16:0_18:0 | PC | 0 | 0.002 | 7.904 |
| PC 16:0_18:1 | PC | 0 | 0.016 | 12.621 |
| PC 16:0_18:2 | PC | 0 | -0.326 | 12.965 |
| PC 16:0_18:3 [a] | PC | 0 | 0.000 | 9.044 |
| PC 16:0_18:3 [b] | PC | 0 | -0.006 | 8.496 |
| PC 16:0_20:3 [a] | PC | 0 | 0.000 | 11.564 |
| PC 16:0_20:3 [b] | PC | 0 | 0.054 | 8.977 |
| PC 16:0_20:4 | PC | 0 | 0.033 | 12.209 |
| PC 16:0_20:5 | PC | 0 | 0.000 | 9.950 |
| PC 16:0_22:6 | PC | 0 | -0.010 | 11.086 |
| PC 16:0;Me_18:1 | PC | 0 | 0.007 | 7.728 |
| PC 16:0;Me_18:2 | PC | 0 | 0.000 | 8.528 |
| PC 16:0;Me_20:4 | PC | 0 | -0.050 | 7.556 |
| PC 16:0;Me_22:6 | PC | 0 | -0.008 | 6.825 |
| PC 16:1_18:2 | PC | 0 | -0.121 | 8.203 |
| PC 16:1_20:4 | PC | 0 | -0.043 | 7.099 |
| PC 16:1_22:6 | PC | 0 | -0.075 | 6.508 |

| Lipid | Class | Imputed in BHS | Coefficient | Mean log-value |
|---|---|---|---|---|
| PC 17:0_18:1 | PC | 0 | 0.056 | 8.197 |
| PC 17:0_18:2 | PC | 0 | 0.048 | 8.863 |
| PC 17:0_20:4 | PC | 0 | 0.023 | 7.804 |
| PC 17:0_22:6 | PC | 0 | 0.058 | 6.635 |
| PC 17:1_18:2 | PC | 0 | 0.000 | 6.514 |
| PC 18:0_18:1 | PC | 0 | 0.000 | 10.525 |
| PC 18:0_18:2 | PC | 0 | -0.229 | 12.408 |
| PC 18:0_20:3 | PC | 0 | 0.000 | 10.263 |
| PC 18:0_20:4 | PC | 0 | 0.000 | 11.405 |
| PC 18:0_22:4 | PC | 0 | 0.000 | 7.348 |
| PC 18:0_22:5(n3) \| PC 20:1_20:4 | PC | 0 | 0.000 | 8.885 |
| PC 18:0_22:5(n6) | PC | 0 | -0.046 | 7.255 |
| PC 18:0_22:6 | PC | 0 | 0.026 | 9.886 |
| PC 18:1_18:1 | PC | 0 | 0.078 | 9.721 |
| PC 18:1_18:2 | PC | 0 | -0.013 | 10.741 |
| PC 18:1_20:3 | PC | 0 | -0.007 | 8.690 |
| PC 18:1_22:6 [a] | PC | 0 | 0.049 | 8.220 |
| PC 18:1_22:6 [b] | PC | 0 | 0.044 | 7.270 |
| PC 18:2_18:2 | PC | 0 | -0.159 | 9.847 |
| PC 18:2_20:5 | PC | 0 | -0.043 | 6.110 |
| PC 20:0_20:4 | PC | 0 | 0.036 | 5.371 |
| PC 28:0 | PC | 0 | 0.053 | 6.049 |
| PC 31:0 [a] | PC | 0 | 0.013 | 6.715 |
| PC 31:0 [b] | PC | 0 | 0.093 | 6.811 |
| PC 32:1 | PC | 0 | 0.000 | 10.030 |
| PC 32:2 | PC | 0 | 0.002 | 8.185 |
| PC 33:0 [a] | PC | 0 | 0.086 | 7.004 |
| PC 33:0 [b] | PC | 0 | 0.158 | 6.064 |
| PC 33:1 | PC | 0 | 0.020 | 8.509 |
| PC 33:2 | PC | 0 | 0.000 | 8.183 |
| PC 34:5 | PC | 0 | 0.047 | 4.733 |
| PC 35:5 | PC | 0 | 0.013 | 4.703 |
| PC 36:6 [a] | PC | 0 | 0.000 | 5.043 |
| PC 38:2 | PC | 0 | 0.109 | 7.979 |
| PC 38:4 [b] | PC | 0 | -0.034 | 8.970 |
| PC 38:5 [a] | PC | 0 | -0.029 | 10.543 |
| PC 38:5 [b] | PC | 0 | 0.000 | 9.840 |
| PC 38:6 [a] | PC | 0 | -0.176 | 8.863 |
| PC 38:7 [c] | PC | 0 | -0.181 | 5.609 |
| PC 39:5 [a] | PC | 0 | 0.000 | 4.708 |
| PC 39:5 [b] | PC | 0 | 0.000 | 5.775 |
| PC 40:7 [a] | PC | 0 | -0.100 | 5.824 |
| PC 40:8 | PC | 0 | -0.127 | 6.700 |
| PC 44:12 | PC | 1 | -0.011 | 2.812 |
| PC O-16:0/16:0 | PC O | 0 | 0.143 | 7.772 |
| PC O-16:0/20:3 | PC O | 0 | 0.013 | 8.354 |
| PC O-16:0/20:4 | PC O | 0 | 0.011 | 9.414 |
| PC O-16:0/22:6 | PC O | 0 | 0.003 | 7.951 |
| PC O-18:0/18:1 | PC O | 1 | 0.000 | 6.257 |
| PC O-18:0/18:2 | PC O | 0 | 0.000 | 7.182 |
| PC O-18:0/20:4 | PC O | 0 | 0.007 | 8.421 |
| PC O-18:0/22:6 | PC O | 0 | 0.060 | 7.002 |
| PC O-18:1/18:1 | PC O | 0 | 0.000 | 6.598 |
| PC O-18:1/18:2 | PC O | 0 | 0.012 | 7.934 |
| PC O-32:1 | PC O | 0 | -0.239 | 4.611 |
| PC O-32:2 | PC O | 0 | -0.098 | 4.682 |
| PC O-34:1 | PC O | 0 | 0.101 | 8.436 |
| PC O-34:2 | PC O | 0 | 0.000 | 8.590 |
| PC O-34:4 | PC O | 0 | -0.018 | 5.187 |
| PC O-36:0 | PC O | 0 | 0.166 | 3.834 |
| PC O-36:5 | PC O | 0 | 0.000 | 7.201 |
| PC O-38:5 | PC O | 0 | 0.169 | 9.225 |
| PC O-40:5 | PC O | 0 | -0.185 | 7.060 |
| PC P-15:0/20:4 [a] | PC P | 0 | 0.000 | 3.470 |
| PC P-15:0/20:4 [b] | PC P | 0 | 0.017 | 4.333 |
| PC P-16:0/14:0 | PC P | 0 | -0.035 | 5.036 |
| PC P-16:0/16:0 | PC P | 0 | 0.000 | 7.688 |
| PC P-16:0/16:1 | PC P | 0 | -0.090 | 6.166 |
| PC P-16:0/18:0 | PC P | 0 | 0.182 | 4.477 |
| PC P-16:0/18:1 | PC P | 0 | 0.013 | 7.906 |
| PC P-16:0/18:2 | PC P | 0 | -0.255 | 8.893 |
| PC P-16:0/18:3 | PC P | 0 | 0.044 | 5.713 |
| PC P-16:0/20:4 | PC P | 0 | -0.017 | 9.103 |
| PC P-16:0/20:5 | PC P | 0 | 0.030 | 6.795 |
| PC P-16:0/22:6 | PC P | 0 | 0.000 | 7.310 |
| PC P-17:0/20:4 [a] | PC P | 0 | 0.000 | 6.773 |
| PC P-17:0/20:4 [b] | PC P | 0 | 0.026 | 5.936 |
| PC P-18:0/18:2 | PC P | 0 | -0.016 | 7.187 |
| PC P-18:0/20:4 | PC P | 0 | 0.000 | 7.491 |
| PC P-18:0/22:5 | PC P | 0 | 0.094 | 6.287 |
| PC P-18:0/22:6 | PC P | 0 | 0.042 | 5.934 |

| Lipid | Class | Imputed in BHS | Coefficient | Mean log-value |
|---|---|---|---|---|
| PC P-18:1/18:1 | PC P | 0 | 0.001 | 6.356 |
| PC P-18:1/22:6 | PC P | 0 | 0.103 | 6.049 |
| PC P-20:0/20:4 | PC P | 0 | 0.122 | 4.423 |
| PC P-35:2 [a] | PC P | 1 | 0.000 | 6.198 |
| PC P-35:2 [b] | PC P | 1 | -0.002 | 5.599 |
| PC P-36:3 | PC P | 0 | -0.023 | 7.535 |
| PC P-38:5 [a] | PC P | 0 | -0.017 | 8.529 |
| PC P-38:5 [b] | PC P | 0 | 0.000 | 6.280 |
| PE 16:0_16:0 | PE | 0 | -0.027 | 3.110 |
| PE 16:0_16:1 | PE | 0 | -0.027 | 5.151 |
| PE 16:0_18:1 | PE | 0 | 0.034 | 7.123 |
| PE 16:0_18:2 | PE | 0 | 0.039 | 7.524 |
| PE 16:0_18:3 [a] | PE | 0 | 0.042 | 4.158 |
| PE 16:0_18:3 [b] | PE | 0 | -0.009 | 3.745 |
| PE 16:0_20:3 | PE | 0 | 0.007 | 6.301 |
| PE 16:0_20:4 | PE | 0 | 0.000 | 7.712 |
| PE 16:0_20:5 | PE | 0 | 0.010 | 5.005 |
| PE 16:0_22:6 | PE | 0 | 0.000 | 8.615 |
| PE 16:0;Me_18:1 | PE | 0 | -0.012 | 2.912 |
| PE 16:0;Me_18:2 | PE | 0 | 0.031 | 3.592 |
| PE 16:0;Me_20:4 | PE | 0 | -0.097 | 3.381 |
| PE 16:0;Me_22:6 | PE | 0 | 0.000 | 3.654 |
| PE 16:1_18:2 | PE | 0 | 0.034 | 3.224 |
| PE 16:1_20:4 | PE | 0 | 0.029 | 3.232 |
| PE 17:0_18:1 | PE | 0 | 0.059 | 3.847 |
| PE 17:0_18:2 | PE | 0 | 0.000 | 4.393 |
| PE 17:0_20:4 | PE | 0 | 0.077 | 4.737 |
| PE 17:0_22:6 | PE | 0 | 0.000 | 3.804 |
| PE 18:0_18:1 | PE | 0 | 0.000 | 6.512 |
| PE 18:0_18:2 | PE | 0 | 0.000 | 8.169 |
| PE 18:0_20:3 [a] | PE | 0 | 0.000 | 6.208 |
| PE 18:0_20:3 [b] | PE | 0 | 0.049 | 3.969 |
| PE 18:0_20:4 | PE | 0 | -0.007 | 8.469 |
| PE 18:0_22:4 | PE | 0 | -0.026 | 4.364 |
| PE 18:0_22:5(n3) | PE | 0 | 0.000 | 5.856 |
| PE 18:0_22:5(n6) | PE | 0 | -0.015 | 4.821 |
| PE 18:0_22:6 | PE | 0 | 0.000 | 7.619 |
| PE 18:1_18:1 | PE | 0 | 0.000 | 5.916 |
| PE 18:1_18:2 | PE | 0 | 0.014 | 6.514 |
| PE 18:1_22:6 [a] | PE | 0 | 0.008 | 5.528 |
| PE 18:1_22:6 [b] | PE | 0 | -0.005 | 4.696 |
| PE 20:0_20:4 | PE | 1 | 0.006 | 2.277 |
| PE 36:0 | PE | 0 | -0.183 | 1.602 |
| PE 38:5 [a] | PE | 0 | 0.023 | 7.081 |
| PE 38:5 [b] | PE | 0 | -0.029 | 6.646 |
| PE O-16:0/18:2 | PE O | 0 | -0.015 | 4.689 |
| PE O-16:0/20:3 | PE O | 0 | 0.000 | 3.691 |
| PE O-16:0/20:4 | PE O | 0 | -0.088 | 6.356 |
| PE O-16:0/22:4 | PE O | 0 | -0.113 | 3.968 |
| PE O-16:0/22:6 | PE O | 0 | -0.108 | 6.320 |
| PE O-18:0/20:4 | PE O | 0 | -0.072 | 5.719 |
| PE O-18:0/22:5 | PE O | 0 | -0.013 | 4.821 |
| PE O-18:0/22:6 | PE O | 0 | -0.011 | 5.365 |
| PE O-18:1/18:2 | PE O | 0 | -0.043 | 3.974 |
| PE O-18:1/22:6 | PE O | 0 | -0.020 | 4.832 |
| PE O-34:1 | PE O | 0 | -0.099 | 4.790 |
| PE O-36:5 | PE O | 0 | 0.000 | 4.360 |
| PE O-38:5 [a] | PE O | 0 | -0.073 | 6.472 |
| PE O-38:5 [b] | PE O | 0 | -0.014 | 4.809 |
| PE P-15:0/22:6 [a] | PE P | 0 | -0.035 | 4.072 |
| PE P-15:0/22:6 [b] | PE P | 0 | -0.031 | 4.258 |
| PE P-16:0/18:1 | PE P | 0 | 0.000 | 6.347 |
| PE P-16:0/18:2 | PE P | 0 | 0.000 | 7.513 |
| PE P-16:0/18:3 | PE P | 0 | -0.081 | 3.568 |
| PE P-16:0/20:3 [a] | PE P | 0 | 0.000 | 5.577 |
| PE P-16:0/20:3 [b] | PE P | 0 | 0.053 | 4.340 |
| PE P-16:0/20:4 | PE P | 0 | 0.000 | 8.492 |
| PE P-16:0/20:5 | PE P | 0 | 0.000 | 6.411 |
| PE P-16:0/22:4 | PE P | 0 | -0.089 | 5.714 |
| PE P-16:0/22:5(n3) | PE P | 0 | -0.026 | 7.128 |
| PE P-16:0/22:5(n6) | PE P | 0 | -0.013 | 5.448 |
| PE P-16:0/22:6 | PE P | 0 | 0.000 | 8.007 |
| PE P-17:0/20:4 [a] | PE P | 0 | 0.000 | 5.769 |
| PE P-17:0/20:4 [b] | PE P | 0 | 0.094 | 5.793 |
| PE P-17:0/22:6 [a] | PE P | 0 | 0.029 | 5.338 |
| PE P-17:0/22:6 [b] | PE P | 0 | 0.000 | 5.102 |
| PE P-18:0/18:1 | PE P | 0 | 0.068 | 6.538 |
| PE P-18:0/18:2 | PE P | 0 | 0.035 | 7.754 |
| PE P-18:0/18:3 | PE P | 0 | 0.019 | 3.587 |
| PE P-18:0/20:3 [a] | PE P | 0 | 0.000 | 5.898 |
| PE P-18:0/20:3 [b] | PE P | 0 | 0.037 | 4.719 |

| Lipid | Class | Imputed in BHS | Coefficient | Mean log-value |
|---|---|---|---|---|
| PE P-18:0/20:4 | PE P | 0 | 0.000 | 8.743 |
| PE P-18:0/20:5 | PE P | 0 | 0.007 | 6.899 |
| PE P-18:0/22:4 | PE P | 0 | -0.261 | 5.053 |
| PE P-18:0/22:5(n3) | PE P | 0 | 0.027 | 6.361 |
| PE P-18:0/22:5(n6) | PE P | 0 | 0.005 | 4.700 |
| PE P-18:0/22:6 | PE P | 0 | 0.000 | 7.774 |
| PE P-18:1/18:1 [a] | PE P | 0 | 0.003 | 5.848 |
| PE P-18:1/18:1 [b] | PE P | 0 | 0.000 | 4.496 |
| PE P-18:1/18:2 [a] | PE P | 0 | 0.000 | 6.851 |
| PE P-18:1/18:2 [b] | PE P | 0 | 0.000 | 5.450 |
| PE P-18:1/18:3 | PE P | 0 | 0.000 | 3.072 |
| PE P-18:1/20:3 [a] | PE P | 0 | 0.043 | 4.786 |
| PE P-18:1/20:3 [b] | PE P | 0 | 0.000 | 4.229 |
| PE P-18:1/20:4 [a] | PE P | 0 | 0.000 | 8.098 |
| PE P-18:1/20:4 [b] | PE P | 0 | 0.018 | 6.510 |
| PE P-18:1/20:5 [a] | PE P | 0 | -0.005 | 5.756 |
| PE P-18:1/20:5 [b] | PE P | 0 | 0.054 | 4.596 |
| PE P-18:1/22:4 | PE P | 0 | 0.034 | 4.758 |
| PE P-18:1/22:5 [a] | PE P | 0 | -0.058 | 5.769 |
| PE P-18:1/22:5 [b] | PE P | 0 | -0.102 | 4.252 |
| PE P-18:1/22:6 [a] | PE P | 0 | -0.008 | 7.188 |
| PE P-18:1/22:6 [b] | PE P | 0 | 0.078 | 5.669 |
| PE P-19:0/20:4 [a] | PE P | 0 | -0.036 | 3.229 |
| PE P-19:0/20:4 [b] | PE P | 0 | -0.106 | 3.768 |
| PE P-20:0/18:1 | PE P | 0 | -0.034 | 4.786 |
| PE P-20:0/18:2 | PE P | 0 | -0.008 | 5.300 |
| PE P-20:0/20:4 | PE P | 0 | 0.000 | 5.660 |
| PE P-20:0/22:6 | PE P | 0 | 0.186 | 4.953 |
| PE P-20:1/20:4 | PE P | 0 | -0.135 | 4.748 |
| PE P-20:1/22:6 | PE P | 0 | 0.000 | 4.322 |
| PG 34:1 | PG | 0 | -0.053 | 5.066 |
| PG 36:1 | PG | 0 | 0.000 | 4.976 |
| PG 36:2 | PG | 0 | 0.032 | 5.515 |
| PI 16:0_16:1 | PI | 0 | 0.000 | 6.340 |
| PI 16:0_20:3 [a] | PI | 0 | -0.008 | 6.813 |
| PI 16:0_20:3 [b] | PI | 0 | 0.000 | 5.370 |
| PI 16:0_20:4 | PI | 0 | 0.097 | 7.939 |
| PI 16:0;Me_18:1 \| PI 17:0_18:1 | PI | 0 | -0.101 | 5.739 |
| PI 16:0;Me_18:2 \| PI 17:0_18:2 | PI | 0 | 0.000 | 5.184 |
| PI 16:0;Me_20:4 \| PI 17:0_20:4 | PI | 0 | 0.032 | 5.766 |
| PI 16:0/16:0 | PI | 0 | -0.060 | 5.331 |
| PI 18:0_18:1 | PI | 0 | -0.150 | 8.367 |
| PI 18:0_20:2 | PI | 0 | -0.089 | 5.957 |
| PI 18:0_20:3 [a] | PI | 0 | -0.014 | 9.351 |
| PI 18:0_20:3 [b] | PI | 0 | 0.000 | 7.173 |
| PI 18:0_20:4 | PI | 0 | -0.040 | 10.383 |
| PI 18:0_22:4 | PI | 0 | -0.166 | 6.003 |
| PI 18:0_22:5 [n3] | PI | 0 | -0.130 | 7.075 |
| PI 18:0_22:5 [n6] | PI | 0 | 0.048 | 6.270 |
| PI 18:0_22:6 | PI | 0 | -0.027 | 7.420 |
| PI 18:1_18:2 | PI | 0 | 0.094 | 6.375 |
| PI 20:0_20:4 | PI | 0 | 0.020 | 4.123 |
| PI 34:0 | PI | 0 | -0.058 | 5.435 |
| PI 34:1 | PI | 0 | -0.060 | 8.278 |
| PI 36:2 | PI | 0 | -0.090 | 9.197 |
| PI 37:6 | PI | 0 | -0.052 | 3.577 |
| PI 38:5 [a] | PI | 0 | 0.017 | 6.803 |
| PI 38:5 [b] | PI | 0 | 0.095 | 6.957 |
| PI 38:6 | PI | 0 | 0.057 | 6.193 |
| PI 39:6 | PI | 0 | -0.037 | 3.358 |
| PS 36:1 | PS | 0 | 0.000 | 4.911 |
| PS 36:2 | PS | 0 | -0.105 | 3.684 |
| PS 38:3 | PS | 0 | -0.073 | 4.175 |
| PS 38:4 | PS | 0 | -0.031 | 5.246 |
| PS 38:5 | PS | 0 | -0.009 | 3.142 |
| PS 40:5 | PS | 0 | -0.045 | 4.244 |
| PS 40:6 | PS | 0 | 0.000 | 4.021 |
| SHexCer 18:1;O2/16:0 | SHexCer | 0 | 0.000 | 4.932 |
| SHexCer 18:1;O2/16:0;O | SHexCer | 0 | 0.000 | 5.192 |
| SM 16:1;O2/19:0 | SM | 0 | 0.050 | 6.517 |
| SM 16:1;O2/23:0 \| SM 17:1;O2/22:0 | SM | 0 | 0.000 | 8.396 |
| SM 16:1;O2/24:1 | SM | 0 | 0.000 | 8.698 |
| SM 17:1;O2/14:0 | SM | 0 | 0.000 | 5.214 |
| SM 17:1;O2/16:0 | SM | 0 | 0.063 | 8.665 |
| SM 17:1;O2/24:1 | SM | 0 | 0.156 | 8.296 |
| SM 18:0;O2/14:0 | SM | 0 | 0.033 | 5.602 |
| SM 18:0;O2/16:0 | SM | 0 | 0.010 | 8.617 |
| SM 18:0;O2/22:0 | SM | 0 | -0.191 | 6.056 |
| SM 18:1;O2/14:0 \| SM 16:1;O2/16:0 | SM | 0 | 0.031 | 8.988 |
| SM 18:1;O2/16:0 | SM | 0 | 0.000 | 11.703 |
| SM 18:1;O2/17:0 \| SM 17:1;O2/18:0 | SM | 0 | 0.000 | 7.946 |

| Lipid | Class | Imputed in BHS | Coefficient | Mean log-value |
|---|---|---|---|---|
| SM 18:1;O2/18:0 \| SM 16:1;O2/20:0 | SM | 0 | -0.018 | 9.944 |
| SM 18:1;O2/20:0 \| SM 16:1;O2/22:0 | SM | 0 | -0.019 | 9.392 |
| SM 18:1;O2/22:0 \| SM 16:1;O2/24:0 | SM | 0 | -0.097 | 10.163 |
| SM 18:1;O2/23:0 \| SM 17:1;O2/24:0 | SM | 0 | -0.001 | 9.232 |
| SM 18:1;O2/24:0 | SM | 0 | -0.239 | 9.717 |
| SM 18:1;O2/24:1 | SM | 0 | 0.190 | 10.472 |
| SM 18:2;O2/14:0 | SM | 0 | -0.007 | 6.411 |
| SM 18:2;O2/16:0 | SM | 0 | 0.000 | 9.407 |
| SM 18:2;O2/17:0 | SM | 0 | 0.000 | 6.064 |
| SM 18:2;O2/18:1 | SM | 0 | -0.121 | 6.457 |
| SM 18:2;O2/20:0 | SM | 0 | 0.034 | 8.721 |
| SM 18:2;O2/22:0 | SM | 0 | -0.006 | 9.477 |
| SM 18:2;O2/23:0 | SM | 0 | -0.003 | 8.565 |
| SM 18:2;O2/24:0 | SM | 0 | -0.039 | 9.160 |
| SM 19:1;O2/24:1 | SM | 0 | 0.017 | 7.331 |
| SM 34:3;O2 | SM | 0 | -0.043 | 4.328 |
| SM 35:2;O2 [b] | SM | 0 | 0.000 | 4.005 |
| SM 37:1;O2 | SM | 1 | 0.171 | 7.407 |
| SM 37:2;O2 | SM | 0 | -0.110 | 5.774 |
| SM 40:3;O2 [a] | SM | 1 | -0.040 | 7.067 |
| SM 40:3;O2 [b] | SM | 1 | -0.058 | 7.312 |
| SM 41:0;O2 | SM | 0 | -0.226 | 5.450 |
| SM 41:1;O2 [a] | SM | 0 | -0.020 | 6.862 |
| SM 43:1;O2 | SM | 0 | -0.062 | 7.121 |
| SM 43:2;O2 [b] | SM | 0 | 0.127 | 6.585 |
| SM 43:2;O2 [c] | SM | 0 | 0.004 | 5.943 |
| SM 44:1;O2 | SM | 1 | -0.012 | 4.613 |
| SM 44:2;O2 | SM | 1 | 0.082 | 5.690 |
| SM 44:3;O2 | SM | 1 | 0.020 | 5.120 |
| SPB 18:1;O2 | Sph | 1 | 0.012 | 3.986 |
| SPBP 16:1;O2 | S1P | 1 | -0.074 | 4.232 |
| SPBP 18:0;O2 | S1P | 1 | 0.144 | 4.473 |
| SPBP 18:1;O2 | S1P | 1 | -0.066 | 6.813 |
| SPBP 18:2;O2 | S1P | 1 | -0.142 | 5.243 |
| TG 48:0 | TG [SIM] | 1 | -0.026 | 9.998 |
| TG 48:0 [NL-16:0] | TG [NL] | 0 | 0.000 | 9.725 |
| TG 48:0 [NL-18:0] | TG [NL] | 1 | 0.000 | 7.350 |
| TG 48:1 | TG [SIM] | 1 | 0.000 | 11.238 |
| TG 48:1 [NL-16:1] | TG [NL] | 1 | -0.013 | 9.049 |
| TG 48:1 [NL-18:1] | TG [NL] | 0 | 0.000 | 9.945 |
| TG 48:2 | TG [SIM] | 1 | 0.000 | 10.902 |
| TG 48:2 [NL-14:0] | TG [NL] | 1 | 0.000 | 9.290 |
| TG 48:2 [NL-14:1] | TG [NL] | 0 | -0.015 | 7.992 |
| TG 48:2 [NL-16:1] | TG [NL] | 0 | 0.000 | 9.477 |
| TG 48:2 [NL-18:2] | TG [NL] | 1 | 0.020 | 8.877 |
| TG 48:3 | TG [SIM] | 1 | 0.007 | 9.647 |
| TG 48:3 [NL-14:0] | TG [NL] | 1 | 0.000 | 7.658 |
| TG 48:3 [NL-16:1] | TG [NL] | 0 | -0.013 | 8.107 |
| TG 48:3 [NL-18:3] | TG [NL] | 1 | 0.014 | 6.790 |
| TG 49:1 | TG [SIM] | 1 | 0.000 | 9.797 |
| TG 49:1 [NL-16:1] | TG [NL] | 1 | 0.029 | 6.235 |
| TG 49:1 [NL-17:1] | TG [NL] | 1 | 0.000 | 6.961 |
| TG 50:0 | TG [SIM] | 1 | -0.028 | 9.567 |
| TG 50:0 [NL-18:0] | TG [NL] | 0 | -0.017 | 8.693 |
| TG 50:1 | TG [SIM] | 1 | -0.052 | 12.099 |
| TG 50:1 [NL-14:0] | TG [NL] | 0 | 0.007 | 7.260 |
| TG 50:1 [NL-16:0] | TG [NL] | 1 | 0.000 | 11.654 |
| TG 50:1 [NL-18:1] | TG [NL] | 0 | 0.000 | 11.064 |
| TG 50:2 | TG [SIM] | 1 | -0.070 | 12.467 |
| TG 50:2 [NL-14:0] | TG [NL] | 1 | 0.097 | 9.516 |
| TG 50:2 [NL-16:1] | TG [NL] | 1 | 0.000 | 10.690 |
| TG 50:2 [NL-18:1] | TG [NL] | 0 | 0.048 | 11.343 |
| TG 50:2 [NL-18:2] | TG [NL] | 0 | 0.000 | 9.931 |
| TG 50:3 | TG [SIM] | 1 | -0.026 | 11.754 |
| TG 50:3 [NL-14:0] | TG [NL] | 1 | 0.046 | 9.392 |
| TG 50:3 [NL-14:1] | TG [NL] | 0 | -0.016 | 6.991 |
| TG 50:3 [NL-16:1] | TG [NL] | 0 | -0.005 | 10.218 |
| TG 50:3 [NL-18:2] | TG [NL] | 1 | 0.030 | 10.364 |
| TG 50:3 [NL-18:3] | TG [NL] | 1 | 0.000 | 7.985 |
| TG 50:4 | TG [SIM] | 1 | 0.000 | 10.169 |
| TG 50:4 [NL-14:0] | TG [NL] | 0 | 0.018 | 8.023 |
| TG 50:4 [NL-18:3] | TG [NL] | 1 | 0.000 | 8.341 |
| TG 50:4 [NL-20:4] | TG [NL] | 1 | 0.000 | 6.421 |
| TG 51:0 [NL-16:0] | TG [NL] | 0 | 0.000 | 6.033 |
| TG 51:1 | TG [SIM] | 1 | 0.000 | 9.556 |
| TG 51:1 [NL-17:0] | TG [NL] | 1 | 0.000 | 8.377 |
| TG 51:2 | TG [SIM] | 1 | -0.018 | 10.579 |
| TG 51:2 [NL-15:0] | TG [NL] | 0 | 0.000 | 8.232 |
| TG 51:2 [NL-17:1] | TG [NL] | 1 | 0.000 | 8.781 |
| TG 52:1 | TG [SIM] | 1 | -0.003 | 11.196 |
| TG 52:1 [NL-18:0] | TG [NL] | 0 | 0.000 | 10.226 |

| Lipid | Class | Imputed in BHS | Coefficient | Mean log-value |
|---|---|---|---|---|
| TG 52:1 [NL-18:1] | TG [NL] | 1 | 0.035 | 10.290 |
| TG 52:2 | TG [SIM] | 1 | 0.000 | 13.079 |
| TG 52:2 [NL-16:0] | TG [NL] | 0 | 0.000 | 12.124 |
| TG 52:2 [NL-18:2] | TG [NL] | 1 | 0.027 | 8.678 |
| TG 52:3 | TG [SIM] | 1 | -0.056 | 12.951 |
| TG 52:3 [NL-16:1] | TG [NL] | 0 | 0.000 | 10.016 |
| TG 52:3 [NL-18:2] | TG [NL] | 0 | 0.074 | 11.717 |
| TG 52:4 | TG [SIM] | 1 | 0.002 | 12.323 |
| TG 52:4 [NL-16:1] | TG [NL] | 1 | 0.000 | 9.773 |
| TG 52:4 [NL-18:2] | TG [NL] | 1 | 0.035 | 11.067 |
| TG 52:4 [NL-18:3] | TG [NL] | 1 | 0.000 | 9.764 |
| TG 52:5 | TG [SIM] | 1 | 0.002 | 10.780 |
| TG 52:5 [NL-18:3] | TG [NL] | 1 | 0.022 | 8.992 |
| TG 52:5 [NL-20:4] | TG [NL] | 1 | -0.011 | 7.793 |
| TG 52:5 [NL-20:5] | TG [NL] | 1 | -0.014 | 6.248 |
| TG 53:2 | TG [SIM] | 1 | 0.008 | 9.670 |
| TG 53:2 [NL-17:1] | TG [NL] | 1 | 0.027 | 5.886 |
| TG 53:2 [NL-18:1] | TG [NL] | 1 | 0.014 | 9.338 |
| TG 54:0 | TG [SIM] | 1 | -0.036 | 7.059 |
| TG 54:0 [NL-18:0] | TG [NL] | 0 | 0.000 | 6.520 |
| TG 54:1 | TG [SIM] | 1 | 0.000 | 8.982 |
| TG 54:1 [NL-18:1] | TG [NL] | 0 | 0.007 | 7.732 |
| TG 54:2 | TG [SIM] | 1 | 0.014 | 11.117 |
| TG 54:2 [NL-18:0] | TG [NL] | 0 | 0.050 | 10.004 |
| TG 54:2 [NL-20:1] | TG [NL] | 1 | -0.013 | 8.310 |
| TG 54:3 | TG [SIM] | 1 | 0.087 | 11.835 |
| TG 54:3 [NL-18:1] | TG [NL] | 0 | 0.007 | 11.598 |
| TG 54:3 [NL-18:2] | TG [NL] | 1 | 0.117 | 9.251 |
| TG 54:4 | TG [SIM] | 1 | 0.025 | 11.892 |
| TG 54:4 [NL-18:2] | TG [NL] | 0 | 0.012 | 10.298 |
| TG 54:4 [NL-20:3] | TG [NL] | 1 | 0.000 | 8.834 |
| TG 54:5 | TG [SIM] | 1 | 0.047 | 11.545 |
| TG 54:5 [NL-18:3] | TG [NL] | 1 | 0.017 | 8.712 |
| TG 54:5 [NL-20:4] | TG [NL] | 1 | 0.000 | 9.175 |
| TG 54:6 | TG [SIM] | 1 | 0.000 | 10.788 |
| TG 54:6 [NL-18:3] | TG [NL] | 1 | 0.035 | 8.205 |
| TG 54:6 [NL-20:4] | TG [NL] | 1 | 0.021 | 8.630 |
| TG 54:6 [NL-20:5] | TG [NL] | 1 | -0.011 | 7.877 |
| TG 54:6 [NL-22:6] | TG [NL] | 1 | -0.029 | 7.316 |
| TG 54:7 | TG [SIM] | 1 | 0.000 | 9.526 |
| TG 54:7 [NL-20:5] | TG [NL] | 1 | -0.007 | 7.268 |
| TG 54:7 [NL-22:6] | TG [NL] | 1 | -0.017 | 7.529 |
| TG 56:6 [NL-20:4] | TG [NL] | 0 | 0.000 | 8.398 |
| TG 56:6 [NL-22:5] | TG [NL] | 1 | -0.061 | 7.341 |
| TG 56:7 | TG [SIM] | 1 | 0.000 | 10.692 |
| TG 56:7 [NL-20:4] | TG [NL] | 1 | 0.000 | 7.993 |
| TG 56:7 [NL-20:5] | TG [NL] | 1 | -0.038 | 6.921 |
| TG 56:7 [NL-22:5] | TG [NL] | 1 | 0.012 | 6.563 |
| TG 56:7 [NL-22:6] | TG [NL] | 1 | -0.021 | 8.995 |
| TG 56:8 | TG [SIM] | 1 | -0.015 | 9.710 |
| TG 56:8 [NL-20:4] | TG [NL] | 0 | 0.040 | 7.037 |
| TG 56:8 [NL-20:5] | TG [NL] | 1 | 0.000 | 6.648 |
| TG 56:8 [NL-22:6] | TG [NL] | 1 | 0.000 | 8.334 |
| TG 56:9 | TG [SIM] | 1 | 0.000 | 8.352 |
| TG 56:9 [NL-22:6] | TG [NL] | 1 | 0.000 | 6.418 |
| TG 58:10 | TG [SIM] | 1 | 0.001 | 7.910 |
| TG 58:10 [NL-22:6] | TG [NL] | 1 | 0.005 | 6.170 |
| TG 58:8 | TG [SIM] | 1 | -0.003 | 9.311 |
| TG 58:8 [NL-22:6] | TG [NL] | 0 | 0.000 | 7.506 |
| TG 58:9 | TG [SIM] | 1 | -0.008 | 8.627 |
| TG 58:9 [NL-22:6] | TG [NL] | 1 | 0.000 | 7.044 |
| TG O-50:1 [NL-15:0] | TG O [NL] | 1 | 0.021 | 3.806 |
| TG O-50:1 [NL-16:0] | TG O [NL] | 0 | 0.000 | 5.865 |
| TG O-50:1 [NL-17:1] | TG O [NL] | 1 | 0.107 | 3.108 |
| TG O-50:1 [NL-18:1] | TG O [NL] | 1 | -0.028 | 5.248 |
| TG O-50:2 [NL-16:1] | TG O [NL] | 1 | -0.200 | 3.635 |
| TG O-50:2 [NL-18:1] | TG O [NL] | 1 | 0.119 | 3.533 |
| TG O-52:0 [NL-16:0] | TG O [NL] | 0 | 0.049 | 3.598 |
| TG O-52:1 [NL-16:0] | TG O [NL] | 1 | -0.002 | 5.205 |
| TG O-52:1 [NL-18:1] | TG O [NL] | 1 | 0.000 | 4.963 |
| TG O-52:2 [NL-16:0] | TG O [NL] | 0 | -0.075 | 5.358 |
| TG O-52:2 [NL-17:1] | TG O [NL] | 1 | -0.017 | 4.015 |
| TG O-52:2 [NL-18:1] | TG O [NL] | 1 | 0.000 | 5.622 |
| TG O-54:2 [NL-18:1] | TG O [NL] | 1 | 0.000 | 4.766 |
| TG O-54:3 [NL-17:1] | TG O [NL] | 1 | 0.119 | 3.058 |
| TG O-54:3 [NL-18:1] | TG O [NL] | 1 | 0.000 | 4.367 |
| TG O-54:4 [NL-17:1] | TG O [NL] | 1 | 0.112 | 3.403 |
| Ubiquinone | CoQ10 | 0 | 0.002 | 6.808 |

AC, acylcarnitine; CE, cholesteryl ester; Cer, ceramide; deoxyCer, deoxyceramide; dhCer, dihydroceramide; COH, free cholesterol; DE, dehydrocholesteryl ester; DG, diacylglycerol; GM1, GM (ganglioside monosialic) 1 ganglioside; GM3, GM3 ganglioside; HexCer, monohexosylceramide; Hex2Cer, dihexosylceramide; Hex3Cer, trihexosylceramide; LPC, lysophosphatidylcholine; LPC O, lysoalkylphosphatidylcholine; LPC P, Lysoalkenylphosphatidylcholine; LPE, lysophosphatidylethanolamine; LPE P, Lysoalkenylphosphatidylethanolamine; LPI, lysophosphatidylinositol; PC, phosphatidylcholine; PC O, alkylphosphatidylcholine; PC P, alkenylphosphatidylcholine; PE, phosphatidylethanolamine; PE O, alkylphosphatidylethanolamine; PE P, alkenylphosphatidylethanolamine; PG, phosphatidylglycerol; PI, phosphatidylinositol; PS, phosphatidylserine; SHexCer, sulfatide; SM, sphingomyelin; Sph, sphingosine; S1P, sphingosine-1-phosphate; TG, triacylglycerol; TG O, alkyldiacylglycerol; CoQ10, ubiquinone; [SIM], single ion monitoring; [NL], neutral loss.

**Table S5. Incremental predictive value of coronary artery and cerebrovascular disease polygenic scores beyond AusCVDRisk and L.CVDRisk.**

| PGS contributor | PGS + offset(AusCVDRisk) | | | PGS + offset(L.CVDRisk) | | | Trait reported | Publication | Sample size | Variant number | Genome build |
|---|---|---|---|---|---|---|---|---|---|---|---|
| | p value[a] | p BH | ΔC | p value[a] | p BH | ΔC | | | | | |
| PGS001839 | 0.00066 | 0.022 | 0.0045 | 0.00067 | 0.017 | 0.0042 | Coronary atherosclerosis | Priv_2022 | 391,124 | 25425 | GRCh37 |
| PGS002048 | 0.00132 | 0.022 | 0.0036 | 0.00134 | 0.022 | 0.0033 | Coronary atherosclerosis | Priv_2022 | 391,124 | 762124 | GRCh37 |
| PGS003446 | 0.00919 | 0.066 | 0.0031 | 0.00635 | 0.045 | 0.0033 | Coronary Artery disease | Tcheandjieu_2022 | 959,564 | 538084 | GRCh37 |
| PGS002724 | 0.01264 | 0.079 | 0.0030 | 0.00728 | 0.045 | 0.0033 | Ischemic stroke | Mishra_2022 | 12,788,906 | 1213574 | GRCh37 |
| PGS003356 | 0.00104 | 0.022 | 0.0029 | 0.00047 | 0.017 | 0.0033 | Coronary artery disease | Aragam_2022 | 1,165,690 | 2324683 | GRCh37 |
| PGS003726 | 0.00210 | 0.026 | 0.0026 | 0.00178 | 0.022 | 0.0029 | Coronary artery disease | Patel_2023 | 1,443,386 | 1296172 | hg19 |
| PGS003725 | 0.00300 | 0.030 | 0.0025 | 0.00228 | 0.023 | 0.0024 | Coronary artery disease | Patel_2023 | 1,443,386 | 1296172 | hg19 |
| PGS002809 | 0.07922 | 0.165 | 0.0025 | 0.06153 | 0.140 | 0.0023 | Coronary artery disease | Ahmed_2022 | 2,202,086 | 205 | NR |
| PGS001798 | 0.01854 | 0.079 | 0.0024 | 0.01584 | 0.071 | 0.0018 | Stroke | Wang_2023 | 1,370,901 | 884168 | GRCh37 |
| PGS000798 | 0.00465 | 0.039 | 0.0022 | 0.00360 | 0.030 | 0.0011 | Coronary heart disease | Severance_2019 | 547,261 | 157 | hg19 |
| PGS001793 | 0.02869 | 0.100 | 0.0022 | 0.02054 | 0.073 | 0.0018 | Stroke | Wang_2023 | 939,362 | 910099 | GRCh37 |
| PGS003438 | 0.01493 | 0.079 | 0.0021 | 0.01104 | 0.061 | 0.0021 | Coronary artery disease | Marston_2023 | 1,165,690 | 241 | NR |
| PGS000665 | 0.14124 | 0.214 | 0.0020 | 0.14892 | 0.213 | 0.0019 | Ischemic stroke | Marston_2020 | 524,354 | 32 | NR |
| PGS000899 | 0.01886 | 0.079 | 0.0019 | 0.01754 | 0.071 | 0.0014 | Coronary heart disease | Feitosa_2021 | 1,716,874 | 176 | GRCh37 |
| PGS000058 | 0.05708 | 0.137 | 0.0017 | 0.06061 | 0.140 | 0.0017 | Coronary artery disease | Morieri_2018 | 1,472,359 | 204 | hg19 |
| PGS000329 | 0.03191 | 0.100 | 0.0014 | 0.02836 | 0.084 | 0.0018 | Coronary heart disease | Mars_2020 | 408,458 | 6423165 | hg19 |
| PGS000018 | 0.02250 | 0.087 | 0.0014 | 0.01859 | 0.071 | 0.0020 | Coronary artery disease | Inouye_2018 | 382,026 | 1745179 | hg19 |
| PGS000038 | 0.28633 | 0.358 | 0.0012 | 0.24708 | 0.309 | 0.0013 | Stroke | Rutten_2018 | 446,696 | 90 | NR |
| PGS000012 | 0.07414 | 0.161 | 0.0011 | 0.07571 | 0.158 | 0.0012 | Coronary artery disease | Abraham_2016 | 194,427 | 49310 | hg19 |
| PGS001316 | 0.05739 | 0.137 | 0.0010 | 0.06143 | 0.140 | 0.0011 | Myocardial infarction (algorithmically-defined) | Tanigawa_2022 | .. | 1831 | GRCh37 |
| PGS001315 | 0.06441 | 0.146 | 0.0008 | 0.06737 | 0.146 | 0.0009 | Myocardial infarction | Tanigawa_2022 | .. | 1788 | GRCh37 |
| PGS000011 | 0.13189 | 0.214 | 0.0008 | 0.11322 | 0.191 | 0.0008 | Coronary artery disease | Tada_2015 | 365,042 | 50 | NR |
| PGS000013 | 0.01795 | 0.079 | 0.0007 | 0.01625 | 0.071 | 0.0014 | Coronary artery disease | Khera_2018 | 187,599 | 6630150 | hg19 |
| PGS002776 | 0.03599 | 0.100 | 0.0007 | 0.02808 | 0.084 | 0.0011 | Incident coronary artery disease | Wong_2022 | 187,599 | 390782 | hg19 |
| PGS000818 | 0.09497 | 0.190 | 0.0006 | 0.08813 | 0.169 | 0.0006 | Coronary heart disease | Bauer_2021 | 1,022,858 | 138 | GRCh37 |
| PGS000039 | 0.19257 | 0.267 | 0.0005 | 0.12595 | 0.191 | 0.0012 | Ischaemic stroke | Abraham_2019 | 3,202,307 | 3225583 | hg19 |
| PGS002244 | 0.10775 | 0.207 | 0.0005 | 0.08533 | 0.169 | 0.0006 | Coronary artery disease | Mars_2022 | 187,599 | 6576338 | GRCh37 |
| PGS000019 | 0.50572 | 0.550 | 0.0005 | 0.51497 | 0.548 | 0.0006 | Coronary artery disease | Paquette_2017 | 187,599 | 192 | NR |
| PGS002725 | 0.23081 | 0.312 | 0.0005 | 0.21798 | 0.287 | 0.0004 | Ischemic stroke | Mishra_2022 | 10,493,598 | 6010730 | GRCh37 |
| PGS000911 | 0.17975 | 0.257 | 0.0005 | 0.15639 | 0.217 | 0.0003 | Ischemic stroke | O_2021 | 446,696 | 530933 | GRCh37 |
| PGS000349 | 0.25799 | 0.331 | 0.0004 | 0.29239 | 0.348 | 0.0004 | Coronary artery disease | Pechlivanis_2020 | 589,596 | 70 | GRCh37 |
| PGS000057 | 0.50454 | 0.550 | 0.0004 | 0.50906 | 0.548 | 0.0002 | Coronary heart disease | Natarajan_2017 | 502,601 | 57 | hg19 |
| PGS002775 | 0.14022 | 0.214 | 0.0003 | 0.12220 | 0.191 | 0.0007 | Incident coronary artery disease | Wong_2022 | 187,599 | 1059 | hg19 |
| PGS002259 | 0.54298 | 0.566 | 0.0003 | 0.55316 | 0.566 | 0.0003 | Stroke | Lu_2021 | 2,442,550 | 534 | GRCh37 |
| PGS002262 | 0.11291 | 0.209 | 0.0002 | 0.14874 | 0.213 | 0.0003 | Coronary artery disease | Lu_2022 | 962,970 | 540 | GRCh37 |
| PGS000200 | 0.33225 | 0.386 | 0.0002 | 0.28755 | 0.348 | 0.0005 | Coronary heart disease | Tikkanen_2013 | 126,029 | 28 | NR |
| PGS002053 | 0.52915 | 0.563 | 0.0001 | 0.47251 | 0.525 | -0.0001 | Cerebrovascular disease | Priv_2022 | .. | 599726 | GRCh37 |
| PGS001314 | 0.12564 | 0.214 | 0.0001 | 0.12363 | 0.191 | 0.0004 | Acute myocardial infarction (time-to-event) | Tanigawa_2022 | .. | 1108 | GRCh37 |
| PGS000059 | 0.78032 | 0.780 | 0.0001 | 0.80012 | 0.800 | 0.0002 | Coronary heart disease | Hajek_2018 | 540,176 | 46 | NR |
| PGS001355 | 0.03442 | 0.100 | 0.0001 | 0.03037 | 0.084 | 0.0004 | Coronary artery disease | Ye_2021 | 184,305 | 2994055 | hg19 |
| PGS003355 | 0.03465 | 0.100 | 0.0000 | 0.02842 | 0.084 | 0.0008 | Coronary artery disease | Aragam_2022 | 187,599 | 1532758 | GRCh37 |
| PGS000749 | 0.11965 | 0.214 | -0.0001 | 0.10223 | 0.189 | 0.0004 | Coronary artery disease | Gola_2020 | 187,599 | 1056021 | hg19 |
| PGS000748 | 0.13732 | 0.214 | -0.0001 | 0.11407 | 0.191 | 0.0005 | Coronary artery disease | Gola_2020 | 187,599 | 3423987 | hg19 |
| PGS000710 | 0.14750 | 0.217 | -0.0002 | 0.11829 | 0.191 | 0.0002 | Myocardial infarction | Sinnott_2021 | .. | 183566 | hg19 |
| PGS000746 | 0.37958 | 0.431 | -0.0002 | 0.31580 | 0.367 | 0.0003 | Coronary artery disease | Gola_2020 | 187,599 | 1940 | hg19 |
| PGS000747 | 0.24910 | 0.328 | -0.0002 | 0.20198 | 0.273 | 0.0002 | Coronary artery disease | Gola_2020 | 187,599 | 375822 | hg19 |
| PGS000010 | 0.32456 | 0.386 | -0.0003 | 0.23408 | 0.300 | 0.0001 | Coronary heart disease | Mega_2015 | 86,995 | 27 | NR |
| PGS003727 | 0.56956 | 0.581 | -0.0004 | 0.55438 | 0.566 | -0.0001 | Coronary artery disease | Patel_2023 | 504,636 | 1125113 | hg19 |
| PGS000116 | 0.32043 | 0.386 | -0.0005 | 0.34664 | 0.394 | -0.0001 | Coronary Artery Disease | Elliott_2020 | 187,599 | 40079 | GRCh37 |
| PGS000296 | 0.05171 | 0.136 | -0.0005 | 0.03242 | 0.085 | 0.0001 | Coronary artery disease | Wang_2020 | 187,599 | 6630150 | hg19 |

[a]Likelihood-ratio test comparing the baseline (AusCVDRisk or L.CVDRisk) and PGS-containing model; BH, Benjamini-Hochberg corrected p value; NR, not reported

**Table S6. Regression coefficients for AusCVDRisk predictors used to model the LS.**

| Term | Estimate (95% CI) |
|---|---|
| Intercept | -0.080 (-0.108, -0.052) |
| log Age | 1.990 (1.869, 2.111) |
| Sex | 0.264 (0.229, 0.299) |
| Diabetes | 0.284 (0.215, 0.354) |
| log SBP | 0.163 (0.007, 0.318) |
| log Total/HDL cholesterol ratio | 0.958 (0.901, 1.015) |
| Smoking status | 0.113 (0.067, 0.159) |
| Blood pressure medication | 0.201 (0.142, 0.261) |
| Lipid-modifying medication | 0.190 (0.124, 0.256) |
| log Age x Sex | 0.287 (0.134, 0.441) |
| log Age x Diabetes | 0.400 (0.078, 0.723) |
| log Age x log SBP | 1.539 (0.959, 2.119) |
| log SBP x Blood pressure medication | -0.533 (-0.908, -0.159) |

**Table S7. Regression coefficients for AusCVDRisk predictors used to model the PGS.**

| Term | Estimate (95% CI) |
|---|---|
| Intercept | -0.135 (-0.184, -0.085) |
| Sex | 0.106 (0.050, 0.163) |
| Diabetes | 0.084 (-0.061, 0.228) |
| log Total/HDL cholesterol ratio | 0.275 (0.184, 0.366) |
| log SBP | -0.230 (-0.433, -0.027) |
| Smoking status | 0.067 (0.007, 0.127) |

**Table S8. Discrimination (Harrell's C-statistic) of AusCVDRisk, L.CVDRisk, G.CVDRisk, and LG.CVDRisk.**

| Data view | Dataset | AusCVDRisk | L.CVDRisk | ΔC (L)[a] | G.CVDRisk | ΔC (G)[b] | LG.CVDRisk | ΔC (LG)[c] |
|---|---|---|---|---|---|---|---|---|
| Overall (whole cohorts) | AusDiab | 0.788 (0.756-0.820) | 0.802 (0.770-0.834) | 0.014 (0.003, 0.025) | .. | .. | .. | .. |
| | BHS | 0.793 (0.759-0.824) | 0.799 (0.765-0.830) | 0.006 (-0.009, 0.021) | 0.804 (0.770-0.835) | 0.011 (-0.004, 0.025) | 0.803 (0.769-0.834) | 0.010 (-0.010, 0.029) |
| | MCCS[d] | 0.824 (0.790-0.853) | 0.835 (0.798-0.867) | 0.011 (-0.007, 0.029) | 0.831 (0.795-0.861) | 0.006 (-0.010, 0.023) | 0.831 (0.794-0.863) | 0.007 (-0.015, 0.028) |
| | ASPREE[d] | 0.701 (0.669-0.733) | 0.694 (0.659-0.726) | -0.008 (-0.025, 0.010) | 0.728 (0.694-0.758) | 0.026 (0.007, 0.046) | 0.722 (0.688-0.753) | 0.020 (-0.001, 0.042) |
| | Pooled estimates | .. | .. | 0.008 (0.001, 0.015) | .. | 0.013 (0.004, 0.023) | .. | 0.012 (0.000, 0.024) |
| Intermediate risk individuals | AusDiab | 0.583 (0.512-0.653) | 0.639 (0.569-0.709) | 0.056 (0.001, 0.111) | .. | .. | .. | .. |
| | BHS | 0.554 (0.471-0.634) | 0.614 (0.530-0.691) | 0.060 (-0.008, 0.132) | 0.653 (0.568-0.732) | 0.099 (0.036, 0.162) | 0.676 (0.594-0.751) | 0.122 (0.054, 0.191) |
| | MCCS[d] | 0.603 (0.524-0.677) | 0.661 (0.578-0.734) | 0.058 (0.001, 0.117) | 0.663 (0.581-0.735) | 0.060 (-0.009, 0.132) | 0.657 (0.571-0.734) | 0.054 (-0.016, 0.125) |
| | ASPREE[d] | 0.595 (0.536-0.651) | 0.583 (0.521-0.641) | -0.013 (-0.065, 0.04) | 0.660 (0.598-0.719) | 0.065 (0.010, 0.119) | 0.640 (0.58-0.696) | 0.045 (-0.015, 0.105) |
| | Pooled estimates | .. | .. | 0.036 (0.007, 0.065) | .. | 0.074 (0.039, 0.110) | .. | 0.071 (0.033, 0.109) |

Gain in discrimination when [a] L.CVDRisk; [b] G.CVDRisk; and [c] LG.CVDRisk was compared with AusCVDRisk.
[d] Statistics were weighted to account for case-cohort designs.

**Table S9. Risk stratification by AusCVDRisk, L.CVDRisk, G.CVDRisk, and LG.CVDRisk.**

| | | AusCVDRisk | | | | | L.CVDRisk | | | | | G.CVDRisk | | | | | LG.CVDRisk | | | | |
|---|---|---|---|---|---|---|---|---|---|---|---|---|---|---|---|---|---|---|---|---|---|
| Cohort | Risk category | Overall | Events | Non-events | Censored | Event rate | Overall | Events | Non-events | Censored | Event rate | Overall | Events | Non-events | Censored | Event rate | Overall | Events | Non-events | Censored | Event rate |
| AusDiab | Low | 4083 | 44 | 4012 | 27 | 0.011 | 4056 | 39 | 3991 | 26 | 0.010 | .. | .. | .. | .. | .. | .. | .. | .. | .. | .. |
| | Int. | 1169 | 67 | 1072 | 30 | 0.058 | 1118 | 51 | 1039 | 28 | 0.046 | .. | .. | .. | .. | .. | .. | .. | .. | .. | .. |
| | High | 421 | 45 | 350 | 26 | 0.110 | 499 | 66 | 404 | 29 | 0.136 | .. | .. | .. | .. | .. | .. | .. | .. | .. | .. |
| BHS | Low | 1353 | 27 | 1312 | 14 | 0.020 | 1285 | 18 | 1253 | 14 | 0.014 | 1362 | 27 | 1322 | 13 | 0.020 | 1308 | 24 | 1271 | 13 | 0.018 |
| | Int. | 555 | 46 | 497 | 12 | 0.084 | 570 | 49 | 513 | 8 | 0.086 | 541 | 41 | 487 | 13 | 0.077 | 527 | 37 | 481 | 9 | 0.071 |
| | High | 322 | 64 | 234 | 24 | 0.208 | 375 | 70 | 277 | 28 | 0.195 | 327 | 69 | 234 | 24 | 0.220 | 395 | 76 | 291 | 28 | 0.200 |
| MCCS | Low | 1107 | 15 | 1080 | 12 | .. | 997 | 14 | 975 | 8 | .. | 1075 | 18 | 1045 | 12 | .. | 965 | 12 | 945 | 8 | .. |
| | Int. | 619 | 59 | 540 | 20 | .. | 577 | 33 | 526 | 18 | .. | 614 | 47 | 547 | 20 | .. | 574 | 34 | 522 | 18 | .. |
| | High | 254 | 56 | 185 | 13 | .. | 406 | 83 | 304 | 19 | .. | 291 | 65 | 213 | 13 | .. | 441 | 84 | 338 | 19 | .. |
| MCCS (weighted) | Low | 5865.8 | 34.5 | 5767.2 | 64.1 | 0.006 | 5281.2 | 32.0 | 5206.5 | 42.7 | 0.006 | 5684.9 | 40.5 | 5580.3 | 64.1 | 0.007 | 5116.0 | 27.0 | 5046.3 | 42.7 | 0.005 |
| | Int. | 3125.9 | 135.5 | 2883.6 | 106.8 | 0.044 | 2978.5 | 73.5 | 2808.8 | 96.1 | 0.025 | 3137.8 | 110.0 | 2921.0 | 106.8 | 0.036 | 2959.6 | 76.0 | 2787.5 | 96.1 | 0.026 |
| | High | 1189.8 | 132.5 | 987.9 | 69.4 | 0.115 | 1921.8 | 197.0 | 1623.4 | 101.5 | 0.106 | 1358.8 | 152.0 | 1137.4 | 69.4 | 0.115 | 2105.9 | 199.5 | 1804.9 | 101.5 | 0.097 |
| ASPREE | Low | 607 | 18 | 565 | 24 | .. | 673 | 23 | 622 | 28 | .. | 618 | 18 | 582 | 18 | .. | 709 | 23 | 661 | 25 | .. |
| | Int. | 1503 | 111 | 1318 | 74 | .. | 1370 | 99 | 1212 | 59 | .. | 1382 | 89 | 1217 | 76 | .. | 1211 | 75 | 1078 | 58 | .. |
| | High | 835 | 146 | 633 | 56 | .. | 902 | 153 | 682 | 67 | .. | 945 | 168 | 717 | 60 | .. | 1025 | 177 | 777 | 71 | .. |
| ASPREE (weighted) | Low | 2005.4 | 26.4 | 1898.4 | 80.6 | 0.013 | 2217.8 | 33.8 | 2089.9 | 94.1 | 0.015 | 2042.4 | 26.4 | 1955.5 | 60.5 | 0.013 | 2338.7 | 33.8 | 2221.0 | 84.0 | 0.015 |
| | Int. | 4849.1 | 172.0 | 4428.5 | 248.6 | 0.036 | 4423.4 | 152.8 | 4072.3 | 198.2 | 0.035 | 4477.7 | 133.3 | 4089.1 | 255.4 | 0.030 | 3929.0 | 112.1 | 3622.1 | 194.9 | 0.029 |
| | High | 2548.3 | 233.3 | 2126.9 | 188.2 | 0.094 | 2761.7 | 245.1 | 2291.5 | 225.1 | 0.092 | 2882.8 | 272.1 | 2409.1 | 201.6 | 0.097 | 3135.1 | 285.8 | 2610.7 | 238.6 | 0.094 |

**Table S10. Risk reclassification tables underlying net reclassification improvement analyses.**

| | | L.CVDRisk | | | | | G.CVDRisk | | | | | LG.CVDRisk | | | | |
|---|---|---|---|---|---|---|---|---|---|---|---|---|---|---|---|---|
| Cohort | AusCVDRisk category | L.CVDRisk category | Overall | Events | Non-events | Censored | G.CVDRisk category | Overall | Events | Non-events | Censored | LG.CVDRisk category | Overall | Events | Non-events | Censored |
| AusDiab | Low | Low | 3867 | 35 | 3809 | 23 | Low | .. | .. | .. | .. | Low | .. | .. | .. | .. |
| | Low | Intermediate | 213 | 9 | 201 | 3 | Intermediate | .. | .. | .. | .. | Intermediate | .. | .. | .. | .. |
| | Low | High | 3 | 0 | 2 | 1 | High | .. | .. | .. | .. | High | .. | .. | .. | .. |
| | Intermediate | Low | 189 | 4 | 182 | 3 | Low | .. | .. | .. | .. | Low | .. | .. | .. | .. |
| | Intermediate | Intermediate | 828 | 38 | 768 | 22 | Intermediate | .. | .. | .. | .. | Intermediate | .. | .. | .. | .. |
| | Intermediate | High | 152 | 25 | 122 | 5 | High | .. | .. | .. | .. | High | .. | .. | .. | .. |
| | High | Low | 0 | 0 | 0 | 0 | Low | .. | .. | .. | .. | Low | .. | .. | .. | .. |
| | High | Intermediate | 77 | 4 | 70 | 3 | Intermediate | .. | .. | .. | .. | Intermediate | .. | .. | .. | .. |
| | High | High | 344 | 41 | 280 | 23 | High | .. | .. | .. | .. | High | .. | .. | .. | .. |
| BHS | Low | Low | 1219 | 16 | 1191 | 12 | Low | 1259 | 21 | 1226 | 12 | Low | 1201 | 18 | 1171 | 12 |
| | Low | Intermediate | 134 | 11 | 121 | 2 | Intermediate | 94 | 6 | 86 | 2 | Intermediate | 141 | 7 | 132 | 2 |
| | Low | High | 0 | 0 | 0 | 0 | High | 0 | 0 | 0 | 0 | High | 11 | 2 | 9 | 0 |
| | Intermediate | Low | 65 | 2 | 61 | 2 | Low | 102 | 5 | 96 | 1 | Low | 101 | 4 | 97 | 0 |
| | Intermediate | Intermediate | 395 | 30 | 362 | 3 | Intermediate | 380 | 28 | 343 | 9 | Intermediate | 329 | 20 | 303 | 6 |
| | Intermediate | High | 95 | 14 | 74 | 7 | High | 73 | 13 | 58 | 2 | High | 125 | 22 | 97 | 6 |
| | High | Low | 1 | 0 | 1 | 0 | Low | 1 | 1 | 0 | 0 | Low | 6 | 2 | 3 | 1 |
| | High | Intermediate | 41 | 8 | 30 | 3 | Intermediate | 67 | 7 | 58 | 2 | Intermediate | 57 | 10 | 46 | 1 |
| | High | High | 280 | 56 | 203 | 21 | High | 254 | 56 | 176 | 22 | High | 259 | 52 | 185 | 22 |
| MCCS (weighted) | Low | Low | 4963.3 | 18.5 | 4902.1 | 4963.3 | Low | 5222.8 | 27.0 | 5147.8 | 48.1 | Low | 4699.2 | 16.0 | 4640.5 | 42.7 |
| | Low | Intermediate | 894.6 | 13.5 | 859.7 | 894.6 | Intermediate | 643.0 | 7.5 | 619.4 | 16.0 | Intermediate | 1105.4 | 16.0 | 1068.0 | 21.4 |
| | Low | High | 7.8 | 2.5 | 5.3 | 7.8 | High | 0.0 | 0.0 | 0.0 | 0.0 | High | 61.2 | 2.5 | 58.7 | 0.0 |
| | Intermediate | Low | 317.9 | 13.5 | 304.4 | 317.9 | Low | 462.1 | 13.5 | 432.5 | 16.0 | Low | 411.5 | 11.0 | 400.5 | 0.0 |
| | Intermediate | Intermediate | 1931.8 | 57.5 | 1804.9 | 1931.8 | Intermediate | 2247.4 | 90.0 | 2082.6 | 74.8 | Intermediate | 1654.5 | 52.5 | 1537.9 | 64.1 |
| | Intermediate | High | 876.2 | 64.5 | 774.3 | 876.2 | High | 416.5 | 32.0 | 368.5 | 16.0 | High | 1059.9 | 72.0 | 945.2 | 42.7 |
| | High | Low | 0.0 | 0.0 | 0.0 | 0.0 | Low | 0.0 | 0.0 | 0.0 | 0.0 | Low | 5.3 | 0.0 | 5.3 | 0.0 |
| | High | Intermediate | 152.0 | 2.5 | 144.2 | 152.0 | Intermediate | 247.5 | 12.5 | 218.9 | 16.0 | Intermediate | 199.7 | 7.5 | 181.6 | 10.7 |
| | High | High | 1037.8 | 130.0 | 843.7 | 1037.8 | High | 942.4 | 120.0 | 769.0 | 53.4 | High | 984.7 | 125.0 | 801.0 | 58.7 |
| ASPREE (weighted) | Low | Low | 1586.8 | 24.4 | 1498.6 | 1586.8 | Low | 1493.2 | 14.8 | 1431.4 | 47.0 | Low | 1456.2 | 14.8 | 1387.7 | 53.8 |
| | Low | Intermediate | 418.6 | 2.0 | 399.8 | 418.6 | Intermediate | 508.9 | 11.6 | 463.7 | 33.6 | Intermediate | 525.7 | 11.6 | 487.2 | 26.9 |
| | Low | High | 0.0 | 0.0 | 0.0 | 0.0 | High | 3.4 | 0.0 | 3.4 | 0.0 | High | 23.5 | 0.0 | 23.5 | 0.0 |
| | Intermediate | Low | 627.6 | 9.4 | 588.0 | 627.6 | Low | 549.2 | 11.6 | 524.2 | 13.4 | Low | 859.0 | 19.0 | 809.8 | 30.2 |
| | Intermediate | Intermediate | 3443.6 | 110.5 | 3185.3 | 3443.6 | Intermediate | 3458.7 | 95.3 | 3185.3 | 178.1 | Intermediate | 2808.4 | 76.7 | 2604.0 | 127.7 |
| | Intermediate | High | 777.9 | 52.1 | 655.2 | 777.9 | High | 841.3 | 65.1 | 719.0 | 57.1 | High | 1181.8 | 76.3 | 1014.7 | 90.7 |
| | High | Low | 3.4 | 0.0 | 3.4 | 3.4 | Low | 0.0 | 0.0 | 0.0 | 0.0 | Low | 23.5 | 0.0 | 23.5 | 0.0 |
| | High | Intermediate | 561.2 | 40.4 | 487.2 | 561.2 | Intermediate | 510.2 | 26.4 | 440.2 | 43.7 | Intermediate | 595.0 | 23.8 | 530.9 | 40.3 |
| | High | High | 1983.8 | 193.0 | 1636.3 | 1983.8 | High | 2038.1 | 206.9 | 1686.7 | 144.5 | High | 1929.8 | 209.5 | 1572.5 | 147.8 |

**Table S11. Incremental discrimination relative to AusCVDRisk: sex-segregated pooled analysis.**

| Data view | Sex | L.CVDRisk ΔC | G.CVDRisk ΔC | LG.CVDRisk ΔC |
|---|---|---|---|---|
| Overall (whole cohorts) | Together | 0.008 (0.001, 0.015) | 0.013 (0.004, 0.023) | 0.012 (0.000, 0.024) |
| | Women | 0.010 (0.000, 0.021) | 0.003 (-0.009, 0.015) | 0.005 (-0.011, 0.021) |
| | Men | 0.003 (-0.007, 0.013) | 0.014 (-0.001, 0.029) | 0.011 (-0.008, 0.030) |
| Intermediate risk individuals | Together | 0.036 (0.007, 0.065) | 0.074 (0.039, 0.110) | 0.071 (0.033, 0.109) |
| | Women | 0.004 (-0.034, 0.041) | 0.037 (-0.006, 0.081) | 0.016 (-0.031, 0.064) |
| | Men | 0.042 (-0.001, 0.085) | 0.116 (0.051, 0.182) | 0.126 (0.061, 0.191) |

**Table S12. Net reclassification improvement relative to AusCVDRisk: sex-segregated pooled analysis.**

| Data view | Sex | Statistic | L.CVDRisk | G.CVDRisk | LG.CVDRisk |
|---|---|---|---|---|---|
| Overall (whole cohorts) | Together | NRI | 0.072 (0.033, 0.111) | 0.044 (0.000, 0.089) | 0.080 (0.031, 0.129) |
| | | NRI+ | 0.103 (0.065, 0.141) | 0.064 (0.022, 0.107) | 0.138 (0.091, 0.185) |
| | | NRI- | -0.03 (-0.036, -0.023) | -0.016 (-0.027, -0.006) | -0.069 (-0.081, -0.056) |
| | Women | NRI | 0.145 (0.082, 0.209) | 0.082 (0.010, 0.154) | 0.155 (0.071, 0.240) |
| | | NRI+ | 0.192 (0.129, 0.255) | 0.109 (0.038, 0.180) | 0.233 (0.151, 0.316) |
| | | NRI- | -0.035 (-0.043, -0.027) | -0.023 (-0.036, -0.010) | -0.078 (-0.093, -0.062) |
| | Men | NRI | 0.027 (-0.021, 0.074) | 0.029 (-0.025, 0.083) | 0.040 (-0.018, 0.099) |
| | | NRI+ | 0.046 (0.000, 0.091) | 0.032 (-0.019, 0.083) | 0.080 (0.025, 0.134) |
| | | NRI- | -0.021 (-0.032, -0.01) | -0.005 (-0.022, 0.012) | -0.050 (-0.069, -0.030) |
| Intermediate risk individuals | Together | NRI | 0.264 (0.194, 0.334) | 0.231 (0.147, 0.315) | 0.305 (0.212, 0.397) |
| | | NRI+ | 0.286 (0.219, 0.354) | 0.228 (0.147, 0.308) | 0.382 (0.293, 0.470) |
| | | NRI- | -0.014 (-0.032, 0.004) | -0.006 (-0.027, 0.016) | -0.068 (-0.093, -0.042) |
| | Women | NRI | 0.241 (0.149, 0.333) | 0.242 (0.141, 0.344) | 0.295 (0.177, 0.414) |
| | | NRI+ | 0.244 (0.155, 0.332) | 0.177 (0.080, 0.273) | 0.336 (0.224, 0.448) |
| | | NRI- | 0.025 (-0.001, 0.052) | 0.058 (0.029, 0.088) | 0.004 (-0.033, 0.041) |
| | Men | NRI | 0.303 (0.203, 0.402) | 0.237 (0.103, 0.370) | 0.313 (0.174, 0.453) |
| | | NRI+ | 0.356 (0.260, 0.453) | 0.316 (0.186, 0.447) | 0.452 (0.317, 0.587) |
| | | NRI- | -0.055 (-0.079, -0.031) | -0.072 (-0.102, -0.042) | -0.146 (-0.182, -0.111) |

**Table S13. Calibration intercepts and slopes for four risk models: sex-segregated pooled analysis.**

| Calibration | Data view | Sex | AusCVDRisk | L.CVDRisk | G.CVDRisk | LG.CVDRisk |
|---|---|---|---|---|---|---|
| **Intercept** | Overall (whole cohorts) | Together | -0.028 (-0.101, 0.045) | -0.114 (-0.187, -0.041) | 0.027 (-0.057, 0.112) | -0.073 (-0.157, 0.012) |
| | | Women | 0.000 (-0.116, 0.115) | -0.087 (-0.203, 0.028) | 0.064 (-0.068, 0.196) | -0.037 (-0.169, 0.095) |
| | | Men | -0.039 (-0.133, 0.056) | -0.125 (-0.220, -0.030) | 0.011 (-0.098, 0.121) | -0.089 (-0.198, 0.021) |
| | Intermediate risk individuals | Together | 0.011 (-0.106, 0.127) | -0.077 (-0.193, 0.040) | 0.022 (-0.112, 0.155) | -0.078 (-0.212, 0.055) |
| | | Women | 0.226 (0.071, 0.382) | 0.120 (-0.035, 0.276) | 0.259 (0.089, 0.430) | 0.145 (-0.026, 0.315) |
| | | Men | -0.176 (-0.352, 0.000) | -0.246 (-0.422, -0.070) | -0.221 (-0.437, -0.005) | -0.306 (-0.522, -0.090) |
| **Slope** | Overall (whole cohorts) | Together | 1.346 (1.230, 1.461) | 1.203 (1.102, 1.304) | 1.305 (1.175, 1.436) | 1.154 (1.038, 1.269) |
| | | Women | 1.426 (1.239, 1.613) | 1.358 (1.191, 1.525) | 1.390 (1.184, 1.595) | 1.313 (1.126, 1.501) |
| | | Men | 1.380 (1.208, 1.551) | 1.150 (1.005, 1.295) | 1.333 (1.139, 1.526) | 1.084 (0.918, 1.251) |
| | Intermediate risk individuals | Together | 1.443 (0.857, 2.029) | 0.993 (0.660, 1.325) | 1.333 (0.933, 1.732) | 1.002 (0.698, 1.306) |
| | | Women | 1.687 (0.901, 2.473) | 0.899 (0.467, 1.331) | 1.400 (0.892, 1.907) | 0.993 (0.613, 1.373) |
| | | Men | 1.306 (0.315, 2.297) | 1.123 (0.600, 1.645) | 1.358 (0.697, 2.018) | 1.043 (0.534, 1.553) |

## Supplementary Figures

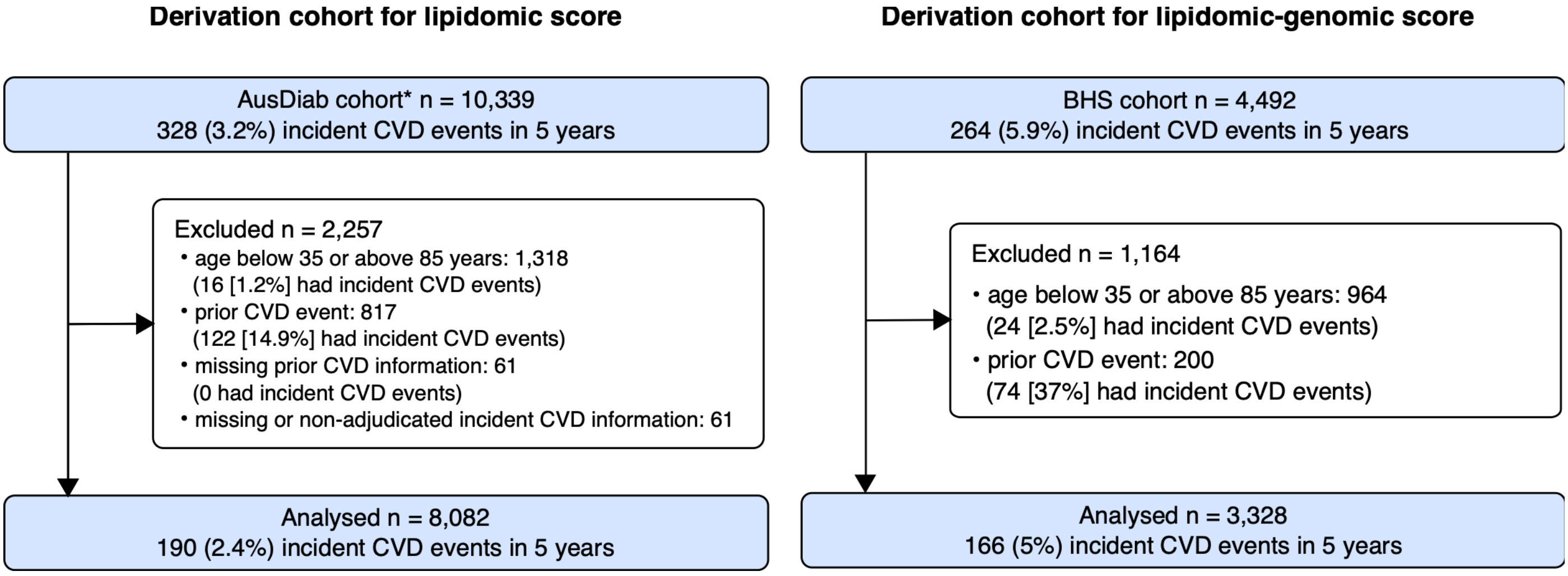


**Figure S1. Flowcharts for derivation cohorts (ages 35-85). Participant selection and exclusion for AusDiab and BHS.**

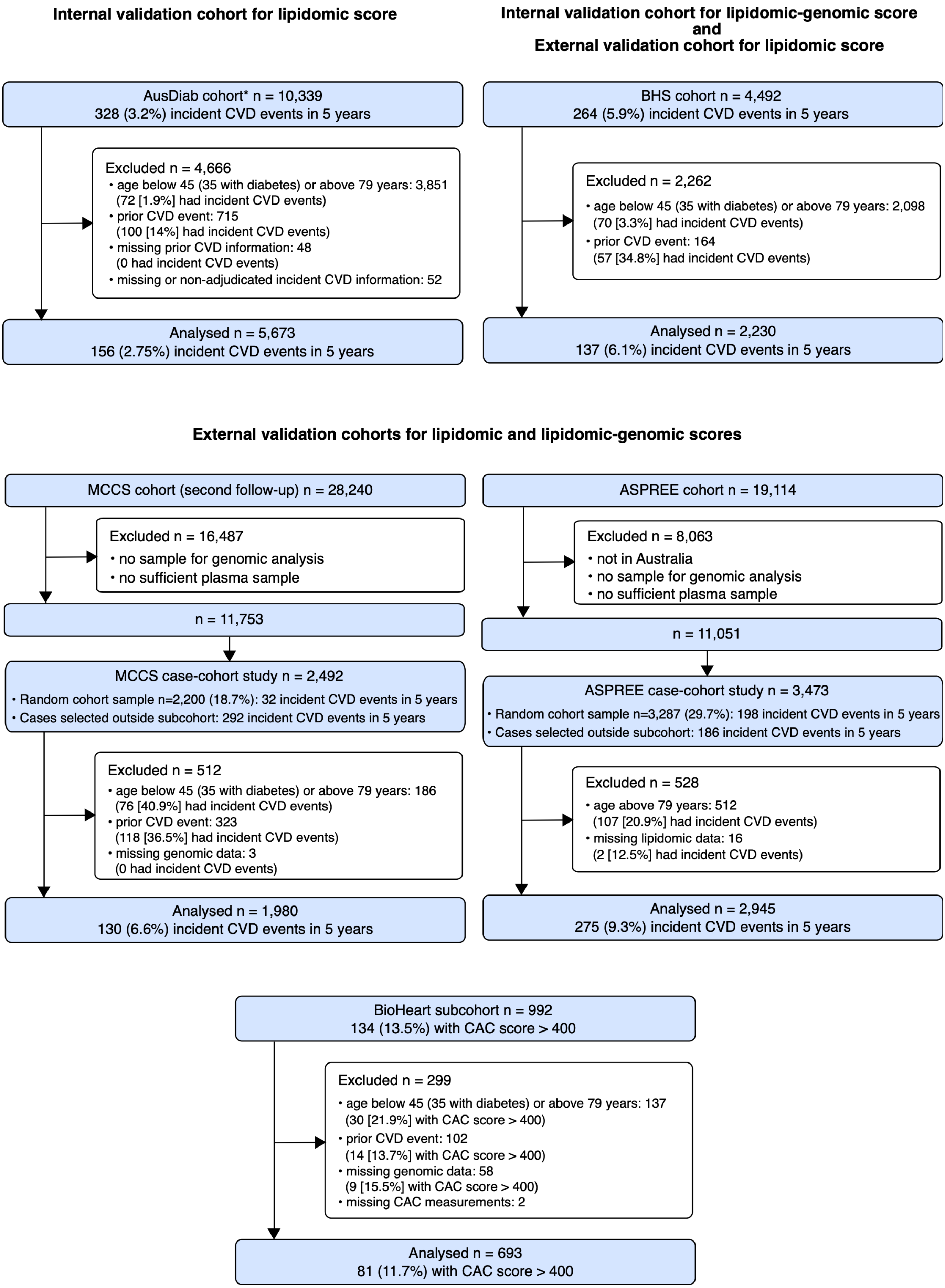


**Figure S2. Flowcharts for internal and external validation cohorts (ages 45-79, or 35-79 for participants with diabetes). Participant selection and exclusion for AusDiab, BHS, MCCS, ASPREE, and BioHEART.**

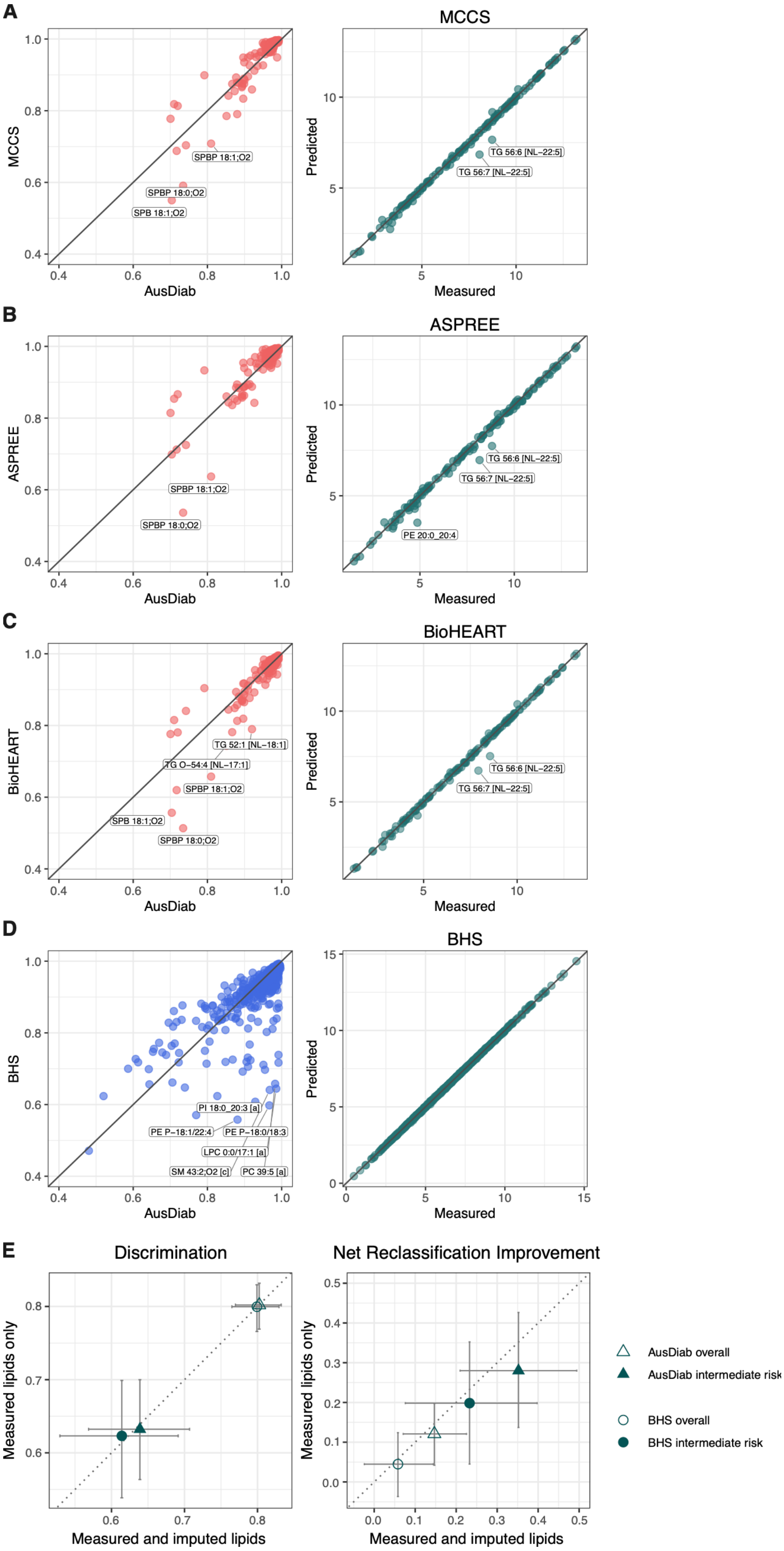


**Figure S3. Evaluation of lipid imputation performance.**

Imputation models were developed in the AusDiab cohort. A total of 139 lipid species not measured in BHS were subsequently imputed in that cohort. Panels A–C evaluate imputation performance for the 139 lipids in MCCS, ASPREE, and BioHEART, respectively. Because these lipids were not measured in BHS, direct validation was not possible; therefore, Panel D evaluates imputation performance for the 550 lipid species measured in BHS. In panels A–D, the left scatter plot compares correlations between measured and imputed lipid concentrations in the derivation cohort (AusDiab; x-axis) and the corresponding validation cohort (y-axis). The right scatter plot compares mean measured and imputed lipid concentrations in the validation cohort. Panel E presents a sensitivity analysis comparing predictive discrimination (Harrell’s C-statistic) and net reclassification improvement of L.CVDRisk models developed with and without the 139 imputed lipid species.

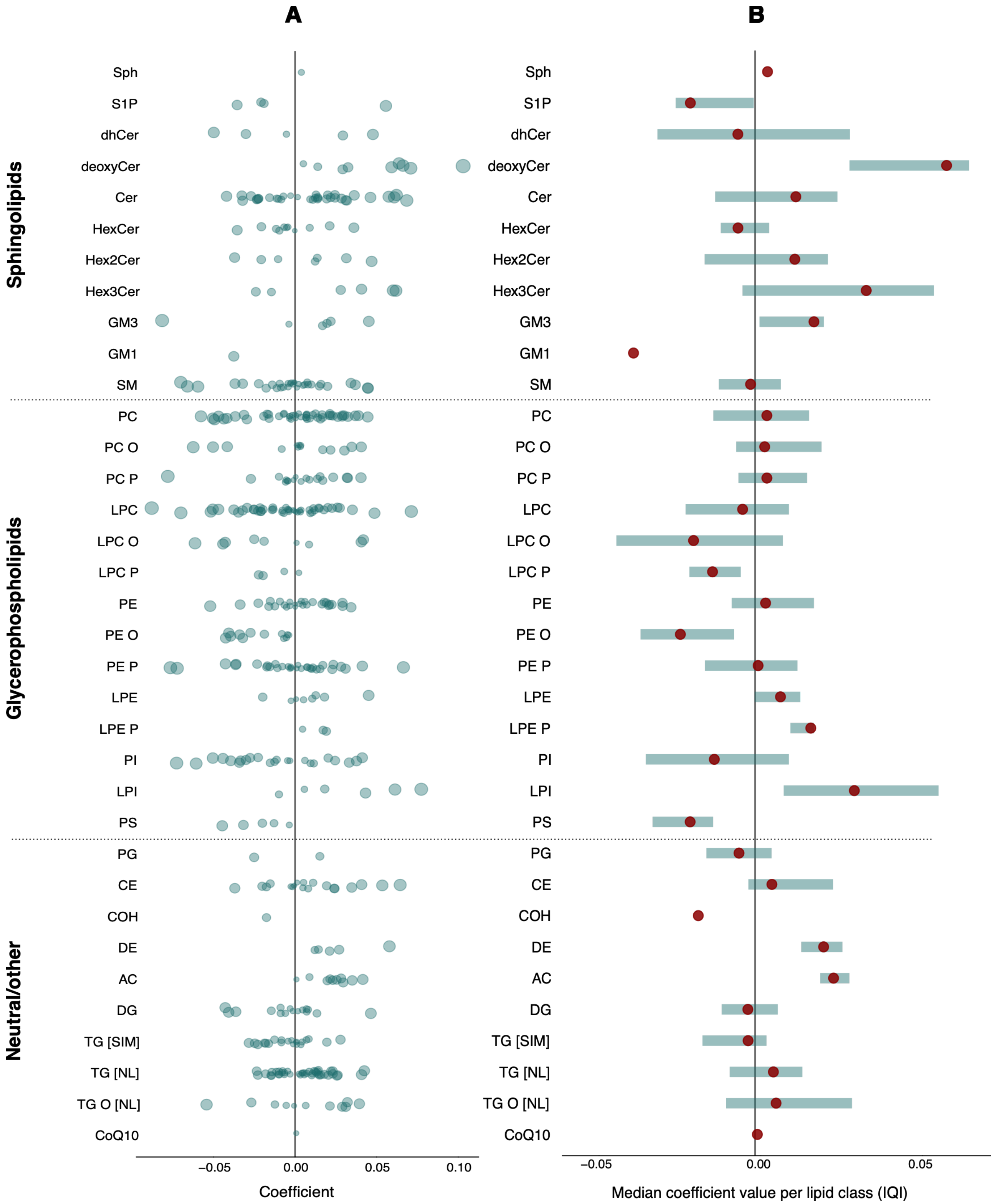


**Figure S4. Standardised coefficients from the LS regularised Cox regression model.**

Panel A shows coefficients for the 512 lipid species retained in the model, grouped by lipid class. Panel B summarises the distribution of coefficients within each lipid class, showing the median coefficient and interquartile interval.

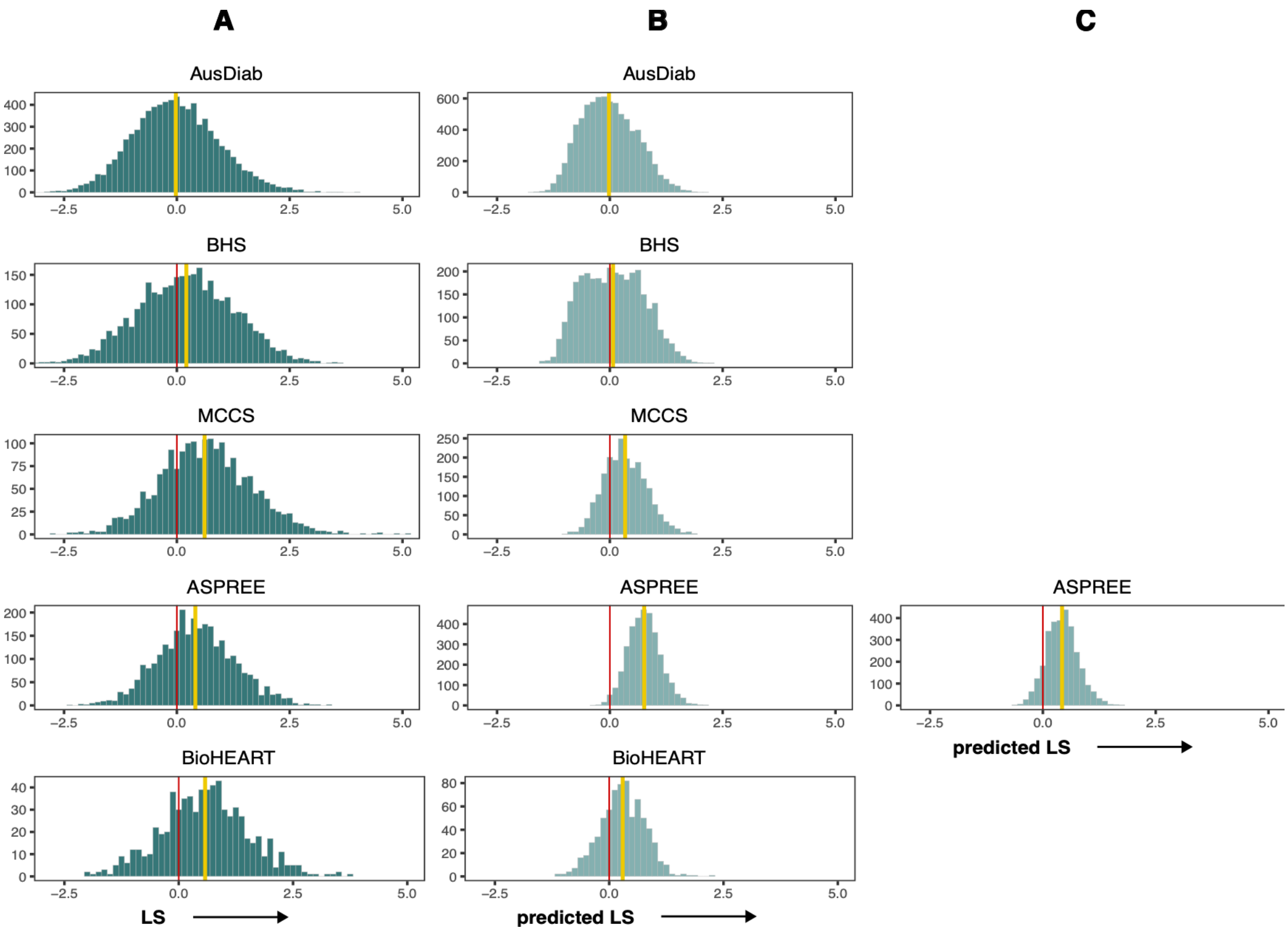


**Figure S5. Distribution of observed and predicted lipidomic scores (LS) across five cohorts.**

The right-shift of predicted LS relative to observed LS in ASPREE motivated cohort-level centring of predicted LS values prior to calculation of residualised LS. Panel **A**: observed LS; Panel **B**: LS predicted from AusCVDRisk predictors; Panel **C**: LS predicted from AusCVDRisk predictors after cohort-level centring in ASPREE.

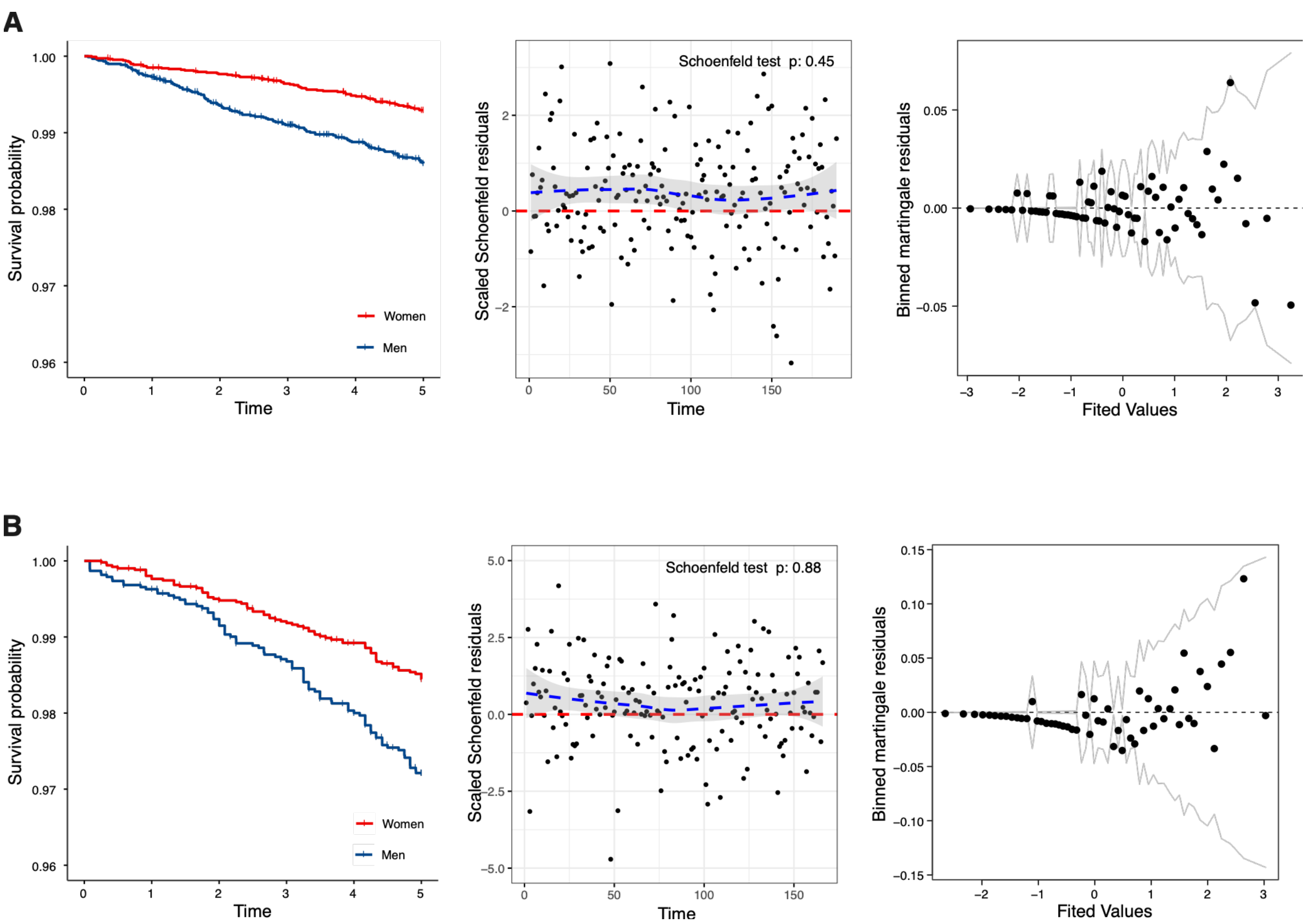


**Figure S6. Diagnostics for Cox proportional hazard regression models.**

Assessment of sex-stratified baseline survival functions, proportional hazard assumption (scaled Schoenfeld residuals), and overall fit (binned Martingale residuals) for (**A**) proto-L.CVDRisk model (AusDiab study), (**B**) proto-G.CVDRisk model (BHS study).

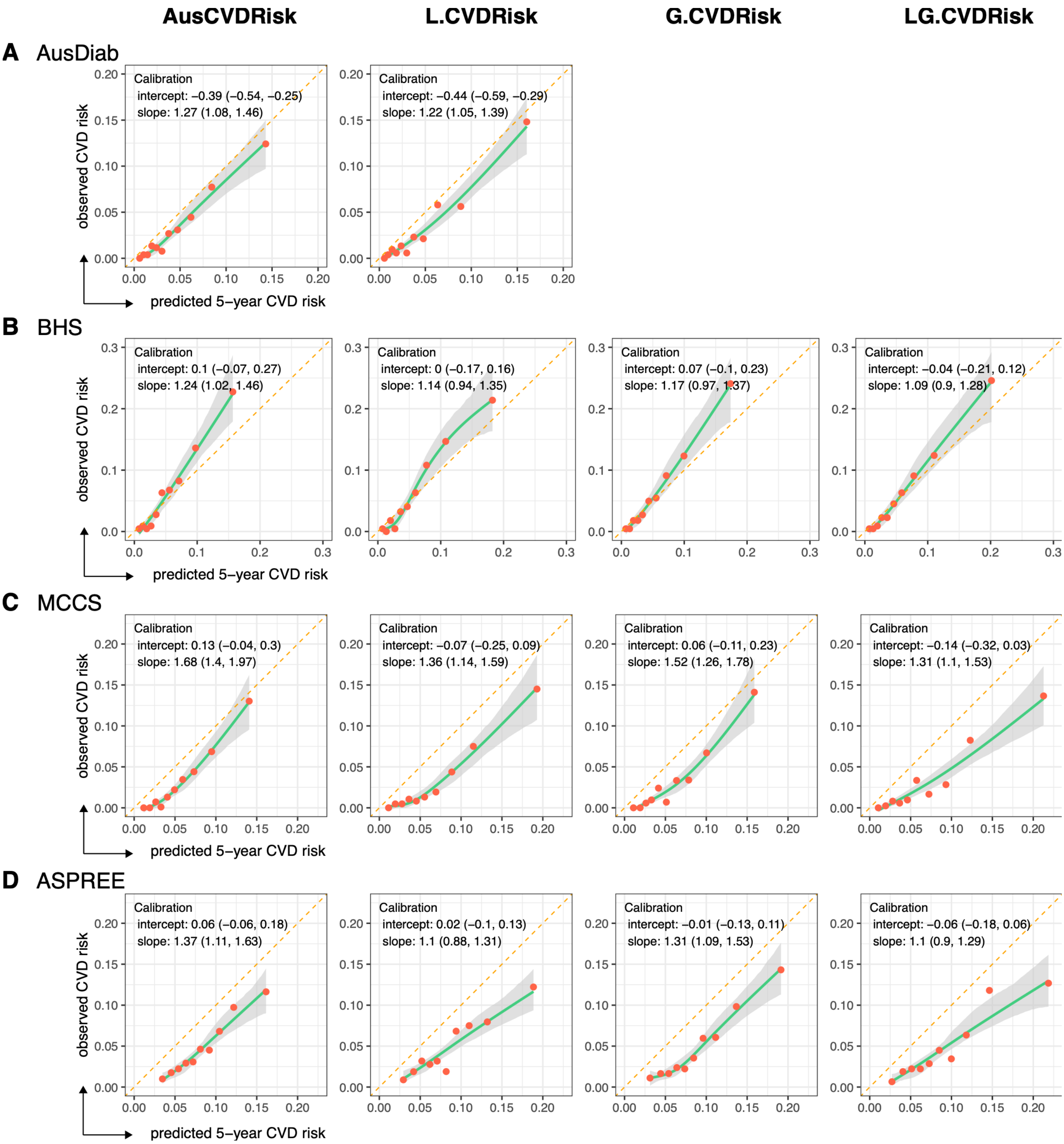


**Figure S7. Calibration plots.**

Predicted versus observed 5-year CVD risk (Kaplan–Meier estimate) across the four validation cohorts (**A–D**: AusDiab, BHS, MCCS, and ASPREE). Solid lines represent generalised additive model (GAM) smoothers, with shaded areas indicating 95% confidence intervals derived from 500 bootstrap samples. Calibration intercepts (calibration-in-the-large) and slopes, estimated using Poisson calibration models, are displayed in each panel. For MCCS and ASPREE, weighted predicted and observed risks were used to account for the case-cohort design in the visual calibration curves; calibration parameters are presented using the original case-cohort sample composition.

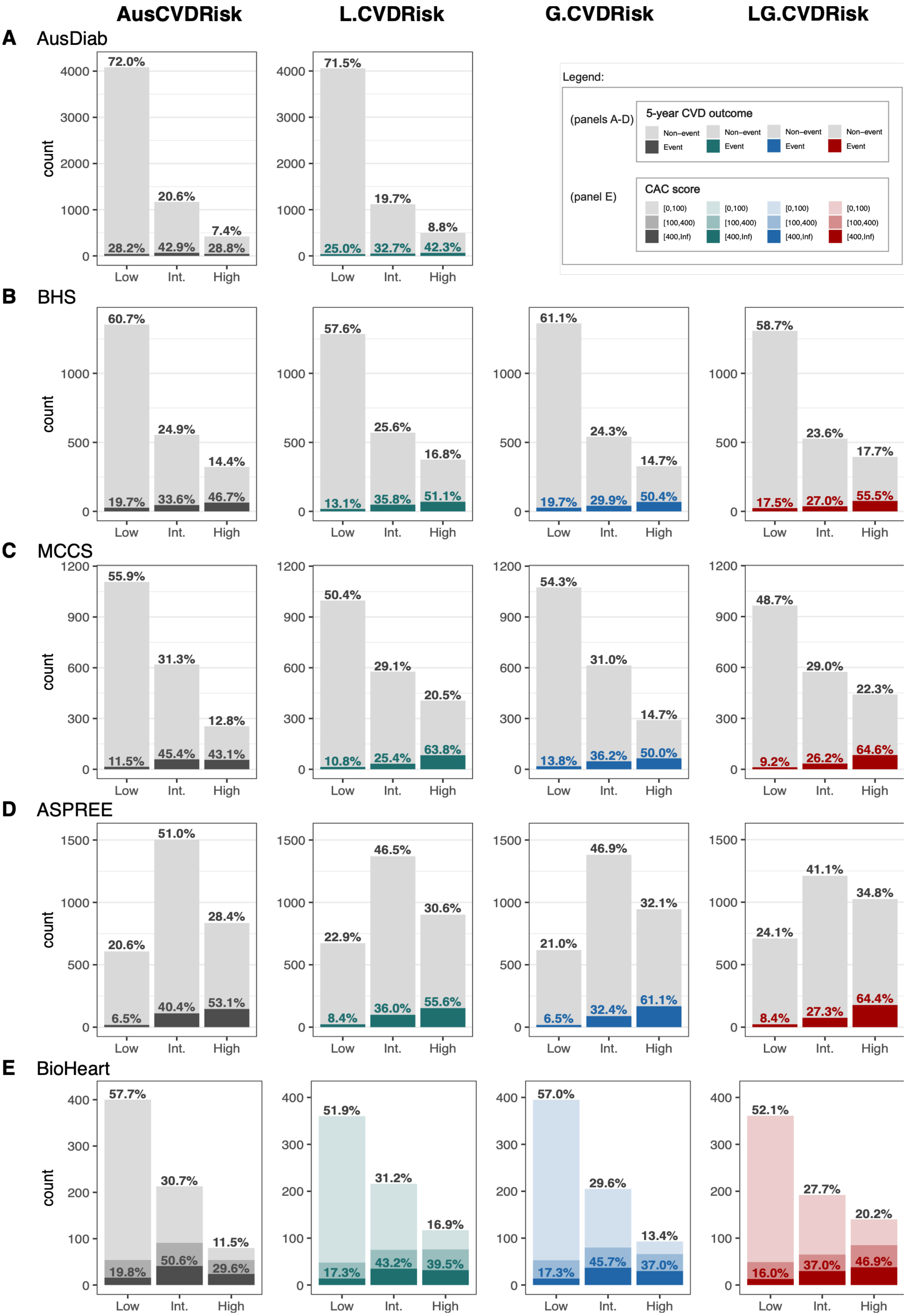


**Figure S8. CVD risk stratification using AusCVDRisk, L.CVDRisk, G.CVDRisk, and LG.CVDRisk in full cohorts (ages 45-79, or 35-79 for those with diabetes).**

Distribution of participants across low-, intermediate-, and high-risk CVD categories in five cohorts (**A-E**: AusDiab, BHS, MCCS, ASPREE, and BioHEART). Percentages of participants in each risk category are shown at the top (black text), and percentages of total CVD events (A-D) or high-CAC cases (E) in each risk category are shown at the bottom (coloured text).

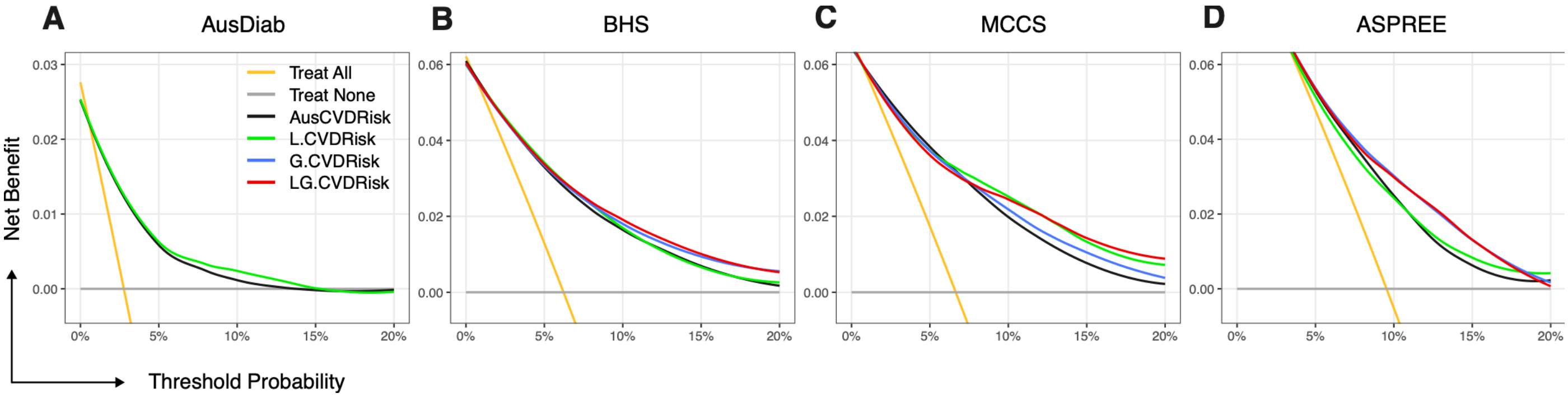


**Figure S9. Decision-curve analysis of AusCVDRisk, L.CVDRisk, G.CVDRisk, and LG.CVDRisk as stand-alone risk assessment tools.**

Unlike in the conditional testing scenario (Figure 4), all participants (ages 45-79, or 35-79 for those with diabetes) were subjected to lipidomic and/or genomic testing. Results are shown for four cohorts (**A-D**: AusDiab, BHS, MCCS, and ASPREE).
